# Impact of surrogates for insulin resistance on mortality and life expectancy in primary care: a nationwide cross-sectional study with registry linkage (LIPIDOGRAM2015)

**DOI:** 10.1016/j.lanepe.2024.101182

**Published:** 2024-12-12

**Authors:** Yang Chen, Ziyi Zhong, Ying Gue, Maciej Banach, Garry McDowell, Dimitri P. Mikhailidis, Peter P. Toth, Peter E. Penson, Tomasz Tomasik, Adam Windak, Marek Gierlotka, Tadeusz Osadnik, Agnieszka Kuras, Marcin Miga, Jacek Jozwiak, Gregory Y.H. Lip, LIPIDOGRAM2015 Investigators, B. Al-Shaer, W. Andrusewicz, M. Andrzejczuk-Rosa, E. Anusz-Gaszewska, A. Bagińska, P. Balawajder, G. Bańka, E. Barańska-Skubisz, B. Barbara Przyczyna, S. Bartkowiak, J. Bartodziej, M. Bartosiewicz, M. Basałyga, A. Batyra, A. Bąk, M. Bednarz, K. Bejnar, W. Bernacki, M. Betiuk-Kwiatkowska, S. Biegaj, M. Bień, W. Bilski, M. Biłogan, G. Biruta-Pawłowska, A. Biskup, B. Błaszczyk, H. Błaszczyk, T. BłońskaJankowska, B. Bogacka-Gancarczyk, M. Bojanowska, E. Bonda, J. Borowik-Skwarek, J. Borowska, J. Bruckner, J. Brzostek, M. Brzuchacz, M. Budzyńska, I. Bulzacka-Fugiel, J. Bulzak, K. Bunikowski, A. Cebulska, T. Celka, E. Cempel-Nowak, W. Chechliński, A. Chludzińska, D. Chmiel, M. Chmielewska, M. Cichy, A. Ciemięga, A. Ciepluch, I. Cieszyńska, B. Czajka, B. Czapla, M. Czerner, B. Czerwińska, W. Czuryszkiewicz, E. Daleka, Z. Dawid, M. Dąbrowska, R. Dąbrowska, D. Dąbrowski, M. Dąbrowski, K. Demczyszyn, A. Dębowska-Serwińska, J. Dmochowski, J. Dobrzecka-Kiwior, E. Dolanowska, H. Dolanowski, P. Dołek, M. Domagała, H. Domański, A. Doszel, D. Duda, M. Dudkowska, B. Dudziuk, P. Dybciak, M. Dymanowski, L. Dziadzio-Bolek, M. Eicke, H. El-Hassan, A. Eremus, M. Fąferek-Muller, E. Figura-Roguska, I. Fijałkowska-Kaczmarek, M. Flis, T. Florczak, M. Florczuk, E. Foryszewska-Witan, W. Frydrych, A. Fugiel, E. Futyma, A. Gaca-Jaroszewicz, I. Gajdamowicz, K. Ganczarski, A. Gatnar, M. Gers, A. Głowacki, K. Głód, J. Godula, J. Gołąb, M. Gołębiewski, E. Goszczyńska, K. Gościcka, A. GórnaHajduga, E. Górny, T. Grabowska, R. Grabowski, A. Graczyk-Duda, A. Gromow, A. Grudewicz, J. Gruszecka, A. Gruszka, J. Gryboś, J. Grzebyk, A. Grzechowiak, D. Grzesiak, T. Grześkowiak, A. Guźla, G. Hachuła, B. Hawel, H. Hiltawska, E. Honkowicz, J. Ignatowicz, K. Imielski, A. Iwaniura, A. JagiełaSzymala, M. Jalć-Sowała, A. Janczylik, E. Janisz, M. Janiszek, K. Jankiewicz-Ziobro, K. Januszewska, A. Jaremek, A. Jaros-Urbaniak, J. Jarosz, P. Jarosz, W. Jasiński, M. Jezierska-Wasilewska, T. Jędraszewski, A. Jędrzejowska, R. Józefowicz, K. Juźwin, E. Kacprzak, J. Kaczmarek-Szewczyk, M. Kaczmarzyk, R. Kandziora, C. Kaniewski, L. Karolak-Brandt, S. Kasperczyk, E. Kasperek-Dyląg, I. Kedziora, A. Kępa, J. Kiciński, J. Kielak-Al-Hosam, Ł. Kiełczawa, P. Kilimowicz, K. Kitliński, T. Kiwka, U. Klein, L. Klichowicz, A. Klimowicz, B. Klonowski, B. Kmolek, E. Kobyłko-Klepacka, A. Kocoń, A. Kolenda, E. Kollek, M. Kopeć, B. Koper-Kozikowska, J. Koralewska, M. Korczyńska, M.T. Korzeniewski, A. Kosk, K. Kotarski, E. Kowalczyk, M. Kowalczyk, I. Kowalik, B. Kozak-Błażkiewicz, M. Kozik, D. Kozłowska, E. Kozłowska, M. Kozłowska, T. Kozubski, K. Kózka, L. Kraśnik, T. Krężel, B. Krochmal, B. Król, G. Król, J. Król, T. Królikowska, H. Kruszewska, B. Krygier-Potrykus, W. Krystek, J. Krzysztoń, T. Kubicki, A. Kuczmierczyk-El-Hassan, W. Kuczyńska-Witek, D. Kujda, A. Kurowski, I. Kurzelewska-Solarz, M. Kwaczyńska, M. Kwaśniak, P. Kwaśniak, T. Kwietniewska, A. Łebek-Ordon, A. Lebiedowicz, L. Lejkowska-Olszewska, M. Lentas, A. Lesiewicz-Ksycińska, M. Limanowski, S. Łoniewski, J.A. Łopata, B. Łubianka, I. Łukasiuk, M. Łużna, M. Łysiak, B. Łysik, Z. Machowski, J. Maciaczyk-Kubiak, G. Mackiewicz-Zabochnicka, Z. Magner-Krężel, S. Majda, P. Malinowski, J. Mantyka, E. Marchlik, G. Martyna-Ordyniec, J. Marzec, M. Marzec, R. Matejko-Wałkiewicz, M. Mazur, M. Michalczak, A. Michalska-Żyłka, M. Michniewicz, D. Mika-Staniszewska, E. Mikiciuk, T. Mikołajczak, J. Milewski, E. Miller, B. Misiaszek, M. Mizik-Łukowska, E. Młyńczyk-Pokutycka, M. Mocek, M. Moczała, M. Morawska-Hermanowicz, P. Moryc, A. Moskal, S. Moskal, A. Moździerz, P. Moździerz, M. Mrozińska, K. Mrozowicz, G. Mróz, T. Munia, A. Mura, M. Muras-Skudlarska, E.Z. Murawska, Ł. Murawski, R. Murawski, R. Musielak, K. Nadaj, W. Nagarnowicz, R. Napierała, M. Niedźwiecka, A. Niemirski, J. Nikiel, M. Nosal, W. Nowacki, J. Nowak, M. Nyrka, A. Obst, J. Ochowicz, E. Ogonowska, M. Oleszczyk, A. Ołdakowski, I. Ołowniuk-Stefaniak, J. Ordowska-Rejman, M. Orliński, B. Osińska, A. Ostańska-Burian, A. Paciorkowska, U. Paczkowska, L. Paluch, L. Pałka, J. Paszko-Wojtkowska, A. Paszkowska, E. PawlakGanczarska, W. Pawlik, I. Pawłowska, M. Paździora, G. Permiakow, A. Petlic-Marendziak, T. Piasecka, E. Piaścińska, A. Piktel, A. Pilarska-Igielska, A. Piotrkowska, K. Piwowar-Klag, M. Planer, J. Plewa, P. Płatkiewicz, B. Płonczyńska, A. Podgórska, M. Polewska, B. Porębska, P. Porwoł, I. Potakowska, A. Prokop, J. Przybylski, M. Przybyła, H. Psiuk, K. Ptak, G. Puzoń, N. Rabiza, S. Rachwalik, E. Raczyńska, M. Raniszewska, A. Romanek-Kozik, A. Rosa, K. Rosa, A. Rozewicz, J. Rudzka-Kałwak, J. Rusak, D. Rutkowska, M. Rybacki, D. Rybińska, A. Rycyk-Sadowska, L. Rynda, B. Rynkiewicz, B. SadowskaKrawczyk, M. Sadowska-Zarzycka, B. Sarnecka, E. Sawalach-Tomanik, B. Sidor-Drozd, M. SiemieniakDębska, A. Sieroń, B. Siewniak-Zalewska, A. Sikora, B. Sitarska-Pawlina, J. Skorupski, I. SkrzypińskaMansfeld, J. Skubisz, R. Skwarek, M. Słodyczka, M. Smentek, K. Smolińska, B. Solarz, W. Sosnowska, B. Sroka, H. Stachura, D. Stangreciak, M. Staniak, Z. Stańczyk, D. Stańszczak-Ozga, E. Startek, M. Stefańczyk, R. Stelmach, E. Sternadel-Rączka, M. Sternik, J. Stępień, J. Stocka, M. Stokowska-Wojda, M. Studler-Karpińska, W. Suchorukow, W. Sufryd, B. Supłacz, J. Sygacz, Ł. Szczepański, J. Szkandera, J. Szłapa-Zellner, D. Szydlarska, T. Śliwa, J. Śliwka, Ł. Śmiejkowski, A. Targońska, E. Tesarska, M. Tobiasz, J. Tomaka, K. Tomalska-Bywalec, E. Tomiak, S. Topczewski, A. Trawińska, L. Trela-Mucha, D. Trojanowski, M. Trzaskowska, B. Trzcińska-Larska, A. Trznadel-Mozul, K. Ulanicka-Liwoch, M. Urbanowicz, A. Uthke-Kluzek, J. Waczyński, J. Walczak, L. Warsz, M. Wasyńczuk, U. Wąchała-Jędras, D. Wąsowicz, J. Wczysła, F. Wenda, E. Werner-Kubicka, E. Weryszko, B. Węgrzynowska, M. Wiaksa, M. Wiankowski, A. Wicherek, R. Wieczorek, R. Wiencek, G. Wienzek-Tatara, B. Wierzbicka, M. Wierzbicki, B. Wilczyńska, D. Wilmańska, P. Winiarski, A. Wiszniewska-Pabiszczak, M.B. Witkowska, J. Witzling, A. Wlaź, I. Wojtkowiak, J. Woydyłło, K. Woźniak, A. Wójtowicz, J. Wrona, M. Wrońska, H. Wujkowska, J. Wyrąbek, O. Wysokiński, R. Zakrzewski, J. Zaleska-Zatkalik, J. Zaleski, M. Zalewska-Dybciak, E. Zalewska, B. Zalewska-Uchimiak, J. Zawadzka-Krajewska, J. Zawadzki, A. Zieliński, E. Zubrycka, I. Żybort, M. Żymełka

**Affiliations:** a Liverpool Centre for Cardiovascular Science at University of Liverpool, Liverpool John Moores University and Liverpool Heart & Chest Hospital, Liverpool, United Kingdom; b Department of Cardiovascular and Metabolic Medicine, Institute of Life Course and Medical Sciences, University of Liverpool, Liverpool, United Kingdom; c Department of Musculoskeletal Ageing and Science, Institute of Life Course and Medical Sciences, University of Liverpool, Liverpool, United Kingdom; d Ciccarone Center for the Prevention of Cardiovascular Disease, Johns Hopkins University School of Medicine, Baltimore, MD 21287, USA; e Department of Preventive Cardiology and Lipidology, Medical University of Lodz (MUL), Rzgowska 281/289, Lodz 93-338, Poland; f Department of Cardiology and Adult Congenital Heart Diseases, Polish Mother’s Memorial Hospital Research Institute (PMMHRI), Rzgowska 281/289, Lodz 93-338, Poland; g Cardiovascular Research Centre, University of Zielona Gora, Zyty 28, Zielona Gora 65-046, Poland; h School of Pharmacy and Biomolecular Sciences, Liverpool John Moores University, Liverpool, United Kingdom; i Department of Clinical Biochemistry, Royal Free Hospital Campus, University College London Medical School, University College London (UCL), Pond St., London NW3 2QG, UK; j Ciccarone Center for the Prevention of Cardiovascular Disease, Johns Hopkins University School of Medicine, Baltimore, MD 21287, USA; k 14CGH Medical Center, Department of Preventive Cardiology, 101 East Miller Road, Sterling, IL 61081, USA; l Clinical Pharmacy & Therapeutics Research Group, School of Pharmacy and Biomolecular Sciences, Liverpool John Moores University, James Parsons Building, Byrom Street, Liverpool L3 3AF, UK; m Liverpool Centre for Cardiovascular Science, University of Liverpool, Brownlow Hill, Liverpool L69 7TX, UK; n Department of Cardiovascular and Metabolic Medicine, Institute of Life Course and Medical Sciences, University of Liverpool, 6 West Derby St., Liverpool L7 8TX, UK; o Department of Family Medicine, Jagiellonian University Medical College, Bochenska 4 Street, Krakow 31-061, Poland; p Department of Cardiology, Institute of Medical Sciences, University of Opole, Oleska 48, Opole 45-052, Poland; q Department of Pharmacology, Faculty of Medical Sciences in Zabrze, Medical University of Silesia, Katowice, Poland, Jordana 38 Street, Zabrze 41-808, Poland; r Multiprofile Medical Simulation Center, University of Opole, Oleska 48 Street, Opole 45-052, Poland; s Clinical University Hospital, Witosa 26 Avenue, Opole 45-401, Poland; t Department of Family Medicine and Public Health, Institute of Medical Sciences, University of Opole, Oleska 48, Opole 45-052, Poland; u Danish Center for Health Services Research, Department of Clinical Medicine, Aalborg University, Aalborg, Denmark; v Medical University of Bialystok, Bialystok 15-089, Poland

**Keywords:** Triglyceride-glucose index, TyG-adjusted body mass index, TyG-adjusted waist circumference, Insulin resistance, All-cause mortality, Premature mortality, Years of life lost

## Abstract

**Background:**

Insulin resistance (IR) is an important risk factor for multiple chronic diseases, increasing mortality and reducing life expectancy. The associations between emerging surrogates for IR, triglyceride-glucose index (TyG) and TyG-related indicators, with all-cause mortality and life expectancy in middle-aged and older patients in primary care are unclear.

**Methods:**

This study originated from the Polish primary care cohort LIPIDOGRAM2015, including patients aged ≥45 years. Baseline fasting triglycerides and fasting glucose were used to derive TyG. Other TyG-related indicators included TyG-adjusted body mass index (TyG-BMI), TyG-adjusted waist circumference (TyG-WC), TyG-adjusted waist-to-hip, and TyG-adjusted waist-to-height. In this longitudinal analysis, we assessed associations between TyG-related indicators with total all-cause mortality, premature (age at death ≤75 years) all-cause mortality and years of life lost (YLL).

**Findings:**

We included 10,688 patients (mean age 61.8 ± 9.3 years; 63.5% female). Cumulative total and premature all-cause mortality were 7.2% and 4.6%, respectively, during 5.7 years (IQR 5.6–5.7) of follow-up. Lowest (Q1) and highest quartile (Q4) of TyG-BMI and TyG-WC were associated with total all-cause mortality (second quartile [Q2]: reference; TyG-BMI: Q1: aHR 1.33, 95% CI 1.07–1.65, Q4: aHR 1.28, 95% CI 1.03–1.58; TyG-WC: Q1: aHR 1.44, 95% CI 1.14–1.82, Q4: aHR 1.29, 95% CI 1.04–1.59), similar results for premature all-cause mortality. Within age 45–80 years, compared with Q2 and third quartile, YLL were 4.49 and 5.46 years for TyG-BMI Q1 and Q4, respectively, 3.24 and 5.31 years for TyG-WC Q1 and Q4, respectively.

**Interpretation:**

TyG-BMI and TyG-WC demonstrated a U-shaped association with total and premature all-cause mortality. Low and high levels of TyG-BMI and TyG-WC were associated with reduced life expectancy. Despite the relatively short follow-up period, significant associations were still observed, but longer follow-up studies are required to further explore these relationships.

**Funding:**

Polish Lipid Association, College of Family Physician in Poland, 10.13039/100011284Valeant in Poland.

**Research in context**

**Evidence before this study**

Insulin resistance (IR) is recognized as a significant predictor of mortality in the general population, yet its assessment in clinical practice remains challenging due to the complexity, time, and cost associated with current standard methods such as the hyperinsulinemic-euglycemic clamp and the Homeostasis Model Assessment of Insulin Resistance. The triglyceride-glucose index (TyG), a recently emerging surrogate for IR, offers a simple, inexpensive, and reliable alternative that can be easily integrated into routine clinical practice without the current cost increase.

To assess the uniqueness of our study, we conducted a search in PubMed in August 2024 using terms including (1) “triglyceride-glucose index” or “TyG” or “TyG-adjusted body mass index” or “TyG-BMI” or “TyG-adjusted waist circumference” or “TyG-WC” or “TyG-adjusted waist-to-hip ratio” or “TyG-WHR” or “TyG-adjusted waist-to-height ratio” or “TyG-WHtR”, and (2) “mortality” or “all-cause mortality” or “premature mortality” or “premature all-cause mortality” or “life expectancy” or “life-expectancy” or “years of life lost”, excluding (3) journal titles containing “tyg”, without language restriction. 250 publications were retrieved, all of which focused on the general population or specific patient groups (e.g., those with diabetes mellitus, those with cardiovascular disease), with most of studies conducted in general health surveys or intensive care unit settings from the United States. Additionally, some of these studies have shown that TyG or TyG-related indicators are associated with all-cause mortality and cardiovascular mortality in different cohorts. However, no study was identified that were conducted within primary care setting, and only three specifically targeted middle-aged and older populations. Furthermore, these studies considered only all-cause mortality or specific cause mortality as the outcomes. Thus, to date, no study has specifically examined the association of TyG and TyG-related indicators (obtained by adjusting for various anthropometric measurements) with all-cause mortality, premature all-cause mortality and life expectancy in middle-aged and older adults within primary care setting.

**Added value of this study**

We investigated the associations of TyG and TyG-related related indicators with all-cause mortality, premature all-cause mortality, and years of life lost in 10,688 middle-aged and older patients within a primary care cohort from the nationwide LIPIDOGRAM2015 study in Poland. Our analysis of these patients over a follow-up period of approximately 5.7 years found that both low and high levels of TyG-BMI and TyG-WC were associated with higher all-cause mortality and premature mortality, demonstrating a U-shaped relationship. Notably, patients in the lowest and highest quartiles of TyG-BMI and TyG-WC experienced a reduction in life expectancy. By evaluating these outcomes together, our research highlights the potential impact of these indicators in identifying at-risk patients within the primary care setting. This comprehensive approach not only identifies at-risk individuals but also improves our understanding of how metabolic health impacts life expectancy.

**Implications of all the available evidence**

A direct clinical implication of our findings is the integration of TyG-related indicators into routine primary care for middle-aged and older patients. These indicators are cost-effective, easily accessible, require no additional laboratory tests, and impose no inconvenience on patients. By incorporating TyG-related indicators, clinicians in primary care can identify individuals at higher risk who might not be detected by traditional measures (e.g., glucose or low-density lipoprotein cholesterol) alone, especially in primary care settings where comprehensive metabolic assessments are essential. This has significant implications for primary care centers in Europe, where there is a growing need for practical and efficient tools to manage the metabolic health of ageing populations. Given the evidence, incorporating these simple and affordable TyG-related indicators into routine care could become a valuable tool for primary and secondary prevention of mortality risk, making them a valuable addition to the management strategies for middle-aged and older patients across European primary care settings.

## Introduction

Chronic non-communicable diseases (NCDs) are characterised by slow progression and long duration of illness,^1^ and have become the leading cause of death and disability globally,^2^ imposing a heavy human, social, and economic burden.^3^^,^^4^ NCDs are common in the middle-aged and older population and have a greater impact on the health and quality of life of these age groups.^5^ The continuous, comprehensive, and coordinated nature of primary care is consistent with the need for the management of patients with chronic diseases.^6^ Effective primary care has been associated with better health outcomes and more sustainable costs.^7^ Therefore, exploring cost-effective and reliable risk markers of mortality in the middle-aged and older populations in primary care is imperative to reduce the burden of mortality associated with NCDs and increase life expectancy.

Insulin resistance (IR) is considered to be an important predictor of a variety of chronic metabolic diseases, such as type 2 diabetes,^8^ obesity,^9^ and cardiovascular diseases.^10^^,^^11^ Previous studies have shown that IR is associated with mortality risk in the older population.^12^^,^^13^ Currently, the main indicators used for IR assessment are the hyperinsulinemic-euglycemic clamp (gold standard) and the Homeostasis Model Assessment of Insulin Resistance (HOMA-IR)^14^^,^^15^; however, these are complex, time-consuming, and expensive to calculate. Notably, a simple, inexpensive, and reliable test, the triglyceride-glucose index (TyG), has recently emerged as an attractive surrogate biomarker for IR.^16^^,^^17^ TyG is an indicator of the metabolism dysfunction calculated by glucose and triglycerides from fasting blood, making it ideal for primary care initial screening. Prior studies have reported that TyG is associated with all-cause mortality among different populations.^18^^,^^19^ Moreover, TyG adjusted by obesity indicators such as body mass index (BMI), waist circumference (WC), waist-to-height ratio (WHtR), and waist-to-hip ratio (WHR) are also potential surrogates for IR.^20^, ^21^, ^22^, ^23^ Nonetheless, while the relationship between the TyG and mortality risk has been investigated globally, as in the recent Prospective Urban Rural Epidemiology (PURE) study,^24^ evidence on both the associations between TyG-related indicators and mortality risk and the relevance of these associations specifically within middle-aged and older adults in primary care settings, remains limited.

We aimed to assess the association of TyG-related indicators for IR surrogates with all-cause mortality, premature all-cause mortality, and loss of life expectancy in a large European primary care prospective cohort.

## Methods

### Data resource and ethical approval

The cohort for this study was extracted from the Polish nationwide study LIPIOGRAM2015, which involved 438 physician-investigators in recruiting 13,724 adult patients attending primary care facilities for any medical reason in 398 primary care clinics of 16 major administrative regions in 2015–2016. Sampling, selection, and the role of the funders for the LIPIDOGRAM2015 study have been described in detail in previous publications.^25^^,^^26^ Specifically, prior to data collection, each physician-researcher underwent individual training on the study procedures and methods outlined in the research protocol. Questionnaires, blood, and saliva samples from participants were labelled with unique, identical barcodes linked to a nine-digit code. Scanned paper questionnaires and biochemical analysis data were electronically transmitted to the study database associated with each participant. Blood samples were transported to the central laboratory (Silesian Analytical Laboratories, SLA, Katowice, Poland) in temperature-controlled containers to ensure sample integrity. The LIPIDOGRAM2015 study received ethical approval from the Bioethical Commission of the Chamber of Physicians (No. K.B.Cz.-0018/2015), following the principles outlined in the Declaration of Helsinki.

### Study outcomes definitions

Study outcomes were total all-cause mortality, years of life lost (YLL), and premature all-cause mortality. Mortality follow-up data were collected from the National Health Found in Poland, using unique identification codes for all patients, and the follow-up ended in December 2021. Regarding premature mortality, although there is inequality in life expectancy between males and females, the purpose of this study was not to explore sex differences. Therefore, a uniform definition of premature mortality helps to simplify the analyses and enhance the generalisability of the results. According to the Global Burden of Disease Study 2017, the average life expectancy of the overall population in the Polish region in 2017 was about 78.0 (77.5–78.5) years, and the average life expectancy of the overall population at the age of 65 years was about 18.3 (18.0–18.7) years,^27^ with reference to the definition of premature death in the epidemic study of the European region.^28^ Premature all-cause mortality in this study was defined as age at death ≤75 years during follow-up. YLL is an indicator used to quantify the burden of premature death in a population,^29^ by calculating the difference in life expectancy between populations with different levels of TyG-related indicators, thus reflecting the impact of TyG-related indicators on life expectancy.

### Study participants

Our inclusion criteria of analysis cohort for “TyG-related indicators and total all-cause mortality/YLL” were: (i) Age ≥45 years; (ii) complete follow-up records; (iii) complete fasting triglycerides and fasting glucose; (iv) complete anthropometric measurements (height, body weight, BMI, WC, and hip circumference [HC]). On analysing TyG-related indicators and premature all-cause mortality, participants aged ≥75 years at the start of follow-up were further excluded.

### Definitions of TyG-related indicators

Serum samples from all enrolled participants were collected after fasting 12 h or more. Within 12 h following blood sample collection, fasting triglycerides (TG) were measured using direct immunological measurement on the Siemens Advia 1800 analyser and Siemens reagents (Munich, Germany). Fasting glucose was measured with Bionime glucometers and Rightest strips test (Taichung City, Taiwan).

For the anthropometric measurements: (i) no heavy clothing or shoes were worn when height (m) and weight (kg) were measured; (ii) WC (m) was measured at the level of the unclothed abdomen, specifically at the midpoint between the lower edge of the ribs and the anterosuperior iliac spine, as recommended by the World Health Organization and the International Diabetes Federation. This method is considered more accurate for assessing central obesity than the measurement at the superior border of the iliac crest in a horizontal plane, as suggested by the National Cholesterol Education Program Third Adult Treatment Panel^30^; (iii) at the level of the greater trochanter, HC (m) was measured. BMI (kg/m^2^) was calculated as weight/height.^2^ WHtR was calculated as WC/height. WHR was calculated as WC/HC.

TyG was calculated as ln [fasting glucose (mg/dL) × fasting triglycerides (mg/dL)/2]. TyG-BMI was calculated as TyG × BMI, TyG-WC was calculated as TyG × WC, TyG-WHtR was calculated as TyG × WHtR, and TyG-WHR was calculated as TyG × WHR.

### Covariates definitions

In this analysis, we included basic demographics (age, sex, level of education, place of residence), lifestyle factors (smoking status, alcohol consumption, regular physical activity, use of antiatherogenic diet), comorbidities (diabetes mellitus [DM], hypertension (HTN), chronic kidney disease [CKD], myocardial infarction [MI], stroke, atrial fibrillation [AF]), use of medications (treatment of dyslipidaemia, treatment of DM, treatment of HTN, antiplatelet treatment, anticoagulant treatment), and laboratory results (high-density lipoprotein cholesterol [HDL-C], low-density lipoprotein cholesterol [LDL-C], total cholesterol [TC], TG). Level of education was defined as “primary and vocational education” and “secondary and higher education”. Place of residence was defined as “rural residence” and “urban residence”. Smoking status was defined as “never smoke” and “past/current smoke”. Alcohol consumption was defined as “never drink” as well as “moderate and heavy alcohol intake”. Levels of physical activity were defined as “regular physical activity” (e.g., Aerobic exercise, playing team games, doing housework or household chores etc.) for 2.0–2.5 h per week” and “no regular physical activity” for not meeting the criteria of regular physical activity. The use of antiatherogenic diet was defined as “use antiatherogenic (hypolipemic, hypoglycemic, hypotensive) diet”,^31^ and “no use”. The information on comorbidities and use of medications was obtained from questionnaires and validated by primary care physicians. Besides, HDL-C, LDL-C, and TC were measured using direct immunological measurement on the Siemens Advia 1800 analyser and Siemens reagents (Munich, Germany).

### Statistical analysis

Normally distributed continuous variables were expressed as means and standard deviations, and differences among groups were evaluated by one-way analysis of variance. Non-normally distributed continuous variables were expressed as median and interquartile range (IQR), and differences among groups were assessed by the Kruskal–Wallis test. Categorical variables were expressed as counts and percentages, and differences among groups were assessed by the Chi-square or Fisher’s test.

Due to the difference in cohort sizes for the different outcomes, the interquartile ranges for the TyG-related indicators also differed (Supplementary Table S1). We plotted Kaplan-Meyer curves for the follow-up times of the different TyG-related indicators groups (divided by quartile level) and performed log-rank tests to compare the differences in cumulative risk among groups. Subsequently, we plotted restricted cubic spline (RCS) curves between continuous TyG-related indicators and all-cause mortality, adjusting for confounders.

To assess the association among different levels of TyG-related indicators and outcomes (total and premature all-cause mortality), regarding the group with the lowest risk of all-cause mortality based on the RCS curves as the reference group, we used multivariable Cox proportional hazards models to calculate adjusted hazard ratios (aHR) and 95% confidence intervals (CI), adjusted for confounders. Correlation and multicollinearity tests between potential confounders were pre-tested to ensure that the adjustment variables included within the model were not significantly correlated (Pearson correlation coefficient [r] >0.8) and strongly multicollinear (variance inflation factor >10). Based on Supplementary Figs. S1 and S2, TC was not included in the adjustment model as the strong correlation between LDL-C and TC (r = 0.89). Thus, we used four models for adjustment as follows: Model 1 was not adjusted for confounders; Model 2 was adjusted for age, sex, level of education, place of residence, smoking status, alcohol consumption, regular physical activity, and use of antiatherogenic diet; Model 3 was further adjusted for comorbidities including DM, hypertension (HTN), CKD, MI, stroke, and AF based on Model 2; Model 4 was further adjusted for LDL-C and HDL-C based on Model 3. To ensure the stability and interpretability of the model, multiple covariance test was conducted for all the variables included in the Model 4 which demonstrated that the variance inflation factor values of all variables were less than 8, indicating that there was no significant multicollinearity among these variables. The proportional hazards assumption was checked utilising Schoenfeld residuals.^32^ If the *P*-values for all variables included in Model 4, as well as for the overall model, were greater than 0.05, the proportional hazards assumption was considered to be satisfied (Supplementary Figs. S3–S12).

For plotting the RCS curves, we used Model 4 for adjustment and the knot of RCS was set to 4. Then, if a non-linear relationship was found, a two-step recursive algorithm (as detailed in the Supplementary Methods) was performed to calculate the inflection points between TyG-related indicators and total all-cause mortality, respectively, and to perform a threshold effect analysis between TyG-related indicators and total all-cause mortality by using Cox proportional hazards model on each side of the inflection points.^33^ Furthermore, we performed a multivariable Cox proportional hazards model adjusted for Model 4 in different subgroups (age ≥65 and age <65 years; male and female) to assess the association of TyG-related indicators with total and premature all-cause mortality.

In addition, we conducted four sensitivity analyses on the TyG-related indicators significantly associated with total and premature all-cause mortality outcomes. First, we assessed whether TyG-related indicators outperformed corresponding anthropometric measurements in predicting total all-cause mortality by calculating the continuous net reclassification improvement (NRI) and integrated discrimination improvement (IDI) after incorporating corresponding anthropometric measurements and TyG-related indicators into the basic model (including age, sex, level of education, place of residence, smoking status, and alcohol consumption, regular physical activity, use of antiatherogenic diet, DM, HTN, CKD, MI, stroke, AF, LDL-C and HDL-C), respectively. Second, we further added the use of medications (including treatment of dyslipidaemia, treatment of DM, treatment of HTN, antiplatelet treatment, anticoagulant treatment) to Model 4 as confounders to evaluate whether the associations between TyG-related indicators with total and premature all-cause mortality outcomes remained stable. The proportional hazards assumption was checked (Supplementary Figs. S13–S16). Third, considering that some of the variables, especially disease comorbidities, might be mediators in the causal pathways from IR to total all-cause mortality, we applied a causal mediation analysis to explore whether the relationships between TyG-related indicators and total all-cause mortality were mediated by comorbidities (DM, HTN, CKD, MI, stroke, AF).

### Role of the funding source

The LIPIDOGRAM2015 Project and the LIPIDOGRAM2015 Investigators received non-material support from the Polish Lipid Association (PoLA) and the College of Family Physicians in Poland (CFPIP). The present study was funded by an unrestricted educational grant from Valeant (Warsaw, Poland). Valeant had no role in the study design, data analysis, data interpretation, or writing of the report.

## Results

### Baseline characteristics

We ultimately included 10,688 participants who met the inclusion criteria (the flowchart of this study is provided in Supplementary Fig. S17). The mean age of participants was 61.8 (9.3) years, and the median follow-up time was 5.7 (IQR 5.6–5.7) years (58594.0 person-years of follow-up). There were 759 (7.2%) total all-cause mortality events and 460 (4.3%) premature all-cause mortality events. As shown in Table 1, compared with the survivors group, participants in the death group were older, had higher BMI and WC, smaller proportion of higher education, and higher proportion of comorbidities (DM, HTN, CKD, MI, stroke, AF). Additionally, the baseline characteristics of the different levels of TyG-related indicators are given in Supplementary Tables S2–S6.

**Table 1:** Baseline characteristics of the survivors and non-survivors groups.

Characteristic	All	Survivors	Non-survivors	*P*-value
N	10,688	9921	767	
Age, years	61.27 (54.89, 67.69)	60.66 (54.43, 66.95)	69.22 (63.73, 77.31)	<0.0001
Female, n (%)	6789 (63.5)	6443 (64.9)	346 (45.1)	<0.0001
BMI, kg/m^2^	28.50 (25.50, 31.80)	28.40 (25.50, 31.60)	29.20 (25.30, 32.70)	0.0085
WC, m	0.96 (0.88, 1.05)	0.96 (0.87, 1.04)	1.01 (0.91, 1.10)	<0.0001
WHtR	0.58 (0.53, 0.63)	0.58 (0.53, 0.63)	0.60 (0.55, 0.66)	<0.0001
WHR	0.90 (0.84, 0.96)	0.90 (0.84, 0.96)	0.93 (0.88, 0.99)	<0.0001
Higher education, n (%)	5621 (52.6)	5365 (54.1)	256 (33.4)	<0.0001
Urban residence, n (%)	5502 (51.5)	5095 (51.4)	407 (53.1)	0.3684
Smoking status, n (%)				0.0075
Never	8952 (83.8)	8336 (84.0)	616 (80.3)	
Past/current	1736 (16.2)	1585 (16.0)	151 (19.7)	
Alcohol Consumption, n (%)				0.1100
Never	3902 (36.5)	3601 (36.3)	301 (39.2)	
Moderate/high	6786 (63.5)	6320 (63.7)	466 (60.8)	
Regular physical activity, n (%)	4189 (39.2)	3996 (40.3)	193 (25.2)	<0.0001
Use of antiatherogenic diet, n (%)	7412 (69.3)	6901 (69.6)	511 (66.6)	0.0948
Comorbidity, n (%)				
Diabetes mellitus	1739 (16.3)	1504 (15.2)	235 (30.6)	<0.0001
Hypertension	6236 (58.3)	5676 (57.2)	560 (73.0)	<0.0001
Chronic kidney disease	338 (3.2)	289 (2.9)	49 (6.4)	<0.0001
Myocardial infarction	607 (5.7)	490 (4.9)	117 (15.3)	<0.0001
Stroke	286 (2.7)	237 (2.4)	49 (6.4)	<0.0001
Atrial fibrillation	687 (6.4)	563 (5.7)	124 (16.2)	<0.0001
Use of Medications, n (%)				
Treatment of Dyslipidaemia	3837 (35.9)	3509 (35.4)	328 (42.8)	<0.0001
Treatment of diabetes mellitus	1635 (15.3)	1414 (14.3)	221 (28.8)	<0.0001
Treatment of hypertension	6005 (56.2)	5462 (55.1)	543 (70.8)	<0.0001
Antiplatelet treatment	1552 (14.5)	1380 (13.9)	172 (22.4)	<0.0001
Anticoagulant treatment	845 (7.9)	699 (7.0)	146 (19.0)	<0.0001
Laboratory results				
Triglycerides, mg/dL	126.30 (93.60, 175.20)	126.40 (93.60, 175.35)	123.80 (93.30, 169.80)	0.5389
Glucose, mg/dL	100.00 (92.00, 111.00)	100.00 (92.00, 111.00)	103.00 (95.00, 123.00)	<0.0001
LDL-C, mg/dL	128.00 (100.00, 157.00)	129.00 (101.00, 158.00)	109.00 (84.00, 142.00)	<0.0001
HDL-C, mg/dL	52.70 (43.80, 63.40)	53.10 (44.20, 63.80)	48.00 (39.30, 58.20)	<0.0001
TC, mg/dL	201.70 (172.30, 232.50)	203.00 (174.20, 233.60)	180.80 (150.70, 216.60)	<0.0001
TyG Related Indicators				
TyG	8.77 (8.43, 9.16)	8.76 (8.43, 9.15)	8.82 (8.47, 9.20)	0.0136
TyG-BMI	251.39 (219.42, 286.65)	250.83 (219.41, 285.85)	258.17 (220.23, 297.73)	0.0025
TyG-WC	8.47 (7.51, 9.43)	8.44 (7.49, 9.40)	8.93 (7.88, 10.00)	<0.0001
TyG-WHtR	5.09 (4.55, 5.68)	5.07 (4.54, 5.66)	5.35 (4.79, 6.02)	<0.0001
TyG-WHR	7.94 (7.25, 8.66)	7.91 (7.22, 8.62)	8.23 (7.63, 8.96)	<0.0001

BMI, body mass index; HDL-C, high-density lipoprotein cholesterol; LDL-C, low-density lipoprotein cholesterol; TC, total cholesterol; TyG, triglyceride-glucose index; WC, waist circumference; WHtR, waist-to-height ratio; WHR, waist-to-hip ratio.

### Overall survival for different levels of TyG-related indicators

**Fig. 1:**
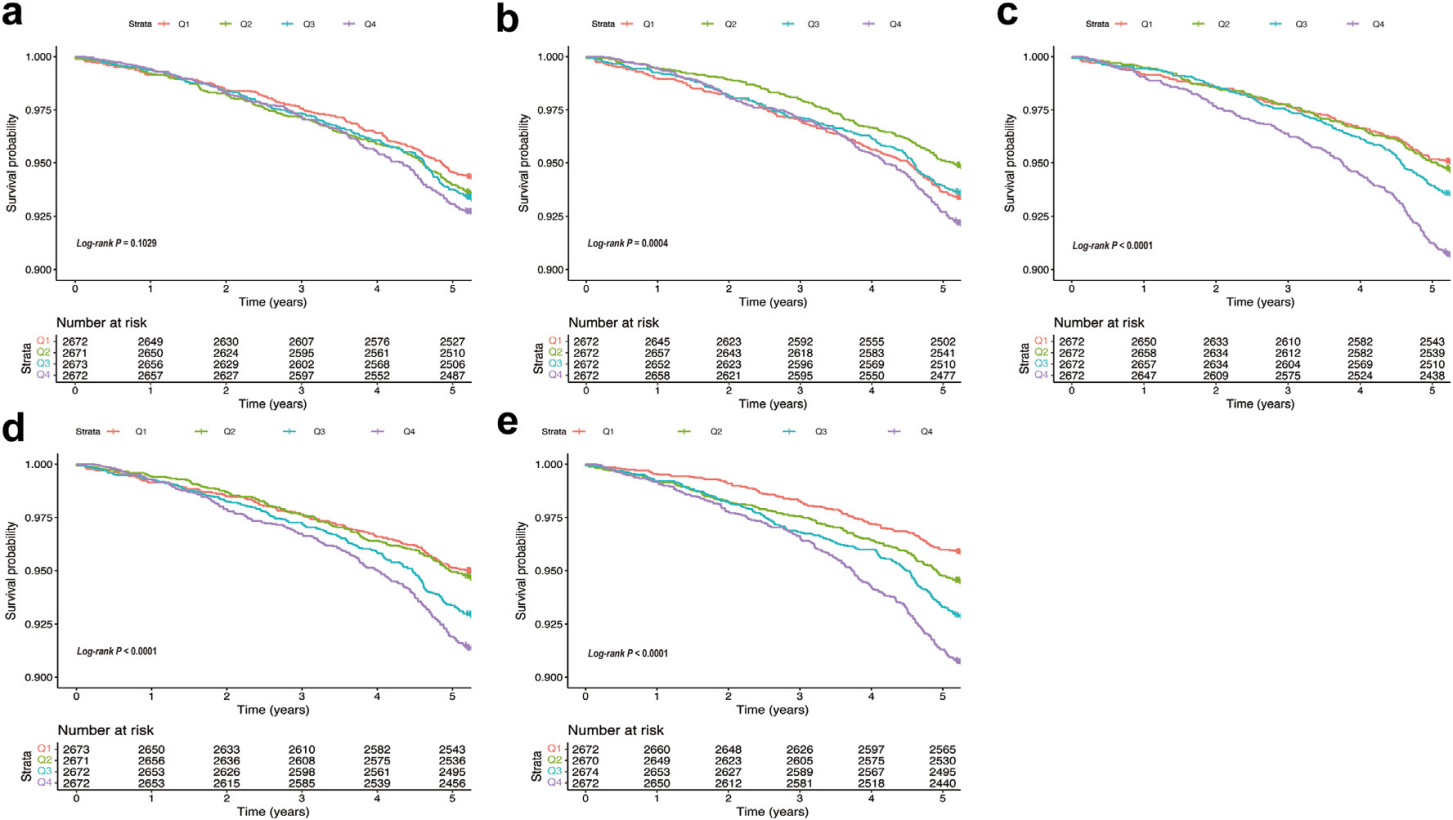
**Kaplan–Meier analysis of overall survival based on quartiles of TyG (a), TyG-BMI (b), TyG-WC (c), TyG-WHtR (d), and TyG-WHR (e) in whole cohort.** Abbreviations: BMI, body mass index; TyG, triglyceride-glucose index; WC, waist circumference; WHtR, waist-to-height ratio; WHR, waist-to-hip ratio.

Fig. 1 shows the Kaplan–Meier survival analysis curves for total all-cause mortality during follow-up for quartile groups of TyG-related indicators. Apart from TyG (*log-rank P* = 0.1029), there were statistically significant differences in TyG-BMI, TyG-WC, TyG-WHtR, and TyG-WHR (TyG-BMI: *log-rank P* = 0.0004; others: *log-rank P* < 0.0001). Among all indicators, the fourth quartile (Q4) had the lowest survival rate.

The second quartile (Q2) of TyG-BMI had the lowest mortality, while the first quartile of TyG-WHR had the lowest mortality. The first and second quartiles (Q1, Q2) of TyG-WC or TyG-WHtR had similar trends in survival rates and significantly lower mortality than the third or fourth quartiles (Q3, Q4).

### Association of TyG-related indicators with total all-cause mortality

**Fig. 2:**
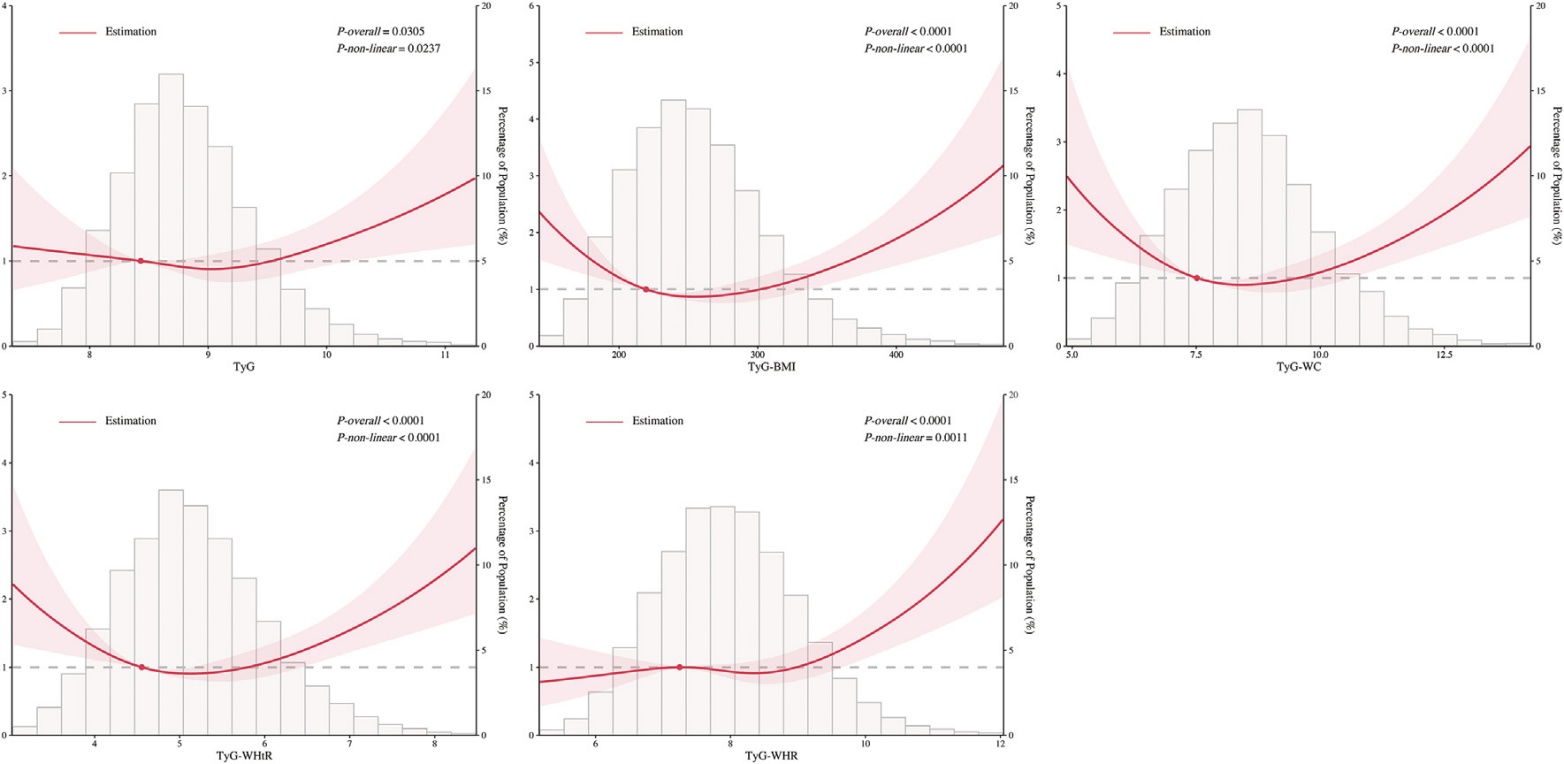
**Associations between TyG-related indicators and total all-cause mortality with restricted cubic splines.** The Cox proportional hazards model was adjusted for age, sex, level of education, place of residence, smoking status, alcohol consumption, regular physical activity, use of antiatherogenic diet, diabetes mellitus, hypertension, chronic kidney disease, myocardial infarction, stroke, atrial fibrillation, low-density lipoprotein cholesterol, and high-density lipoprotein cholesterol. Abbreviations: BMI, body mass index; TyG, triglyceride-glucose index; WC, waist circumference; WHtR, waist-to-height ratio; WHR, waist-to-hip ratio.

According to the multivariate-adjusted RCS analysis (Fig. 2), except TyG, TyG-related indicators showed significant non-linear associations with total all-cause mortality (TyG-WHR: *P-non-linear* = 0.0011; others: *P-non-linear* < 0.0001). TyG-BMI, TyG-WC and TyG-WHtR were associated with total all-cause mortality in a U-shaped association, whereas TyG and TyG-WHR showed a J-shaped correlation.

**Fig. 3:**
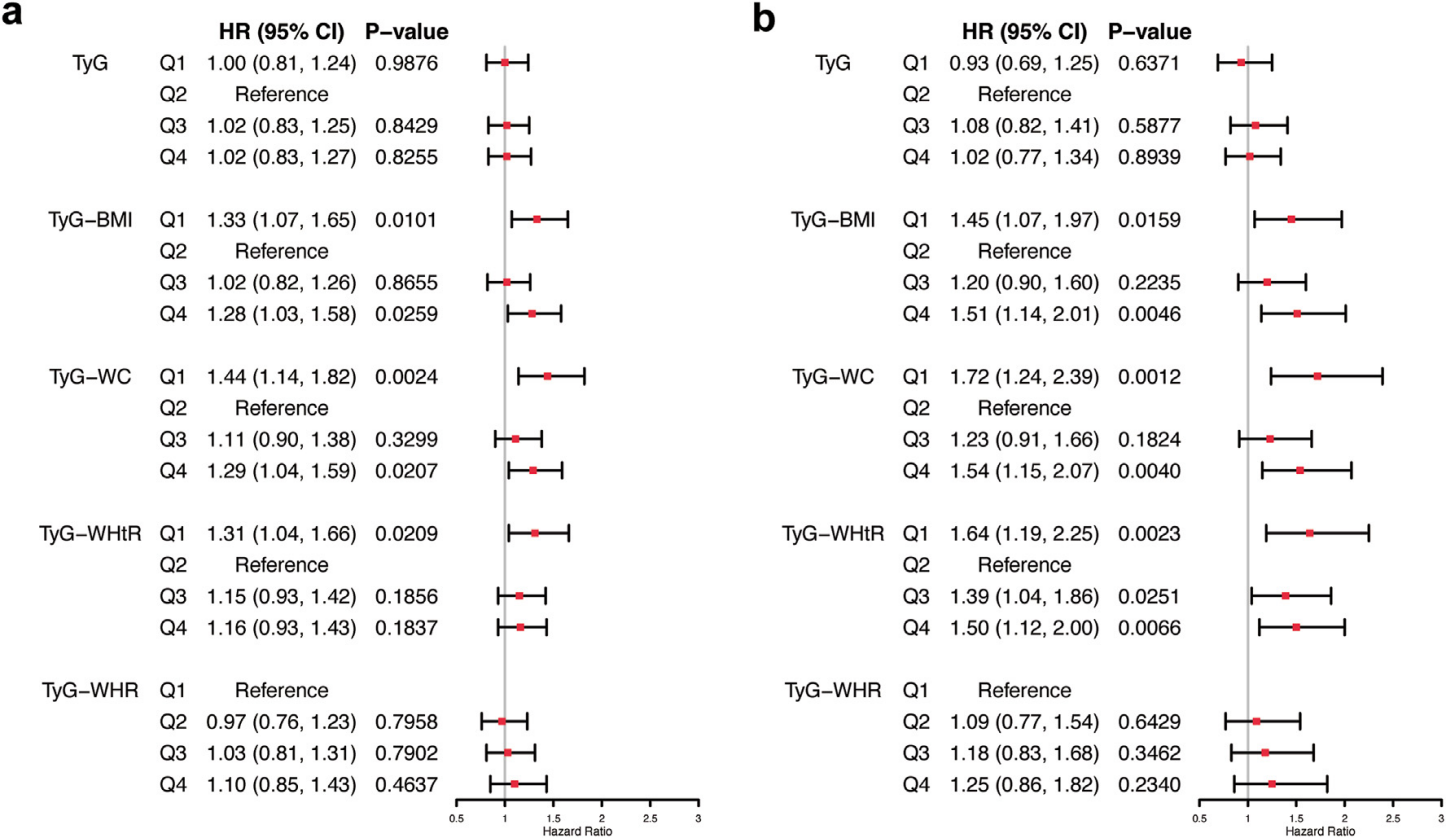
**Multivariable Cox proportional hazard regression model for total (a) and premature (b) all-cause mortality.** The Cox proportional hazards model was adjusted for Model 4, including age, sex, level of education, place of residence, smoking status, alcohol consumption, regular physical activity, use of antiatherogenic diet, diabetes mellitus, hypertension, chronic kidney disease, myocardial infarction, stroke, atrial fibrillation, low-density lipoprotein cholesterol, and high-density lipoprotein cholesterol. Abbreviations: BMI, body mass index; CI, confidence interval; HR, hazard ratio; TyG, triglyceride-glucose index; WC, waist circumference; WHtR, waist-to-height ratio; WHR, waist-to-hip ratio.

Based on the results of Cox proportional hazards model fully adjusted for confounders (Table 2 and Fig. 3), TyG-BMI, TyG-WC, and TyG-WHtR were associated with total all-cause mortality (TyG-BMI: Q2 as the reference group, Q1: aHR 1.33, 95% CI 1.07–1.65, *P* = 0.0101, Q4: aHR 1.28, 95% CI 1.03–1.58, *P* = 0.0259; TyG-WC: Q2 as the reference group, Q1: aHR 1.44, 95% CI 1.14–1.82, *P* = 0.0024, Q4: aHR 1.29, 95% CI 1.04–1.59, *P* = 0.0207; TyG-WHtR: Q2 as the reference group, Q1: aHR 1.31, 95% CI 1.04–1.66, *P* = 0.0209), whereas TyG and TyG-WHR were not associated with total all-cause mortality.

**Table 2:** Univariable and multivariable Cox proportional hazards regression model for all-cause mortality.

Variables	Model 1		Model 2		Model 3		Model 4	
	HR (95% CI)	*P*-value	HR (95% CI)	*P*-value	HR (95% CI)	*P*-value	HR (95% CI)	*P*-value
TyG								
Q1	0.91 (0.74, 1.13)	0.3994	0.99 (0.80, 1.22)	0.9228	1.02 (0.83, 1.26)	0.8704	1.00 (0.81, 1.24)	0.9876
Q2	Reference		Reference		Reference		Reference	
Q3	1.09 (0.89, 1.33)	0.3895	1.09 (0.89, 1.33)	0.3903	1.03 (0.84, 1.26)	0.7640	1.02 (0.83, 1.25)	0.8429
Q4	1.16 (0.95, 1.42)	0.1357	1.17 (0.96, 1.43)	0.1234	1.04 (0.85, 1.28)	0.6836	1.02 (0.83, 1.27)	0.8255
TyG-BMI								
Q1	1.25 (1.01, 1.54)	0.0415	1.28 (1.03, 1.59)	0.0236	1.31 (1.06, 1.62)	0.0139	1.33 (1.07, 1.65)	0.0101
Q2	Reference		Reference		Reference		Reference	
Q3	1.23 (1.00, 1.52)	0.0552	1.12 (0.90, 1.38)	0.3065	1.04 (0.84, 1.29)	0.7198	1.02 (0.82, 1.26)	0.8655
Q4	1.55 (1.26, 1.90)	<0.0001	1.54 (1.26, 1.90)	<0.0001	1.33 (1.07, 1.64)	0.0093	1.28 (1.03, 1.58)	0.0259
TyG-WC								
Q1	0.95 (0.76, 1.20)	0.6868	1.37 (1.09, 1.73)	0.0079	1.40 (1.11, 1.77)	0.0045	1.44 (1.14, 1.82)	0.0024
Q2	Reference		Reference		Reference		Reference	
Q3	1.32 (1.07, 1.63)	0.0101	1.18 (0.95, 1.46)	0.1322	1.12 (0.91, 1.39)	0.2921	1.11 (0.90, 1.38)	0.3299
Q4	1.89 (1.55, 2.30)	<0.0001	1.55 (1.26, 1.89)	<0.0001	1.33 (1.08, 1.64)	0.0081	1.29 (1.04, 1.59)	0.0207
TyG-WHtR								
Q1	0.95 (0.76, 1.20)	0.6804	1.25 (1.00, 1.58)	0.0520	1.29 (1.03, 1.62)	0.0294	1.31 (1.04, 1.66)	0.0209
Q2	Reference		Reference		Reference		Reference	
Q3	1.38 (1.12, 1.70)	0.0026	1.22 (0.99, 1.51)	0.0594	1.17 (0.95, 1.44)	0.1451	1.15 (0.93, 1.42)	0.1856
Q4	1.72 (1.41, 2.10)	<0.0001	1.39 (1.14, 1.70)	0.0013	1.20 (0.97, 1.47)	0.0945	1.16 (0.93, 1.43)	0.1837
TyG-WHR								
Q1	Reference		Reference		Reference		Reference	
Q2	1.36 (1.08, 1.72)	0.0104	1.03 (0.81, 1.31)	0.7951	0.97 (0.77, 1.24)	0.8236	0.97 (0.76, 1.23)	0.7958
Q3	1.82 (1.45, 2.27)	<0.0001	1.14 (0.90, 1.43)	0.2786	1.04 (0.82, 1.31)	0.7737	1.03 (0.81, 1.31)	0.7902
Q4	2.31 (1.87, 2.87)	<0.0001	1.33 (1.05, 1.68)	0.0189	1.11 (0.87, 1.42)	0.3856	1.10 (0.85, 1.43)	0.4637

BMI, body mass index; CI, confidence interval; HR, hazard ratio; TyG, triglyceride-glucose index; WC, waist circumference; WHtR, waist-to-height ratio; WHR, waist-to-hip ratio.

Model 1: Unadjusted.

Model 2: Adjusted for age, sex, level of education, place of residence, smoking status, and alcohol consumption, regular physical activity, use of antiatherogenic diet.

Model 3: Model 2 further adjusted for diabetes mellitus, hypertension, chronic kidney disease, myocardial infarction, stroke, atrial fibrillation.

Model 4: Model 3 further adjusted for low-density lipoprotein cholesterol and high-density lipoprotein cholesterol.

Since all the TyG-related indicators had significant nonlinear relationships with total all-cause mortality, Supplementary Table S7 provides the inflection points of the TyG-related indicators for total all-cause mortality and the results of the corresponding threshold effect analysis. For instance, the inflection point of TyG-WC for total all-cause mortality was 7.66, and when TyG-WC < 7.66, for each 1-unit increase in TyG-WC, the aHR (95% CI) for total all-cause mortality was 0.63 (0.53, 0.75), *P* < 0.0001; whereas when TyG-WC ≥ 7.66, for each 1-unit increase in TyG-WC, the aHR (95% CI) for total all-cause mortality was 1.17 (1.10, 1.25), *P* < 0.0001.

### Associations of TyG-related indicators with premature all-cause mortality

According to the results of Cox proportional hazards model fully adjusted for all confounders (Fig. 3), TyG-BMI, TyG-WC and TyG-WHtR were associated with premature all-cause mortality (TyG-BMI: Q2 as the reference group, Q1: aHR 1.45, 95% CI 1.07–1.97, *P* = 0.0159, Q4: aHR 1.51, 95% CI 1.14–2.01, *P* = 0.0046; TyG-WC: Q2 as the reference group, Q1: aHR 1.72, 95% CI 1.24–2.39, *P* = 0.0012, Q4: aHR 1.54, 95% CI 1.15–2.07, *P* = 0.0040; TyG-WHtR: Q2 as the reference group, Q1: aHR 1.64, 95% CI 1.19–2.25, *P* = 0.0023, Q3: aHR 1.39, 95% CI 1.04–1.86, *P* = 0.0251, Q4: aHR 1.50, 95% CI 1.12–2.00, *P* = 0.0066), whereas TyG and TyG-WHR were not associated with premature all-cause mortality.

### Subgroup analysis

Subgroup analyses showed that TyG-BMI and TyG-WHtR in females, and TyG-WC in both sexes were associated with total all-cause mortality (Supplementary Fig. S18), but there was only a significant interaction between TyG-BMI and sex (TyG-BMI: *P for interaction* = 0.0355; TyG-WC: *P for interaction* = 0.7332; TyG-WHtR: *P for interaction* = 0.1852). Additionally, according to Supplementary Fig. S19, subgroup analyses with age groups indicated that TyG-BMI, TyG-WC, and TyG-WHtR were associated with total all-cause mortality in the 45 ≤ age <65 years group, whereas TyG-WC was related to it in the age ≥ 65 years group, while there was no significant interaction between TyG-BMI or TyG-WC and age group (TyG-BMI: *P for interaction* = 0.3279; TyG-WC: *P for interaction* = 0.2945; TyG-WHtR: *P for interaction* = 0.1662).

Subgroup analyses showed that in males TyG-BMI, TyG-WC and TyG-WHtR were associated with premature all-cause mortality, while in females TyG-BMI and TyG-WC were related to it (Supplementary Fig. S20), but there were no significant interactions between these TyG-related indicators and sex (TyG-BMI: *P for interaction* = 0.8740; TyG-WC: *P for interaction* = 0.0728; TyG-WHtR: *P for interaction* = 0.5761). Moreover, according to Supplementary Fig. S21, subgroup analyses with age groups indicated that TyG-BMI, TyG-WC, and TyG-WHtR were associated with premature all-cause mortality in the 45 ≤ age <65 years group, whereas only TyG-WC was related to it in the age ≥ 65 years group, while there was no significant interaction between these TyG-related indicators and age group (TyG-BMI: *P for interaction* = 0.3880; TyG-WC: *P for interaction* = 0.5968; TyG-WHtR: *P for interaction* = 0.3675).

### Sensitivity analysis

We assessed whether TyG-BMI and TyG-WC outperformed BMI and WC in predicting total all-cause mortality using NRI and IDI (Supplementary Table S8). Compared to adding BMI, adding TyG-BMI to the basic model significantly improved the prediction of total all-cause mortality (continuous NRI: 11.25%, *P* = 0.0026; IDI: 0.22%, *P* = 0.0118). Similarly, adding TyG-WC improved risk reclassification compared to adding WC (continuous NRI: 10.43%, *P* = 0.0052; IDI: 0.20%, *P* = 0.0034).

After adjusting for the use of medications, the associations between TyG-BMI, TyG-WC, and mortality outcomes remained significant (Supplementary Table S9). The Q1 and Q4 of both TyG-BMI and TyG-WC were associated with a higher risk of total all-cause mortality (TyG-BMI: Q2 as the reference group, Q1: aHR 1.34, 95% CI 1.08–1.66, *P* = 0.0088, Q4: aHR 1.28, 95% CI 1.03–1.59, *P* = 0.0252; TyG-WC: Q2 as the reference group, Q1: aHR 1.45, 95% CI 1.14–1.83, *P* = 0.0022, Q4: aHR 1.30, 95% CI 1.05–1.60, *P* = 0.0176). Similarly, these associations were observed for premature all-cause mortality.

The mediation analysis results, presented in Supplementary Table S10, showed that none of the comorbidities included in this analysis significantly mediated the relationships between TyG-BMI or TyG-WC and total all-cause mortality.

### Exploration interaction analysis of TyG-BMI and TyG-WC in males and females

Since TyG-BMI, TyG-WC, and TyG-WHtR were significantly associated with total all-cause mortality, but TyG-WHtR showed statistical significance only in Q1 compared with Q2, thus TyG-BMI and TyG-WC were selected for further analyses. However, as there was a significant interaction between categorised TyG-BMI and sex, and WC has different categorisation criteria due to physiological differences in body fat distribution between sexes, we additionally performed RCS and interaction analyses in males and females. Supplementary Fig. S22 presents that the direction and trend of the effects of TyG-BMI and TyG-WC were essentially similar in both sexes, and there were no significant interactions (TyG-BMI: *P for interaction* = 0.3437; TyG-WC: *P for interaction* = 0.1121), implying that they are reliable and generalisable in cross-sex applications.

### YLL in groups with different levels of TyG-BMI and TyG-WC

**Fig. 4:**
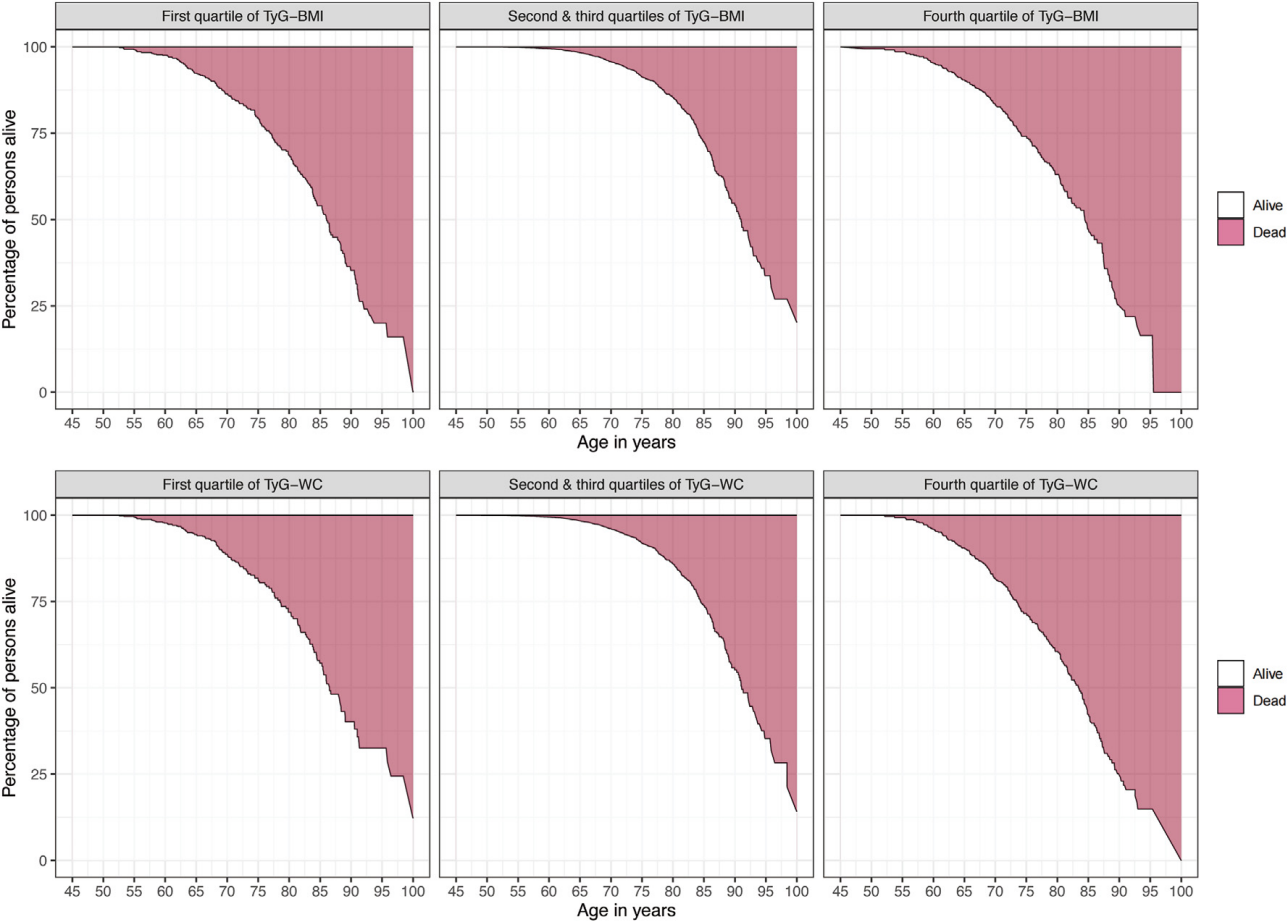
**Percentage of survivors in the 45- to 100-year age range in the first, fourth quartiles, and second-third composite quartile groups for TyG-BMI and TyG-WC in whole cohort.** Abbreviations: BMI, body mass index; TyG, triglyceride-glucose index; WC, waist circumference.

**Fig. 5:**
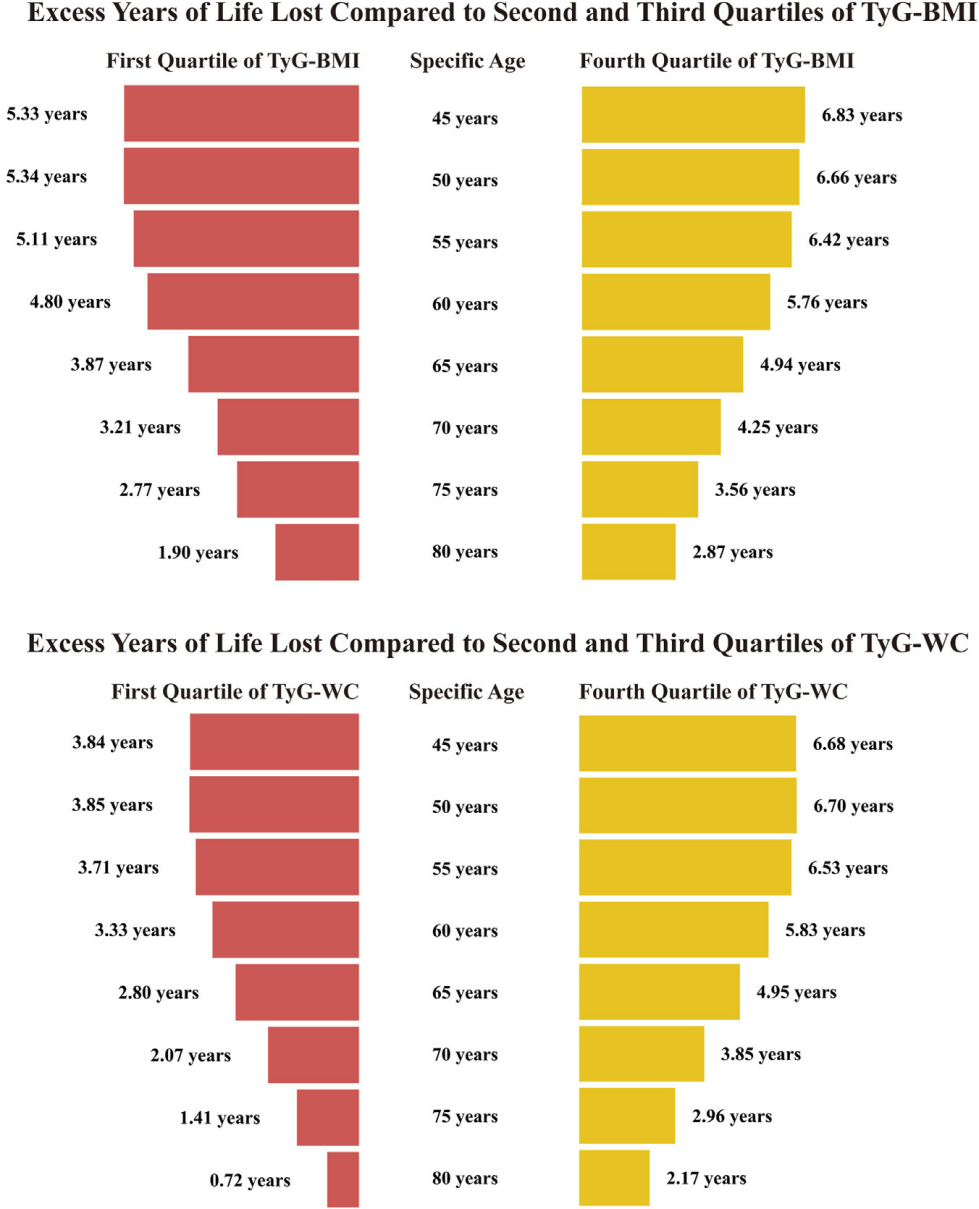
**Age-specific excess years of life lost in the first and fourth quartiles compared to the second and third composite quartiles for TyG-BMI and TyG-WC.** Abbreviations: BMI, body mass index; TyG, triglyceride-glucose index; WC, waist circumference.

Since the Cox proportional hazards model showed no significant difference in the risk of total all-cause mortality between the Q2 and Q3 of TyG-BMI or TyG-WC, so in this section, we combined these two groups. Fig. 4 illustrates the curves of YLL at different ages for different quartiles of TyG-BMI and TyG-WC between age 45 and 100 years. Additionally, we calculated the YLL and 95% CI for different levels of TyG-BMI or TyG-WC at different specific ages (due to the limited number of deaths over the age of 80, the corresponding YLLs were calculated only for each 5 years of age in the 45–80 age group as a specific age cohort [Fig. 5 and Supplementary Table S11]).

For the overall interval from 45 to 80 years of age, the YLL with 95% CI for TyG-BMI Q1 and Q4 was 4.49 (2.90, 6.00) and 5.46 (3.47, 6.67) years, respectively, and for TyG-WC Q1 and Q4 was 3.24 (1.50, 5.11) and 5.31 (3.77, 6.66) years, respectively, when compared with Q2 and Q3. For a specific age, such as, at age 50 years, compared with quartiles 2 and 3, the YLL for Q1 and Q4 of TyG-BMI were 5.34 (3.65, 6.78) years and 6.66 (4.80, 7.99) years, respectively.

## Discussion

This study analysed middle-aged and older patients receiving primary care medical services throughout Poland – the largest Central and Eastern European country as well as the 6th largest European country. To our knowledge, this is the first comprehensive analysis of TyG-related indicators with total and premature all-cause mortality, and YLL in primary care. The main findings are: (i) TyG-BMI, TyG-WC, and TyG-WHtR were associated with total all-cause mortality in a U-shaped pattern, (ii) TyG-BMI, TyG-WC and TyG-WHtR were associated with premature all-cause mortality, and (iii) when compared to second/third quartile, TyG-BMI and TyG-WC, there were different degrees of YLL at different ages in first quartile and fourth quartile. These findings demonstrated the importance of focusing on surrogates of IR, TyG-related indicators, in reducing all-cause mortality, preventing premature deaths, and prolonging life expectancy in middle-aged and older primary care patients.

Elevated TG levels are recognised as an independent risk factor for cardiovascular risk due to their role in promoting an atherogenic dyslipidemia, which is characterised by an increase in remnant lipoproteins that contribute to endothelial dysfunction and inflammation.^34^ TG abnormalities are especially pronounced in the context of IR, as IR is strongly associated with abnormalities in lipid metabolism (high TG and remnant lipoproteins, low HDL-C, and large numbers of small, dense LDL particles). In the setting of IR, insulin no longer inhibits hormone-sensitive lipase in visceral adipose tissue and hence serum free fatty acid levels can rise and provide hepatocytes with more substrate for TG biosynthesis. These TG can then either be packaged into nascent very low-density lipoprotein particles and secreted into plasma, undergo B-oxidation, be converted into glucose via activation of phosphoenolpyruvate carboxykinase, or they may be deposited in the hepatic parenchyma yielding hepatic steatosis. Moreover, as hepatocytes become insulin resistant, there is increased gluconeogenesis and glucagon induced glycogenolysis, inducing a persistent hyperglycemic state.^35^ From the perspective of physiological mechanisms, the combined indicator of TyG combining TG and glucose reflects the metabolic changes of IR. Indeed, several previous IR diagnostic validation studies have evaluated the consistency between TyG and IR. Vasques et al. reported higher concordance of TyG with the gold standard hyperglycemic euglycaemic clamp test than with HOMA-IR (TyG: *r* = −0.64, *P* < 0.0001; HOMA-IR: *r* = −0.51, *P* < 0.0001) and better C-index of TyG in identifying IR than HOMA- IR (TyG: 0.79 [0.69–0.89]; HOMA-IR: 0.77 [0.66–0.88]).^36^ Also, Guerrero-Romero et al. showed that TyG was associated with the hyperglycemic clamp test with a correlation coefficient of −0.68 and that the C-index for the diagnosis of IR by TyG was 0.858, with high sensitivity (96.5%) and specificity (85.0%).^37^ Overall, TyG provides a comprehensive assessment of IR via derangements in lipid and glucose metabolism and thus has potential application in primary care to represent the IR status of patients.

IR is associated with enhanced inflammatory responses (via activation of nuclear factor-kappaB and receptors of advanced glycosylated end products), oxidative stress (mitochondrial dysfunction and activation of multiple oxidase enzymes), and the development or progression of several chronic metabolic diseases,^38^ which may be associated with an increased risk of all-cause mortality. Several studies have investigated the association between TyG and all-cause mortality. For example, Lopez-Jaramillo et al. reported that TyG was not significantly associated with the risk of cardiovascular and non-cardiovascular mortality in middle-income countries among participants aged 35–70 years.^24^ Similarly, our study also did not find a significant association between TyG with total and premature all-cause mortality. However, our analysis further revealed that TyG-BMI and TyG-WC were significantly associated with total and premature all-cause mortality, extending the perspective of assessing IR and mortality risk based solely on TyG, especially in middle-aged and older primary care populations. Sun et al. reported a U-shaped relationship between TyG and all-cause mortality in the general middle-aged and older population in the United States, the adjusted Cox proportion hazards model analysis showed a significant association between Q3 of TyG and all-cause mortality (Q1 as the reference group, Q3: aHR 0.84, 95% CI 0.73–0.98, *P* < 0.05), and the inflection point for the Cox proportion hazards model between TyG and all-cause mortality was 9.18.^39^ However, a J-shaped relationship between TyG and all-cause mortality was also observed in our European cohort, with a similar inflection point of 9.537 for the Cox proportional hazards model. Xu et al. reported that high levels of TyG in early life were significantly associated with all-cause mortality at long-term follow-up (median 25 years) in young Americans, and RCS analyses showed a linear trend between TyG and all-cause mortality.^40^ However, this differs from our results, possibly because of the large variation in the age range of the study cohort and the fact that changes in TyG over long-term follow-up may have a considerable impact on the analysis of results. Additionally, the results of a meta-analysis of ≥500,000 general populations from different cohorts showed no statistically significant association between the TyG and all-cause mortality (the highest levels of TyG were compared with the lowest levels of TyG, aHR 1.08, 95% CI 0.92–1.27, I^2^ = 87%).^41^ However, the four included studies showed high heterogeneity due to I^2^ over 75%, suggesting that the significant differences in the results of these studies may be caused by other factors such as differences in study design, interventions. Therefore, the relationship between TyG and all-cause mortality remains controversial and requires further study.

TyG adjusted by BMI, WC, and WHtR have been proven to have the potential for the diagnosis of IR and metabolic syndrome.^42^^,^^43^ Several studies have explored the association between obesity index adjusted TyG and mortality. For example, Kityo et al. found that the RCS analysis curves for TyG-BMI and TyG-WC with all-cause mortality were U-shaped or J-shaped,^44^ which is similar to our results. Dang et al. reported that TyG-WC (area under the curve was 0.63) showed better diagnostic performance for cardiovascular mortality than TyG (area under the curve was 0.61), and the correlation with various cardiometabolic diseases was also stronger than that of TyG.^45^ Furthermore, Zhan et al. found that TyG-BMI was significantly associated with all-cause mortality in patients undergoing peritoneal dialysis (aHR 1.19, 95% CI 1.09–1.31), and the second and third quartile groups had the lowest risk of all-cause mortality.^46^

Nevertheless, no prior study has explored the association between obesity index adjusted TyG and mortality in the middle-aged and older population. Adjusting the TyG for obesity indicators provides a more comprehensive assessment of IR by further incorporating body fat distribution based on glucose and lipid metabolism. The potential mechanisms underlying the U-shaped relationship between TyG-BMI or TyG-WC and all-cause mortality risk may be: (i) Low levels of TyG-BMI or TyG-WC may reflect malnutrition status where the body’s energy reserves are decreased and immune system function may be impaired, increasing the risk of various infections and diseases,^47^ thereby raising the risk of death, similar to the U-shaped relationship between BMI and all-cause mortality risk,^48^ (ii) high levels of TyG-BMI or TyG-WC are usually indicative of the presence of higher degrees of IR, metabolic disorders, and excess fat accumulation, which are associated with increased inflammation, oxidative stress, atherosclerotic disease, HTN, and endocrine dysregulation, leading to increased all-cause mortality.^49^ Besides, high levels of TyG-BMI or TyG-WC are accompanied by greater IR than low levels of TyG-BMI or TyG-WC, which may lead to a higher risk of death and more YLLs.

Although TyG-BMI and TyG-WC showed no significant interactions with age and sex on mortality risk, their associations varied across subgroups, indicating potential differences in the underlying mechanisms based on age and sex. Regarding age differences, in adults aged ≥ 65 years, sarcopenia and changes in body composition may affect fat distribution as assessed by BMI or WC,^50^^,^^51^ resulting in a weaker relationship between TyG-BMI or TyG-WC and mortality risk in this advanced age group. Furthermore, the associations between TyG-BMI and TyG-WC and premature all-cause mortality were stronger in females. This may be attributed to higher subcutaneous fat accumulation in females, along with hormonal fluctuations that increase the IR risk and metabolic disorders,^52^ making them more responsive to TyG-related indicators.

This study provides new insights into understanding the relationship between TyG and all-cause mortality and longevity in middle-aged and older adults, and lays the foundation for future research, especially by further exploring the relationship between TyG and other IR-related clinical outcomes, which will help to reveal the underlying mechanisms. In clinical practice, TyG-BMI and TyG-WC are promising as useful tools for assessing long-term all-cause mortality in middle-aged and older patients in primary care and allow healthcare professionals to identify high-risk populations for early/upfront intensive interventions.^53^^,^^54^ However, as ageing leads to increased fat and decreased muscle mass in older adults, the traditional BMI method may inaccurately reflect obesity,^50^^,^^51^ so some caution is needed when interpreting TyG-BMI results. Additionally, since BMI and WC may capture different health risk information, it may be necessary to further evaluate their joint effects on mortality risk in future prospective studies. For example, constructing a more comprehensive IR risk score by standardising and weighting TyG, BMI and WC, to better evaluate their joint effect on mortality risk.

Our study is only observational, although our results suggest that controlling for TyG-BMI or TyG-WC within certain limits may have survival benefits (based on the results of our threshold size analysis, the nadir of risk of all-cause mortality was 221.668 and 7.660 for TyG-BMI and TyG-WC, respectively), nevertheless, whether this will ultimately affect life expectancy or all-cause mortality for individuals or the global population’s life span remains unknown and cannot be extrapolated from these data, and therefore needs to be interpreted with caution. Future research should include large-scale, prospective, and interventional studies to validate these thresholds and explore their application in middle-aged and older adults to determine whether TyG-BMI and TyG-WC can be used as effective tools for early screening of high-risk all-cause mortality to improve public health and clinical primary care practice.

In addition, since TyG and TyG-related indicators may only represent a short-term IR state at baseline measurement, a single assessment of these indicators may not fully capture their cumulative metabolic burden over time and their long-term impact on health. A recent study has shown that distinct TyG trajectories are significantly associated with different mortality risks.^40^ Future large-scale prospective studies assess dynamic changes in TyG and TyG-related indicators over time, thereby clarifying how these fluctuations impact long-term all-cause mortality risk and life expectancy.

This study has several significant strengths. First, although previous studies have explored the association between TyG or TyG-related indicators and mortality risk, evidence within primary care settings remains scarce. The LIPIDOGRAM2015 was a nationwide cross-sectional study covering 16 major administrative regions and around 400 primary care clinics. This design ensures a diverse and representative sample, providing a broader perspective on the relationship between TyG or TyG-related indicators and mortality risk in middle-aged and older populations specifically within primary care. Second, our study is the first to include premature all-cause mortality and YLL, which have not been considered in previous research on TyG-related indicators. By examining these endpoints, our analysis goes beyond traditional mortality outcomes, providing a more nuanced understanding of the impact of TyG-related indicators on both lifespan and health quality. Third, a rigorous and scientific random sampling methodology and a rational questionnaire design enhanced the reliability of the study. Fourth, the study had a long-term follow-up of more than five years, providing sufficient time to observe all-cause mortality events. Together, these strengths ensure the scientific validity and applicability of the study results.

However, there are some limitations in this analysis. First, a clear causal relationship between TyG-related indicators and all-cause mortality may not be established and other potential residual confounders (such as information on renal function, CKD treatment) may be present. Second, although the TyG-related indicators have been shown to be a useful index for assessing IR, they may not fully represent the biomechanisms associated with IR. Third, the follow-up period in this study was about 5.7 years, which may not allow for evaluation of the association of TyG-related indicators with mortality outcomes of interest over longer follow-up periods. Fourth, TyG, BMI, and WC at baseline are prone to fluctuation through exercise and diet, and therefore, TyG and TyG-related indicators at baseline measurements may only represent a short-term metabolic state. Unfortunately, we did not evaluate the associations between altered TyG-related indicators and mortality outcomes of interest. Fifth, our YLLs were based on the complex statistical model in the R package “lillies”, which has been applied in several previous studies, but the accuracy of the results may be affected by the dataset used and the model chosen. Sixth, our study only included all-cause mortality and did not analyse the associations between TyG-related indicators with different NCDs and deaths due to specific NCDs. Finally, this study is based on a Polish primary care cohort, so external validation studies on the applicability of the TyG-related indicators and mortality management in other regions and other ethnicities are required.

TyG-BMI and TyG-WC, surrogates for IR, were strongly associated with total and premature all-cause mortality. Moreover, the low and high levels of TyG-BMI and TyG-WC adversely affected life expectancy. Fasting TG, fasting glucose, BMI and WC are all commonly measured indicators in primary care, implying the usefulness and immediate feasibility of using TyG-BMI and TyG-WC in primary care for long-term mortality risk stratification.

## Contributors

Jacek Jozwiak (JJ), Maciej Banach (MB), Marek Gierlotka (MG), and Tadeusz Osadnik (TO) contributed to the acquisition and interpretation of data. Yang Chen (YC), Ying Gue (YG), and Gregory Y.H. Lip (GYHL) initiated, planned, and designed the study. YC, Ziyi Zhong (ZZ), YG, and Garry McDowell (GM) conducted the literature review. YC and YG conducted the statistical analyses, as well as the preparation of figures and tables, with statistical validation performed by GM and GYHL. The first draft of the manuscript was written by YC and ZZ. YG, MB, GM, Dimitri P. Mikhailidis (DPM), Peter P. Toth (PPT), Peter E. Penson (PEP), Tomasz Tomasik (TT), Adam Windak (AW), MG, TO, Agnieszka Kuras (AK), Marcin Miga (MM), JJ, and GYHL revised the manuscript. YG, MB, JJ, and GYHL ensured management of the project and the study. All named authors meet the criteria for authorship as outlined by the International Committee of Medical Journal Editors, take collective responsibility for the integrity of the work, and have provided their approval for its publication.

## Data sharing statement

The dataset used for this study is not public available.

## Declaration of interests

Garry McDowell reports: receiving grants from Horizon Europe (grant number: 101136244); receiving contracts from Alder Hospital Children’s Chairty and Peel Medical Trust. Peter P. Toth reports: receiving consulting fees from Merck; payment or honoraria for lectures, presentations, speakers bureaus, manuscript writing or educational events were received from Amgen, Lilly, and Novo-Nordisk. Peter E. Penson reports: he owns four shares in Astra Zeneca PLC – full disclosure, no obvious link to this manuscript. Tomasz Tomasik reports: personal fees for lectures, presentations, speakers bureaus, manuscript writing or educational events were received from Boehringer Ing, Biofarm, AstraZeneca, Novartis, Novo Nordisk Pharma, and Teva. Marek Gierlotka reports: receiving consulting fees from Novartis, Sanofi, Amgen, and AstraZeneca; payment or honoraria for lectures, presentations, speakers bureaus, manuscript writing or educational events were received from Bayer, Novartis, Sanofi, Berlin Chemie, Novo Nordisk, Amgen, and Boehringer Ingelheim; receiving support for attending meetings and/or travel from Novartis and Servier; he is a member of Polish Cardiac Society. Tadeusz Osadnik reports: consulting fees were received from Novartis; payment or honoraria for lectures, presentations, speakers bureaus, manuscript writing or educational events were received from Amgen, Novartis, and Sanofi; he is an investigator in a study on Polygenic Risk Score, which has received grants from National Science Center, Poland; he is also a member of Polish Genetic Society. Other authors report no conflicts of interest.
