# Supplementary material for: Impact of surrogates for insulin resistance on mortality and life expectancy in primary care: a nationwide cross-sectional study with registry linkage (LIPIDOGRAM2015)
Source: Lancet Reg Health Eur. 2024 Dec 12;49:101182. doi: 10.1016/j.lanepe.2024.101182 (PMC11697418; doi:10.1016/j.lanepe.2024.101182)
Supplement: Supplementary Methods, Figs. S1–S22, and Tables S1–S11 [file mmc1.pdf]

STROBE Statement—Checklist of items that should be included in reports of ***cohort studies***

	Item No	Recommendation	Page
**Title and abstract**	1	(*a*) Indicate the study's design with a commonly used term in the title or the abstract	**1**
		(*b*) Provide in the abstract an informative and balanced summary of what was done and what was found	**3**
**Introduction**			
Background/rationale	2	Explain the scientific background and rationale for the investigation being reported	**4**
Objectives	3	State specific objectives, including any prespecified hypotheses	**4**
**Methods**			
Study design	4	Present key elements of study design early in the paper	**4**
Setting	5	Describe the setting, locations, and relevant dates, including periods of recruitment, exposure, follow-up, and data collection	**4-5**
Participants	6	(*a*) Give the eligibility criteria, and the sources and methods of selection of participants. Describe methods of follow-up	**4-5**
		(*b*) For matched studies, give matching criteria and number of exposed and unexposed	**NA**
Variables	7	Clearly define all outcomes, exposures, predictors, potential confounders, and effect modifiers. Give diagnostic criteria, if applicable	**4-5**
Data sources/ measurement	8*	For each variable of interest, give sources of data and details of methods of assessment (measurement). Describe comparability of assessment methods if there is more than one group	**4-5**
Bias	9	Describe any efforts to address potential sources of bias	**4-5**
Study size	10	Explain how the study size was arrived at	**4**
Quantitative variables	11	Explain how quantitative variables were handled in the analyses. If applicable, describe which groupings were chosen and why	**5-6**
Statistical methods	12	(*a*) Describe all statistical methods, including those used to control for confounding	**6-7**
		(*b*) Describe any methods used to examine subgroups and interactions	**4-7**
		(*c*) Explain how missing data were addressed	**NA**
		(*d*) If applicable, explain how loss to follow-up was addressed	**NA**
		(*e*) Describe any sensitivity analyses	**7**
**Results**			
Participants	13*	(a) Report numbers of individuals at each stage of study—eg numbers potentially eligible, examined for eligibility, confirmed eligible, included in the study, completing follow-up, and analysed	**7**
		(b) Give reasons for non-participation at each stage	**7**
		(c) Consider use of a flow diagram	**7**
Descriptive data	14*	(a) Give characteristics of study participants (eg demographic, clinical, social) and information on exposures and potential confounders	**7**
		(b) Indicate number of participants with missing data for each variable of interest	**NA**
		(c) Summarise follow-up time (eg, average and total amount)	**7**
Outcome data	15*	Report numbers of outcome events or summary measures over time	**8**
Main results	16	(*a*) Give unadjusted estimates and, if applicable, confounder-adjusted estimates and their precision (eg, 95% confidence interval). Make clear	**7-9**

		which confounders were adjusted for and why they were included	
		(*b*) Report category boundaries when continuous variables were categorized	**7-9**
		(*c*) If relevant, consider translating estimates of relative risk into absolute risk for a meaningful time period	**7-9**
Other analyses	17	Report other analyses done—eg analyses of subgroups and interactions, and sensitivity analyses	**9-10**
**Discussion**			
Key results	18	Summarise key results with reference to study objectives	**10**
Limitations	19	Discuss limitations of the study, taking into account sources of potential bias or imprecision. Discuss both direction and magnitude of any potential bias	**13-14**
Interpretation	20	Give a cautious overall interpretation of results considering objectives, limitations, multiplicity of analyses, results from similar studies, and other relevant evidence	**10-14**
Generalisability	21	Discuss the generalisability (external validity) of the study results	**10-14**
**Other information**			
Funding	22	Give the source of funding and the role of the funders for the present study and, if applicable, for the original study on which the present article is based	**3,7**

*Give information separately for exposed and unexposed groups.

Note: An Explanation and Elaboration article discusses each checklist item and gives methodological background and published examples of transparent reporting. The STROBE checklist is best used in conjunction with this article (freely available on the Web sites of PLoS Medicine at http://www.plosmedicine.org/, Annals of Internal Medicine at http://www.annals.org/, and Epidemiology at http://www.epidem.com/). Information on the STROBE Initiative is available at http://www.strobe-statement.org.

List of LIPIDOGRAM2015 Investigators

B Al-Shaer, W Andrusewicz, M Andrzejczuk-Rosa, E Anusz-Gaszewska, A Bagińska, P Balawajder, G Bańka, E Barańska-Skubisz, B Barbara Przyczyna, S Bartkowiak, J Bartodziej, M Bartosiewicz, M Basałyga, A Batyra, A Bąk, M Bednarz, K Bejnar, W Bernacki, M Betiuk-Kwiatkowska, S Biegaj, M Bień, W Bilski, M Biłogan, G Biruta-Pawłowska, A Biskup, B Błaszczyk, H Błaszczyk, T Błońska-Jankowska, B Bogacka-Gancarczyk, M Bojanowska, E Bonda, J Borowik-Skwarek, J Borowska, J Bruckner, J Brzostek, M Brzuchacz, M Budzyńska, I Bulzacka-Fugiel, J Bulzak, K Bunikowski, A Cebulska, T Celka, E Cempel-Nowak, W Chechliński, A Chludzińska, D Chmiel, M Chmielewska, M Cichy, A Ciemięga, A Ciepluch, I Cieszyńska, B Czajka, B Czapla, M Czerner, B Czerwińska, W Czuryszkiewicz, E Daleka, Z Dawid, M Dąbrowska, R Dąbrowska, D Dąbrowski, M Dąbrowski, K Demczyszyn, A Dębowska-Serwińska, J Dmochowski, J Dobrzecka-Kiwior, E Dolanowska, H Dolanowski, P Dołek, M Domagała, H Domański, A Doszel, D Duda, M Dudkowska, B Dudziuk, P Dybciak, M Dymanowski, L Dziadzio-Bolek, M Eicke, H El-Hassan, A Eremus, M Fąferek-Muller, E Figura-Roguska, I Fijałkowska-Kaczmarek, M Flis, T Florczak, M Florczuk, E Foryszewska-Witan, W Frydrych, A Fugiel, E Futyma, A Gaca-Jaroszewicz, I Gajdamowicz, K Ganczarski, A Gatnar, M Gers, A Głowacki, K Głód, J Godula, J Gołąb, M Gołębiewski, E Goszczyńska, K Gościcka, A Górna-Hajduga, E Górny, T Grabowska, R Grabowski, A Graczyk-Duda, A Gromow, A Grudewicz, J Gruszecka, A Gruszka, J Gryboś, J Grzebyk, A Grzechowiak, D Grzesiak, T Grześkowiak, A Guźla, G Hachuła, B Hawel, H Hiltawska, E Honkowicz, J Ignatowicz, K Imielski, A Iwaniura, A Jagieła-Szymala, M Jalć-Sowała, A Janczylik, E Janisz, M Janiszek, K Jankiewicz-Ziobro, K Januszewska, A Jaremek, A Jaros-Urbaniak, J Jarosz, P Jarosz, W Jasiński, M Jezierska-Wasilewska, T Jędraszewski, A Jędrzejowska, R Józefowicz, K Juźwin, E Kacprzak, J Kaczmarek-Szewczyk, M Kaczmarzyk, R Kandziora, C Kaniewski, L Karolak-Brandt, S Kasperczyk, E Kasperek-Dyląg, I Kedziora, A Kępa, J Kiciński, J Kielak-Al-Hosam, Ł Kiełczawa, P Kilimowicz, K Kitliński, T Kiwka, U Klein, L Klichowicz, A Klimowicz, B Klonowski, B Kmolek, E Kobyłko-Klepacka, A Kocoń, A Kolenda, E Kollek, M Kopeć, B Koper-Kozikowska, J Koralewska, M Korczyńska, M T Korzeniewski, A Kosk, K Kotarski, E Kowalczyk, M Kowalczyk, I Kowalik, B Kozak-Błażkiewicz, M Kozik, D Kozłowska, E Kozłowska, M Kozłowska, T Kozubski, K Kózka, L Kraśnik, T Krężel, B Krochmal, B Król, G Król, J Król, T Królikowska, H Kruszewska, B Krygier-Potrykus, W Krystek, J Krzysztoń, T Kubicki, A Kuczmierczyk-El-Hassan, W Kuczyńska-Witek, D Kujda, A Kurowski, I Kurzelewska-Solarz, M Kwaczyńska, M Kwaśniak, P Kwaśniak, T Kwietniewska, A Łebek-Ordon, A Lebiedowicz, L Lejkowska-Olszewska, M Lentas, A Lesiewicz-Ksycińska, M Limanowski, S Łoniewski, J A Łopata, B Łubianka, I Łukasiuk, M Łużna, M Łysiak, B Łysik, Z Machowski, J Maciaczyk-Kubiak, G Mackiewicz-Zabochnicka, Z Magner-Krężel, S Majda, P Malinowski, J Mantyka, E Marchlik, G Martyna-Ordyniec, J Marzec, M Marzec, R Matejko-Wałkiewicz, M Mazur, M Michalczak, A Michalska-Żyłka, M Michniewicz, D Mika-Staniszewska, E Mikiciuk, T Mikołajczak, J Milewski, E Miller, B Misiaszek, M Mizik-Łukowska, E Młyńczyk-Pokutycka, M Mocek, M Moczała, M Morawska-Hermanowicz, P Moryc, A Moskal, S Moskal, A Moździerz, P Moździerz, M Mrozińska, K Mrozowicz, G Mróz, T Munia, A Mura, M Muras-Skudlarska, E Z Murawska, Ł Murawski, R Murawski, R Musielak, K Nadaj, W Nagarnowicz, R Napierała, M Niedźwiecka, A Niemirski, J Nikiel, M Nosal, W Nowacki, J Nowak, M Nyrka, A Obst, J Ochowicz, E Ogonowska, M Oleszczyk, A Ołdakowski, I Ołowniuk-Stefaniak, J Ordowska-Rejman, M Orliński, B Osińska, A Ostańska-Burian, A Paciorkowska, U Paczkowska, L Paluch, L Pałka, J Paszko-Wojtkowska, A Paszkowska, E Pawlak-Ganczarska, W Pawlik, I Pawłowska, M Paździora, G Permiakow, A Petlic-Marendziak, T Piasecka, E Piaścińska, A Piktel, A Pilarska-Igielska, A Piotrkowska, K Piwowar-Klag, M Planer, J Plewa, P Płatkiewicz, B Płonczyńska, A Podgórska, M Polewska, B Porębska, P Porwoł, I Potakowska, A Prokop, J Przybylski, M Przybyła, H Psiuk, K Ptak, G Puzoń, N Rabiza, S Rachwalik, E Raczyńska, M Raniszewska, A Romanek-Kozik, A Rosa, K Rosa, A Rozewicz, J Rudzka-Kałwak, J Rusak, D Rutkowska, M Rybacki, D Rybińska, A Rycyk-Sadowska, L Rynda, B Rynkiewicz, B Sadowska-Krawczyk, M Sadowska-Zarzycka, B Sarnecka, E Sawalach-Tomanik, B Sidor-Drozd, M Siemieniak-Dębska, A Sieroń, B Siewniak-Zalewska, A Sikora, B Sitarska-Pawlina, J Skorupski, I Skrzypińska-Mansfeld, J Skubisz, R Skwarek, M Słodyczka, M Smentek, K Smolińska, B Solarz, W Sosnowska, B Sroka, H Stachura, D Stangreciak, M Staniak, Z Stańczyk, D Stańszczak-Ozga, E Startek, M Stefańczyk, R Stelmach, E Sternadel-Rączka, M Sternik, J Stępień, J Stocka, M Stokowska-Wojda, M Studler-Karpińska, W Suchorukow, W Sufryd, B Supłacz, J Sygacz, Ł Szczepański, J Szkandera, J Szłapa-Zellner, D Szydlarska, T Śliwa, J Śliwka, Ł Śmiejkowski, A Targońska, E Tesarska, M Tobiasz, J Tomaka, K Tomalska-Bywalec, E Tomiak, S Topczewski, A Trawińska, L Trela-Mucha, D Trojanowski, M Trzaskowska, B Trzcińska-Larska, A Trznadel-Mozul, K Ulanicka-Liwoch, M Urbanowicz, A Uthke-Kluzek, J Waczyński, J Walczak, L Warsz, M Wasyńczuk, U Wąchała-Jędras, D Wąsowicz, J Wczysła, F Wenda, E Werner-Kubicka, E Weryszko, B Węgrzynowska, M Wiaksa, M

Wiankowski, A Wicherek, R Wieczorek, R Wiencek, G Wienzek-Tatara, B Wierzbicka, M Wierzbicki, B Wilczyńska, D Wilmańska, P Winiarski, A Wiszniewska-Pabiszczak, M B Witkowska, J Witzling, A Wlaź, I Wojtkowiak, J Woydyło, K Woźniak, A Wójtowicz, J Wrona, M Wrońska, H Wujkowska, J Wyrąbek, O Wysokiński, R Zakrzewski, J Zaleska-Zatkalik, J Zaleski, M Zalewska-Dybciak, E Zalewska, B Zalewska-Uchimiak, J Zawadzka-Krajewska, J Zawadzki, A Zieliński, E Zubrycka, I Żybort, M Żymełka

Supplementary Methods. Recursive algorithm for inflection point calculation and threshold effect analysis

Once a non-linear relationship between TyG-related indicators and total all-cause mortality was detected using restricted cubic splines, and examining whether the TyG-related indicators were partitioned into intervals. We applied segmented regression (also known as piece-wise regression) that is using a separate line segment to fit each interval. Log-likelihood ratio test comparing one-line (non-segmented) model to segmented regression model was used to determine whether threshold exists. The inflection point that connecting the segments was based on the model gives maximum likelihood, and it was determined using a two-step recursive algorithm as follows.

Step 1

We began by narrowing the potential inflection point to a 10 percentile range of the TyG-related indicator. This was done by testing 19 segmented regression models with candidate inflection points set at every 5th percentile between the 5th and 95th percentiles of the TyG-related indicators. Each model was fitted to identify which percentile provided the maximum likelihood. The precise inflection point was narrowed down to ± 4% percentile of the percentile points which gives the maximum likelihood among the 19 segmented regression models, which we labeled as Kmin (tp2.min in R code) and Kmax (tp2.max in R code), respectively.

Step 2

In the step 2, we applied a recursive method to refine the exact inflection point between Kmin and Kmax. The specific method is to first run three models with inflection points set at the 25th (Q1), 50th (Q2), and 75th (Q3) percentiles within the Kmin-Kmax range to find out which quartile point gives the model with highest likelihood among these three models. Then we narrow down the Kmin and Kmax to the range of ± 25% of the corresponding quartile point. This process was repeated recursively, reducing the range by 50% at each iteration, until we identified a specific value of the independent variable that served as the final inflection point. Using this final inflection point would give the segmented regression model highest likelihood. Then, Cox proportional hazards model for the threshold effect analysis on either side of this inflection point.

The R code used in this analysis was as follows:

```
cuttab<-function(fit,var,wdtmp,cutpoint=NULL) {
  UseMethod("cuttab")
}

############
gettpval.cph<-function(fit,var,wdtmp, tppmin=NA, tppmax=NA,dec=NA) {
  if (missing(dec)) dec<-3
  wdtmp<-as.data.frame(wdtmp)
  xTMP <- wdtmp[,var]
  tmp.ss<-seq(0.05,0.95,0.05)
  tp<-quantile(xTMP,probs=tmp.ss,na.rm=TRUE)
  tmp.llk<-rep(NA,length(tmp.ss))
  call<-fit[["call"]]
  ff<- paste0(call)
  fml<-ff[2]
```

```
fml<-paste(fml,"+tmp.X")
if (!is.na(tppmin) & !is.na(tppmax)) {
  tp2.min = tppmin; tp2.max = tppmax;
} else {
  for (k in (1:length(tmp.ss))) {
    tmp.X<-(xTMP > tp[k])*(xTMP-tp[k]); wdtmp1<-cbind(wdtmp,tmp.X)
    tmp.mdl<- coxph(formula(fml),weights=wdtmp$weights,data=wdtmp,na.action=na.omit,method="breslow")
    tmp.llk[k]<-tmp.mdl$loglik[2];
    rm(wdtmp1, tmp.X)
  }
  tp1<-tmp.ss[which.max(tmp.llk)]
  tp2.min = tp1 - 0.04
  tp2.max = tp1 + 0.04
  if (tp2.min<0.05) {tp2.min=0.05}
  if (tp2.max>0.95) {tp2.max=0.95}
}
tp.pctlrange<-quantile(xTMP,probs=c(tp2.min,tp2.max),na.rm=TRUE)
tp.range<-unique(xTMP[xTMP>tp.pctlrange[1] & xTMP<tp.pctlrange[2]])
while (length(tp.range)>5) {
  tmp.pct3<-quantile(tp.range,probs=c(0,0.25,0.5,0.75,1),type=3)
  tmp.llk3<-rep(NA,3)
  for (k in (2:4)) {
    tmp.X<-(xTMP>tmp.pct3[k])*(xTMP-tmp.pct3[k]); wdtmp1<-cbind(wdtmp,tmp.X)
    tmp.mdl<- coxph(formula(fml),data=wdtmp,na.action=na.omit)

    tmp.llk3[k-1]<-tmp.mdl$loglik[2];
    rm(wdtmp1, tmp.X)
  }
  tmp.min3<-which.max(tmp.llk3)
  tp.range<-tp.range[tp.range>=tmp.pct3[tmp.min3] & tp.range<=tmp.pct3[tmp.min3+2]]
}
if (length(tp.range)>0) {
  if (length(tp.range)==1) {tp.val=tp.range[1];} else {
    tmp.llk<-rep(NA,length(tp.range))
    for (k in (1:length(tp.range))) {
      tmp.X<-(xTMP>tp.range[k])*(xTMP-tp.range[k]); wdtmp1<-cbind(wdtmp,tmp.X)
      tmp.mdl<- coxph(formula(fml),data=wdtmp,na.action=na.omit)

      tmp.llk[k]<-tmp.mdl$loglik[2];
      rm(wdtmp1, tmp.X)
```

```
      }
      tp.val<-tp.range[which.max(tmp.llk)]
    }
  } else { tp.val<-tp.pctlrange[1];}
  return(round(tp.val,dec));
}

############
cuttab.cph<-function(fit,var,wdtmp,cutpoint=NULL) {
  cut.p<-gettpval(fit=fit,var =var,wdtmp=wdtmp)
  if (!is.null(cutpoint)) {cut.p<-cutpoint}
  dt<-as.data.frame(wdtmp)
  x <-wdtmp[,var]
  X1 <-(x<=cut.p)*(x-cut.p)
  X2 <-(x> cut.p)*(x-cut.p)
  dt <-cbind(dt,X1,X2)
  call<-fit[["call"]]
  ff<- paste0(call)
  fml<-ff[2];fml1<-paste(fml,"+X2");
  fml2<-transformfml(fit,var)
  fml2<-paste(fml2,"+X1+X2");
  fit0<-coxph(formula(fml),weights=wdtmp$weights,data=wdtmp)
  fit1<-coxph(formula(fml1),weights=wdtmp$weights,data=wdtmp)
  fit2<-coxph(formula(fml2),weights=wdtmp$weights,data=wdtmp)
  t1<-coef(summary(fit0));t1.1<-summary(fit0)$conf.int
  t1.2<-round(coef(summary(fit0))[var,2],3);t1.3<-round(coef(summary(fit0))[var,5],3);
  t1.4<-round(summary(fit0)$conf.int[var,3],3);t1.5<-round(summary(fit0)$conf.int[var,4],3)
  t1<-paste0(t1.2,"(",t1.4,"-",t1.5,")",t1.3)
  t2<-coef(summary(fit1));t2.1<-summary(fit1)$conf.int;
  t2.2<-round(coef(summary(fit1))[var,2],3);t2.3<-round(coef(summary(fit1))[var,5],3)
  t2.4<-round(summary(fit1)$conf.int[var,3],3);t2.5<-round(summary(fit1)$conf.int[var,4],3)
  t2<-paste0(t2.2,"(",t2.4,"-",t2.5,")",t2.3)
  t3<-coef(summary(fit2));t3.1<-summary(fit2)$conf.int;
  t3.2<-round(t3[paste0("X2"),2],3);t3.3<-round(t3[paste0("X2"),5],3);
  t3.4<-round(t3.1[paste0("X2"),3],3);t3.5<-round(t3.1[paste0("X2"),4],3)
  t3<-paste0(t3.2,"(",t3.4,"-",t3.5,")",t3.3)
  i1<-paste0("<",cut.p)
  i2<-paste0(">",cut.p)
  plrt<-pvformat(1-pchisq(2*(logLik(fit2)[1]-logLik(fit0)[1]),1),3)
  d1<-c("Model 1 Fitting model by standard linear regression",t1)
```

```
  d2<-c("Model 2 Fitting model by two-piecewise linear regression","")
  d3<-c("Inflection point",cut.p)
  d4<-c(i1,t2)
  d5<-c(i2,t3)
  d6<-c("P for likelihood ratio test",plrt)
  dat<-rbind(d1,d2,d3,d4,d5,d6)
  colnames(dat)<-c("Outcome","the effect size, 95%CI, P value")
  dat
}

#############
transformfml <- function(fit,var){
  UseMethod("transformfml")
}

transformfml.cph<-function(fit,var) {
  fml.1<-as.character(fit[["call"]][["formula"]][[2]])
  fml.2<-paste0(fml.1[1],"(",fml.1[2],",",fml.1[3],")")
  name<-fit[["Design"]][["name"]]
  name[name== var] <- NA
  name<- na.omit(name)
  name1<-paste0(name,collapse = "+")
  name2<-paste0("~",name1)
  f<-paste0(fml.2,name2)
  f
}

###############
pvformat<-function(p,dec) {
  pp <- sprintf(paste("%.",dec,"f",sep=""),as.numeric(p))
  if (is.matrix(p)) {pp<-matrix(pp, nrow=nrow(p)); colnames(pp)<-colnames(p);rownames(pp)<-rownames(p);}
  lw <- paste("<",substr("0.00000000000",1,dec+1),"1",sep="");
  pp[as.numeric(p)<(1/10^dec)]<-lw
  return(pp)
}
```

Supplementary Table S1. The interquartile ranges for the TyG-related indicators in cohort for different outcomes.

		Total all-cause mortality & Years of life lost	Premature all-cause mortality
TyG	Q1	TyG ≤ 8.4307	TyG ≤ 8.4342
	Q2	8.4307 < TyG ≤ 8.7674	8.4342 < TyG ≤ 8.7741
	Q3	8.7674 < TyG ≤ 9.1561	8.7741 < TyG ≤ 9.1643
	Q4	TyG > 9.1561	TyG > 9.1643
TyG-BMI	Q1	TyG ≤ 219.4245	TyG ≤ 219.7839
	Q2	219.4245 < TyG ≤ 251.3857	219.7839 < TyG ≤ 251.6716
	Q3	251.3857 < TyG ≤ 286.6459	251.6716 < TyG ≤ 287.4559
	Q4	TyG > 286.6459	TyG > 287.4559
TyG-WC	Q1	TyG ≤ 7.5144	TyG ≤ 7.4866
	Q2	7.5144 < TyG ≤ 8.4739	7.4866 < TyG ≤ 8.4605
	Q3	8.4739 < TyG ≤ 9.4340	8.4605 < TyG ≤ 9.4375
	Q4	TyG > 9.4340	TyG > 9.4375
TyG-WHtR	Q1	TyG ≤ 4.5541	TyG ≤ 4.5315
	Q2	4.5541 < TyG ≤ 5.0932	4.5315 < TyG ≤ 5.0708
	Q3	5.0932 < TyG ≤ 5.6807	5.0708 < TyG ≤ 5.6645
	Q4	TyG > 5.6807	TyG > 5.6645
TyG-WHR	Q1	TyG ≤ 7.2458	TyG ≤ 7.2332
	Q2	7.2458 < TyG ≤ 7.9352	7.2332 < TyG ≤ 7.9358
	Q3	7.9352 < TyG ≤ 8.6557	7.9358 < TyG ≤ 8.6715
	Q4	TyG > 8.6557	TyG > 8.6715

Abbreviations: BMI, body mass index; TyG, triglyceride-glucose index; WC, waist circumference; WHtR, waist-to-height ratio; WHR, waist-to-hip ratio.

Supplementary Table S2. Baseline characteristics of TyG quartile groups in the whole cohort.

Characteristic	Q1	Q2	Q3	Q4	*P-value*
Age, years	59.92 (53.29, 67.15)	61.65 (55.00, 68.15)	62.04 (55.91, 67.85)	61.16 (55.39, 67.44)	< 0.0001
Female, n (%)	1859 (69.6)	1778 (66.6)	1720 (64.3)	1432 (53.6)	< 0.0001
Higher Education, n (%)	1575 (58.9)	1446 (54.1)	1378 (51.6)	1222 (45.7)	< 0.0001
Urban Residence, n (%)	1353 (50.6)	1427 (53.4)	1367 (51.1)	1355 (50.7)	0.1348
Smoking status, n (%)					< 0.0001
Never	2308 (86.4)	2258 (84.5)	2239 (83.8)	2147 (80.4)	
Past/current	364 (13.6)	413 (15.5)	434 (16.2)	525 (19.6)	
Alcohol Consumption, n (%)					0.0098
Never	993 (37.2)	1018 (38.1)	985 (36.8)	906 (33.9)	
Moderate/high	1679 (62.8)	1653 (61.9)	1688 (63.2)	1766 (66.1)	
Regular Physical Activity, n (%)	1171 (43.8)	1047 (39.2)	1023 (38.3)	948 (35.5)	< 0.0001
Use of antiatherogenic Diet, n (%)	1884 (70.5)	1889 (70.7)	1855 (69.4)	1784 (66.8)	0.0060
Comorbidity, n (%)					
Diabetes Mellitus	162 (6.1)	251 (9.4)	460 (17.2)	866 (32.4)	< 0.0001
Hypertension	1227 (45.9)	1508 (56.5)	1669 (62.4)	1832 (68.6)	< 0.0001
Chronic Kidney Disease	63 (2.4)	72 (2.7)	90 (3.4)	113 (4.2)	< 0.0001
Myocardial Infarction	118 (4.4)	137 (5.1)	168 (6.3)	184 (6.9)	< 0.0001
Stroke	54 (2.0)	74 (2.8)	68 (2.5)	90 (3.4)	0.0224
Atrial Fibrillation	146 (5.5)	162 (6.1)	194 (7.3)	185 (6.9)	0.0312
Pharmacotherapy, n (%)					
Treatment of Dyslipidaemia	795 (29.8)	916 (34.3)	1033 (38.6)	1093 (40.9)	< 0.0001
Treatment of Diabetes Mellitus	152 (5.7)	234 (8.8)	425 (15.9)	824 (30.8)	< 0.0001
Treatment of Hypertension	1179 (44.1)	1447 (54.2)	1612 (60.3)	1767 (66.1)	< 0.0001
Antiplatelet Treatment	332 (12.4)	379 (14.2)	416 (15.6)	425 (15.9)	0.0011
Anticoagulant Treatment	186 (7.0)	202 (7.6)	241 (9.0)	216 (8.1)	0.0393
Laboratory results					
LDL-C, mg/dL	119.00 (95.00, 145.00)	131.00 (102.00, 159.00)	131.00 (102.00, 162.00)	131.00 (101.00, 164.00)	< 0.0001
HDL-C, mg/dL	62.45 (52.80, 73.20)	55.60 (47.20, 64.70)	50.40 (43.10, 58.50)	44.20 (38.20, 52.40)	< 0.0001
TC, mg/dL	194.20 (168.10, 220.10)	201.60 (171.10, 231.10)	201.10 (171.25, 234.00)	212.15 (180.70, 246.90)	< 0.0001

Abbreviations: HDL-C, high-density lipoprotein cholesterol; LDL-C, low-density lipoprotein cholesterol; TC, total cholesterol; TyG, triglyceride-glucose index.

Supplementary Table S3. Baseline characteristics of triglyceride-glucose index adjusted body mass index quartile groups in the whole cohort.

Characteristic	Q1	Q2	Q3	Q4	*P-value*
Age, years	59.40 (53.03, 66.97)	61.26 (54.56, 67.71)	62.17 (56.24, 68.21)	61.85 (55.89, 67.64)	< 0.0001
Female, n (%)	1972 (73.8)	1643 (61.5)	1572 (58.8)	1602 (60.0)	< 0.0001
Higher Education, n (%)	1683 (63.0)	1504 (56.3)	1286 (48.1)	1148 (43.0)	< 0.0001
Urban Residence, n (%)	1477 (55.3)	1422 (53.2)	1371 (51.3)	1232 (46.1)	< 0.0001
Smoking status, n (%)					< 0.0001
Never	2124 (79.5)	2252 (84.3)	2264 (84.7)	2312 (86.5)	
Past/current	548 (20.5)	420 (15.7)	408 (15.3)	360 (13.5)	
Alcohol Consumption, n (%)					0.4659
Never	979 (36.6)	964 (36.1)	953 (35.7)	1006 (37.6)	
Moderate/high	1693 (63.4)	1708 (63.9)	1719 (64.3)	1666 (62.4)	
Regular Physical Activity, n (%)	1183 (44.3)	1096 (41.0)	1029 (38.5)	881 (33.0)	< 0.0001
Use of Antiatherogenic Diet, n (%)	1840 (68.9)	1855 (69.4)	1854 (69.4)	1863 (69.7)	0.9227
Comorbidity, n (%)					
Diabetes Mellitus	150 (5.6)	271 (10.1)	472 (17.7)	846 (31.7)	< 0.0001
Hypertension	1038 (38.8)	1427 (53.4)	1742 (65.2)	2029 (75.9)	< 0.0001
Chronic Kidney Disease	54 (2.0)	80 (3.0)	93 (3.5)	111 (4.2)	< 0.0001
Myocardial Infarction	112 (4.2)	128 (4.8)	181 (6.8)	186 (7.0)	< 0.0001
Stroke	52 (1.9)	69 (2.6)	76 (2.8)	89 (3.3)	0.0166
Atrial Fibrillation	138 (5.2)	149 (5.6)	179 (6.7)	221 (8.3)	< 0.0001
Use of Medications, n (%)					
Treatment of Dyslipidaemia	722 (27.0)	920 (34.4)	1059 (39.6)	1136 (42.5)	< 0.0001
Treatment of Diabetes Mellitus	137 (5.1)	258 (9.7)	434 (16.2)	806 (30.2)	< 0.0001
Treatment of Hypertension	989 (37.0)	1371 (51.3)	1680 (62.9)	1965 (73.5)	< 0.0001
Antiplatelet Treatment	308 (11.5)	343 (12.8)	408 (15.3)	493 (18.5)	< 0.0001
Anticoagulant Treatment	148 (5.5)	191 (7.1)	218 (8.2)	288 (10.8)	< 0.0001
Laboratory results					
LDL-C, mg/dL	128.00 (102.00, 154.00)	130.00 (102.00, 162.00)	126.00 (99.00, 157.00)	126.00 (96.00, 156.00)	< 0.0001
HDL-C, mg/dL	63.00 (53.10, 73.60)	53.95 (45.70, 64.10)	49.80 (42.10, 58.30)	46.05 (39.20, 54.43)	< 0.0001
TC, mg/dL	205.65 (179.40, 234.20)	203.20 (173.08, 235.20)	198.90 (169.87, 229.00)	199.10 (167.68, 231.50)	< 0.0001

Abbreviations: BMI, body mass index; HDL-C, high-density lipoprotein cholesterol; LDL-C, low-density lipoprotein cholesterol; TC, total cholesterol.

Supplementary Table S4. Baseline characteristics of triglyceride-glucose index adjusted waist circumference quartile groupsin the whole cohort.

Characteristic	Q1	Q2	Q3	Q4	*P-value*
Age, years	58.12 (52.13, 65.18)	61.48 (55.34, 67.85)	62.52 (56.27, 68.74)	62.49 (56.55, 68.14)	< 0.0001
Female, n (%)	2300 (86.1)	1823 (68.2)	1474 (55.2)	1192 (44.6)	< 0.0001
Higher Education, n (%)	1779 (66.6)	1465 (54.8)	1251 (46.8)	1126 (42.1)	< 0.0001
Urban Residence, n (%)	1499 (56.1)	1395 (52.2)	1354 (50.7)	1254 (46.9)	< 0.0001
Smoking status, n (%)					0.0035
Never	2191 (82.0)	2226 (83.3)	2288 (85.6)	2247 (84.1)	
Past/current	481 (18.0)	446 (16.7)	384 (14.4)	425 (15.9)	
Alcohol Consumption, n (%)					< 0.0001
Never	1052 (39.4)	990 (37.1)	958 (35.9)	902 (33.8)	
Moderate/high	1620 (60.6)	1682 (62.9)	1714 (64.1)	1770 (66.2)	
Regular Physical Activity, n (%)	1227 (45.9)	1081 (40.5)	1001 (37.5)	880 (32.9)	< 0.0001
Use of Antiatherogenic Diet, n (%)	1826 (68.3)	1875 (70.2)	1871 (70.0)	1840 (68.9)	0.3911
Comorbidity, n (%)					
Diabetes Mellitus	107 (4.0)	277 (10.4)	449 (16.8)	906 (33.9)	< 0.0001
Hypertension	1017 (38.1)	1445 (54.1)	1749 (65.5)	2025 (75.8)	< 0.0001
Chronic Kidney Disease	60 (2.2)	68 (2.5)	92 (3.4)	118 (4.4)	< 0.0001
Myocardial Infarction	72 (2.7)	121 (4.5)	170 (6.4)	244 (9.1)	< 0.0001
Stroke	45 (1.7)	56 (2.1)	98 (3.7)	87 (3.3)	< 0.0001
Atrial Fibrillation	111 (4.2)	152 (5.7)	175 (6.5)	249 (9.3)	< 0.0001
Use of Medications, n (%)					
Treatment of Dyslipidaemia	672 (25.1)	966 (36.2)	1053 (39.4)	1146 (42.9)	< 0.0001
Treatment of Diabetes Mellitus	97 (3.6)	260 (9.7)	419 (15.7)	859 (32.1)	< 0.0001
Treatment of Hypertension	968 (36.2)	1387 (51.9)	1680 (62.9)	1970 (73.7)	< 0.0001
Antiplatelet Treatment	269 (10.1)	354 (13.2)	408 (15.3)	521 (19.5)	< 0.0001
Anticoagulant Treatment	136 (5.1)	191 (7.1)	202 (7.6)	316 (11.8)	< 0.0001
Laboratory results					
LDL-C, mg/dL	129.00 (103.00, 156.00)	130.00 (102.00, 161.00)	128.00 (99.00, 158.00)	123.00 (95.00, 155.00)	< 0.0001
HDL-C, mg/dL	63.95 (54.70, 74.52)	54.70 (47.07, 64.10)	49.40 (42.80, 58.10)	44.00 (37.98, 52.10)	< 0.0001
TC, mg/dL	207.00 (181.10, 235.10)	203.00 (173.68, 235.10)	199.50 (169.08, 228.10)	195.90 (165.60, 230.15)	< 0.0001

Abbreviations: HDL-C, high-density lipoprotein cholesterol; LDL-C, low-density lipoprotein cholesterol; TC, total cholesterol.

Supplementary Table S5. Baseline characteristics of triglyceride-glucose index adjusted waist-to-height ratio quartile groups in the whole cohort.

Characteristic	Q1	Q2	Q3	Q4	*P-value*
Age, years	57.62 (51.76, 64.63)	61.13 (54.93, 67.52)	62.56 (56.46, 68.64)	63.34 (57.28, 69.05)	< 0.0001
Female, n (%)	2006 (75.0)	1599 (59.9)	1596 (59.7)	1588 (59.4)	< 0.0001
Higher Education, n (%)	1766 (66.1)	1514 (56.7)	1267 (47.4)	1074 (40.2)	< 0.0001
Urban Residence, n (%)	1498 (56.0)	1411 (52.8)	1340 (50.1)	1253 (46.9)	< 0.0001
Smoking status, n (%)					0.0053
Never	2189 (81.9)	2225 (83.3)	2263 (84.7)	2275 (85.1)	
Past/current	484 (18.1)	446 (16.7)	409 (15.3)	397 (14.9)	
Alcohol Consumption, n (%)					< 0.0001
Never	975 (36.5)	910 (34.1)	958 (35.9)	1059 (39.6)	
Moderate/high	1698 (63.5)	1761 (65.9)	1714 (64.1)	1613 (60.4)	
Regular Physical Activity, n (%)	1251 (46.8)	1079 (40.4)	1018 (38.1)	841 (31.5)	< 0.0001
Use of Antiatherogenic Diet, n (%)	1835 (68.6)	1878 (70.3)	1877 (70.2)	1822 (68.2)	0.2137
Comorbidity, n (%)					
Diabetes Mellitus	101 (3.8)	264 (9.9)	448 (16.8)	926 (34.7)	< 0.0001
Hypertension	973 (36.4)	1422 (53.2)	1754 (65.6)	2087 (78.1)	< 0.0001
Chronic Kidney Disease	60 (2.2)	68 (2.5)	84 (3.1)	126 (4.7)	< 0.0001
Myocardial Infarction	78 (2.9)	140 (5.2)	177 (6.6)	212 (7.9)	< 0.0001
Stroke	44 (1.6)	64 (2.4)	95 (3.6)	83 (3.1)	< 0.0001
Atrial Fibrillation	111 (4.2)	155 (5.8)	174 (6.5)	247 (9.2)	< 0.0001
Use of Medications, n (%)					
Treatment of Dyslipidaemia	667 (25.0)	892 (33.4)	1101 (41.2)	1177 (44.0)	< 0.0001
Treatment of Diabetes Mellitus	94 (3.5)	246 (9.2)	417 (15.6)	878 (32.9)	< 0.0001
Treatment of Hypertension	924 (34.6)	1353 (50.7)	1700 (63.6)	2028 (75.9)	< 0.0001
Antiplatelet Treatment	260 (9.7)	351 (13.1)	394 (14.7)	547 (20.5)	< 0.0001
Anticoagulant Treatment	132 (4.9)	203 (7.6)	206 (7.7)	304 (11.4)	< 0.0001
Laboratory results					
LDL-C, mg/dL	129.00 (103.00, 155.00)	130.00 (101.00, 161.00)	128.50 (99.00, 158.00)	123.00 (95.00, 155.00)	< 0.0001
HDL-C, mg/dL	63.30 (53.60, 74.00)	53.90 (45.80, 63.80)	50.10 (42.80, 59.00)	45.40 (38.70, 53.70)	< 0.0001
TC, mg/dL	206.50 (180.20, 234.20)	201.60 (172.70, 233.20)	200.85 (169.40, 231.85)	196.75 (166.35, 231.10)	< 0.0001

Abbreviations: HDL-C, high-density lipoprotein cholesterol; LDL-C, low-density lipoprotein cholesterol; TC, total cholesterol.

Supplementary Table S6. Baseline characteristics of triglyceride-glucose index adjusted waist-to-hip ratio quartile groups in the whole cohort.

Characteristic	Q1	Q2	Q3	Q4	*P-value*
Age, years	58.84 (52.61, 66.22)	61.76 (55.22, 68.01)	62.45 (56.47, 68.44)	61.42 (55.71, 67.71)	< 0.0001
Female, n (%)	2463 (92.2)	2032 (76.1)	1487 (55.6)	807 (30.2)	< 0.0001
Higher Education, n (%)	1767 (66.1)	1481 (55.5)	1232 (46.1)	1141 (42.7)	< 0.0001
Urban Residence, n (%)	1494 (55.9)	1367 (51.2)	1349 (50.4)	1292 (48.4)	< 0.0001
Smoking status, n (%)					< 0.0001
Never	2293 (85.8)	2259 (84.6)	2221 (83.1)	2179 (81.5)	
Past/current	379 (14.2)	411 (15.4)	453 (16.9)	493 (18.5)	
Alcohol Consumption, n (%)					< 0.0001
Never	1140 (42.7)	1074 (40.2)	918 (34.3)	770 (28.8)	
Moderate/high	1532 (57.3)	1596 (59.8)	1756 (65.7)	1902 (71.2)	
Regular Physical Activity, n (%)	1188 (44.5)	1018 (38.1)	1015 (38.0)	968 (36.2)	< 0.0001
Use of Antiatherogenic Diet, n (%)	1833 (68.6)	1894 (70.9)	1844 (69.0)	1841 (68.9)	0.2296
Comorbidity, n (%)					
Diabetes Mellitus	128 (4.8)	294 (11.0)	490 (18.3)	827 (31.0)	< 0.0001
Hypertension	1134 (42.4)	1503 (56.3)	1737 (65.0)	1862 (69.7)	< 0.0001
Chronic Kidney Disease	59 (2.2)	87 (3.3)	84 (3.1)	108 (4.0)	0.0020
Myocardial Infarction	59 (2.2)	99 (3.7)	182 (6.8)	267 (10.0)	< 0.0001
Stroke	39 (1.5)	69 (2.6)	82 (3.1)	96 (3.6)	< 0.0001
Atrial Fibrillation	114 (4.3)	159 (6.0)	174 (6.5)	240 (9.0)	< 0.0001
Use of Medications, n (%)					
Treatment of Dyslipidaemia	718 (26.9)	933 (34.9)	1078 (40.3)	1108 (41.5)	< 0.0001
Treatment of Diabetes Mellitus	118 (4.4)	272 (10.2)	467 (17.5)	778 (29.1)	< 0.0001
Treatment of Hypertension	1085 (40.6)	1448 (54.2)	1671 (62.5)	1801 (67.4)	< 0.0001
Antiplatelet Treatment	273 (10.2)	378 (14.2)	409 (15.3)	492 (18.4)	< 0.0001
Anticoagulant Treatment	162 (6.1)	172 (6.4)	222 (8.3)	289 (10.8)	< 0.0001
Laboratory results					
LDL-C, mg/dL	126.00 (100.00, 153.00)	131.00 (101.25, 161.00)	130.00 (101.00, 161.00)	124.00 (96.00, 155.00)	< 0.0001
HDL-C, mg/dL	63.50 (54.60, 74.20)	55.40 (47.73, 64.57)	49.60 (43.00, 58.30)	43.20 (37.50, 51.20)	< 0.0001
TC, mg/dL	203.80 (178.25, 232.40)	202.65 (173.03, 233.70)	200.30 (169.42, 232.50)	199.40 (168.40, 231.63)	< 0.0001

Abbreviations: HDL-C, high-density lipoprotein cholesterol; LDL-C, low-density lipoprotein cholesterol; TC, total cholesterol.

Supplementary Table S7. Threshold effect analysis of TyG-related biomarkers on total all-cause mortality.

	Adjusted HR (95% CI)	*P-value*
TyG		
Fitting by the standard linear model	1.077 (0.932, 1.245)	0.3169
Fitting by the two-piecewise linear model		
Inflection point: 9.537		
TyG < 9.537	0.883 (0.733, 1.063)	0.1889
TyG ≥ 9.537	1.847 (1.330, 2.566)	0.0003
P for log-likelihood ratio		0.0013
TyG-BMI		
Fitting by the standard linear model	1.001 (1.000, 1.003)	0.1473
Fitting by the two-piecewise linear model		
Inflection point: 221.668		
TyG-BMI < 221.668	0.984 (0.979, 0.989)	< 0.0001
TyG-BMI ≥ 221.668	1.004 (1.002, 1.006)	< 0.0001
P for log-likelihood ratio		< 0.0001
TyG-WC		
Fitting by the standard linear model	1.068 (1.008, 1.133)	0.0268
Fitting by the two-piecewise linear model		
Inflection point: 7.660		
TyG-WC < 7.660	0.632 (0.529, 0.754)	< 0.0001
TyG-WC ≥ 7.660	1.170 (1.097, 1.247)	< 0.0001
P for log-likelihood ratio		< 0.0001
TyG-WHtR		
Fitting by the standard linear model	1.103 (1.002, 1.214)	0.0457
Fitting by the two-piecewise linear model		
Inflection point: 4.639		
TyG-WHtR < 4.639	0.481 (0.356, 0.651)	< 0.0001
TyG-WHtR ≥ 4.639	1.280 (1.150, 1.426)	< 0.0001
P for log-likelihood ratio		< 0.0001
TyG-WHR		
Fitting by the standard linear model	1.154 (1.061, 1.256)	0.0009
Fitting by the two-piecewise linear model		
Inflection point: 9.114		
TyG-WHR < 9.114	0.998 (0.896, 1.112)	0.9758
TyG-WHR ≥ 9.114	1.577 (1.341, 1.855)	< 0.0001
P for log-likelihood ratio		< 0.0001

The Cox proportional hazards model was adjusted for age, sex, level of education, place of residence, smoking status, alcohol consumption, regular physical activity, use of antiatherogenic diet, diabetes mellitus, hypertension, chronic kidney disease, myocardial infarction, stroke, atrial fibrillation, low-density lipoprotein cholesterol, and high-density lipoprotein cholesterol.

Abbreviations: BMI, body mass index; CI, confidence interval; HR, hazard ratio; TyG, triglyceride-glucose index; WC, waist circumference; WHtR, waist-to-height ratio; WHR, waist-to-hip ratio.

Supplementary Table S8. Reclassification statistics comparing the performance of TyG-BMI and TyG-WC with BMI and WC for predicting all-cause mortality.

	Continuous NRI (95% CI), %	*P*	IDI (95% CI), %	*P*
TyG-BMI vs. BMI				
Basic model + BMI	*Reference*		*Reference*	
Basic model + TyG-BMI	11.25 (3.93-18.57)	0.0026	0.22 (0.05-0.39)	0.0118
TyG-WC vs. WC				
Basic model + WC	*Reference*		*Reference*	
Basic model + TyG-WC	10.43 (3.12-17.75)	0.0052	0.20 (0.07-0.34)	0.0034

Basic model included age, sex, level of education, place of residence, smoking status, alcohol consumption, regular physical activity, use of antiatherogenic diet, diabetes mellitus, hypertension, chronic kidney disease, myocardial infarction, stroke, atrial fibrillation, low-density lipoprotein cholesterol and high-density lipoprotein cholesterol.

Supplementary Table S9 Sensitivity analyses of TyG-related indicators and all-cause mortality and premature all-cause mortality adding use of treatments.

		Total all-cause mortality		Premature all-cause mortality	
		**aHR, 95% CI**	**P**	**aHR, 95% CI**	**P**
TyG-BMI	Q1	1.34 (1.08-1.66)	0.0088	1.46 (1.08-1.98)	0.0144
	Q2	*Reference*		*Reference*	
	Q3	1.02 (0.82-1.27)	0.8544	1.21 (0.90-1.61)	0.2035
	Q4	1.28 (1.03-1.59)	0.0252	1.49 (1.12-1.99)	0.0060
TyG-WC	Q1	1.45 (1.14-1.83)	0.0022	1.70 (1.22-2.36)	0.0016
	Q2	*Reference*		*Reference*	
	Q3	1.12 (0.90-1.38)	0.3227	1.23 (0.91-1.66)	0.1760
	Q4	1.30 (1.05-1.60)	0.0176	1.53 (1.14-2.06)	0.0048

The Cox proportional hazards model was adjusted for age, sex, level of education, place of residence, smoking status, alcohol consumption, regular physical activity, use of antiatherogenic diet, diabetes mellitus, hypertension, chronic kidney disease, myocardial infarction, stroke, atrial fibrillation, low-density lipoprotein cholesterol, and high-density lipoprotein cholesterol, treatment of dyslipidaemia, treatment of diabetes mellitus, treatment of hypertension, antiplatelet treatment, anticoagulant treatment. Abbreviations: BMI, body mass index; CI, confidence interval; HR, hazard ratio; TyG, triglyceride-glucose index; WC, waist circumference.

Supplementary Table S10 Mediation analysis of comorbidities in the associations between TyG-BMI/TyG-WC and all-cause mortality.

Exposures	Mediators	Mediated Proportion	
		Estimate (95% CI)	*P**
TyG-BMI	Diabetes Milletus	-0.30 (-2.87, 2.45)	0.820
	Hypertension	-0.64 (-2.63, 4.38)	0.702
	MI	2.14 (-6.30, 9.32)	0.460
	Stroke	-3.85 (-7.87, 7.54)	0.690
	AF	0.56 (-6.57, 10.20)	0.440
	CKD	0.05 (-5.91, 6.64)	0.770
TyG-WC	Diabetes Milletus	-1.22 e^{-3} (-3.42e^{-3}, 0.02)	0.316
	Hypertension	0.03 (-0.02, 0.18)	0.192
	MI	-3.57e^{-3} (-0.05, 0.11)	0.504
	Stroke	0.02 (-0.04, 0.07)	0.594
	AF	0.03 (-0.03, 0.14)	0.228
	CKD	1.40e^{-3} (-0.01, 0.05)	0.354

*The "mediation" package can only output *P* values to 3 decimal places; therefore, 4-decimal *P* values are not reported here.

The mediation analysis was adjusted for including age, sex, level of education, place of residence, smoking status, and alcohol consumption, regular physical activity, use of antiatherogenic diet, low-density lipoprotein cholesterol, and high-density lipoprotein cholesterol.

Supplementary Table S11 Excess years of life lost in first or fourth quartiles compared to second and third quartiles of TyG-BMI and TyG-WC.

	Excess years of life lost (95% confidence interval), years			
	TyG-BMI		TyG-WC	
Specific age	First quartile	Fourth quartile	First quartile	Fourth quartile
45 years	5.33 (3.69, 6.88)	6.83 (4.96, 8.21)	3.84 (2.15, 5.67)	6.68 (5.00, 8.15)
50 years	5.34 (3.65, 6.78)	6.66 (4.80, 7.99)	3.85 (2.04, 5.66)	6.70 (5.15, 8.19)
55 years	5.11 (3.32, 6.61)	6.42 (4.37, 7.60)	3.71 (1.76, 5.50)	6.53 (5.08, 8.02)
60 years	4.80 (3.10, 6.34)	5.76 (3.98, 7.07)	3.33 (1.48, 5.09)	5.83 (4.28, 7.19)
65 years	3.87 (2.20, 5.37)	4.94 (3.08, 6.28)	2.80 (0.85, 4.74)	4.95 (3.36, 6.35)
70 years	3.21 (1.56, 4.80)	4.25 (2.36, 5.54)	2.07 (0.32, 3.86)	3.85 (2.25, 5.23)
75 years	2.77 (1.02, 4.43)	3.56 (1.31, 4.93)	1.41 (-0.52, 3.38)	2.96 (1.18, 4.45)
80 years	1.90 (0.17, 3.38)	2.87 (0.49, 4.35)	0.72 (-1.25, 2.63)	2.17 (0.43, 3.71)
During 45 to 80 years	4.49 (2.90, 6.00)	5.46 (3.47, 6.67)	3.24 (1.50, 5.11)	5.31 (3.77, 6.66)

Abbreviations: BMI, body mass index; TyG, triglyceride-glucose index; WC, waist circumference.

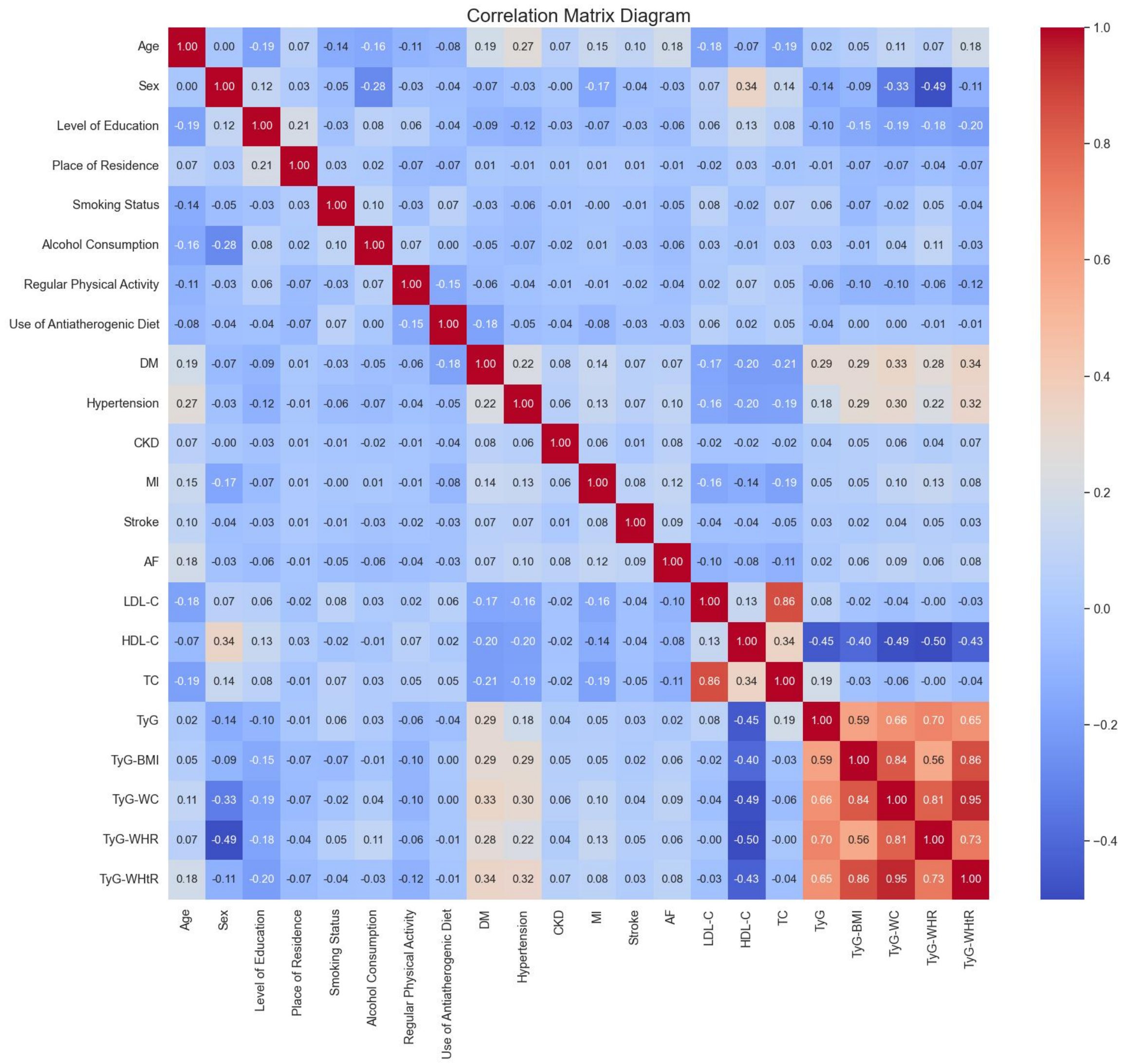

Supplementary Figure S1. Pearson correlation matrix between variables. The figure displays the Pearson correlation coefficients between the variables in this study. The colours and values indicate the strength of the correlation, with positive and negative correlations shown in different colours. Abbreviations: AF, atrial fibrillation; BMI, body mass index; CKD, chronic kidney disease; DM, diabetes mellitus; HDL-C, high-density lipoprotein cholesterol; LDL-C, low-density lipoprotein cholesterol; MI, myocardial infarction; TC, total cholesterol; TyG, triglyceride-glucose index; WC, waist circumference; WHtR, waist-to-height ratio; WHR, waist-to-hip ratio.

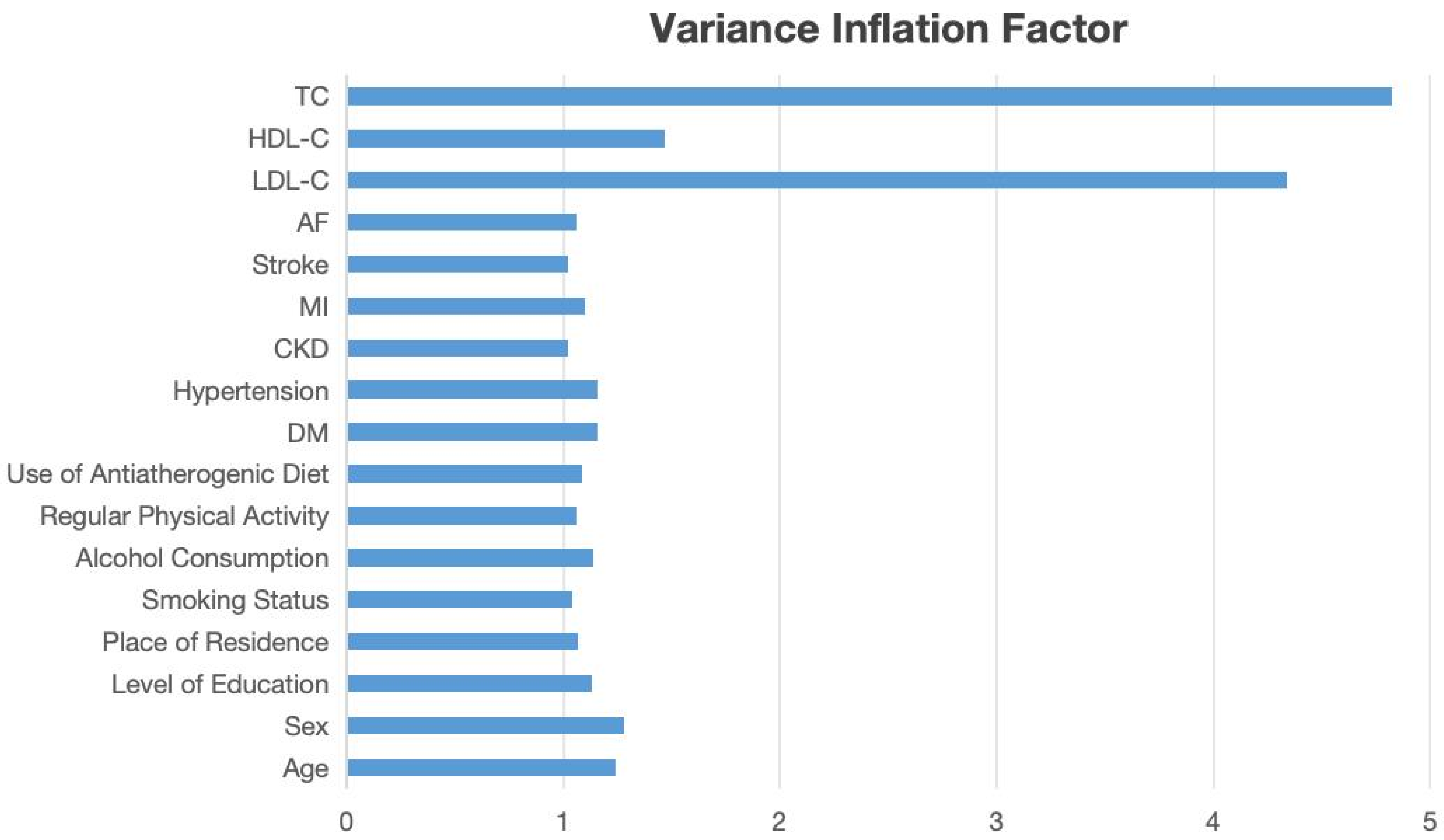

Supplementary Figure S2. Variance inflation factor analysis of variables. Abbreviations: AF, atrial fibrillation; BMI, body mass index; CKD, chronic kidney disease; DM, diabetes mellitus; HDL-C, high-density lipoprotein cholesterol; LDL-C, low-density lipoprotein cholesterol; MI, myocardial infarction; TC, total cholesterol.

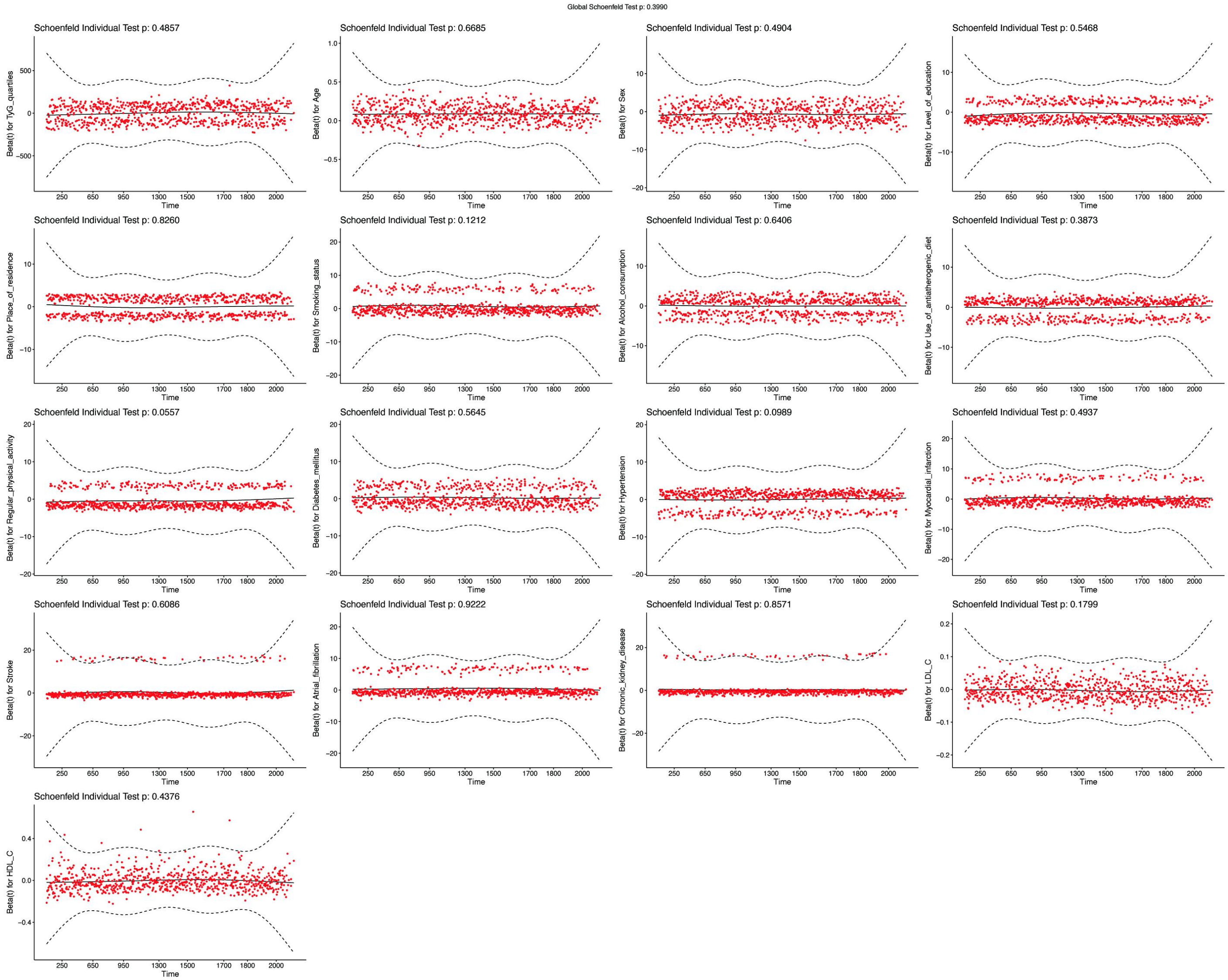

Supplementary Figure S3. Schoenfeld residuals plot for proportional hazards assumption in Cox models (TyG and total all-cause mortality). HDL_C, high-density lipoprotein cholesterol; LDL_C, low-density lipoprotein cholesterol; TyG, triglyceride-glucose index.

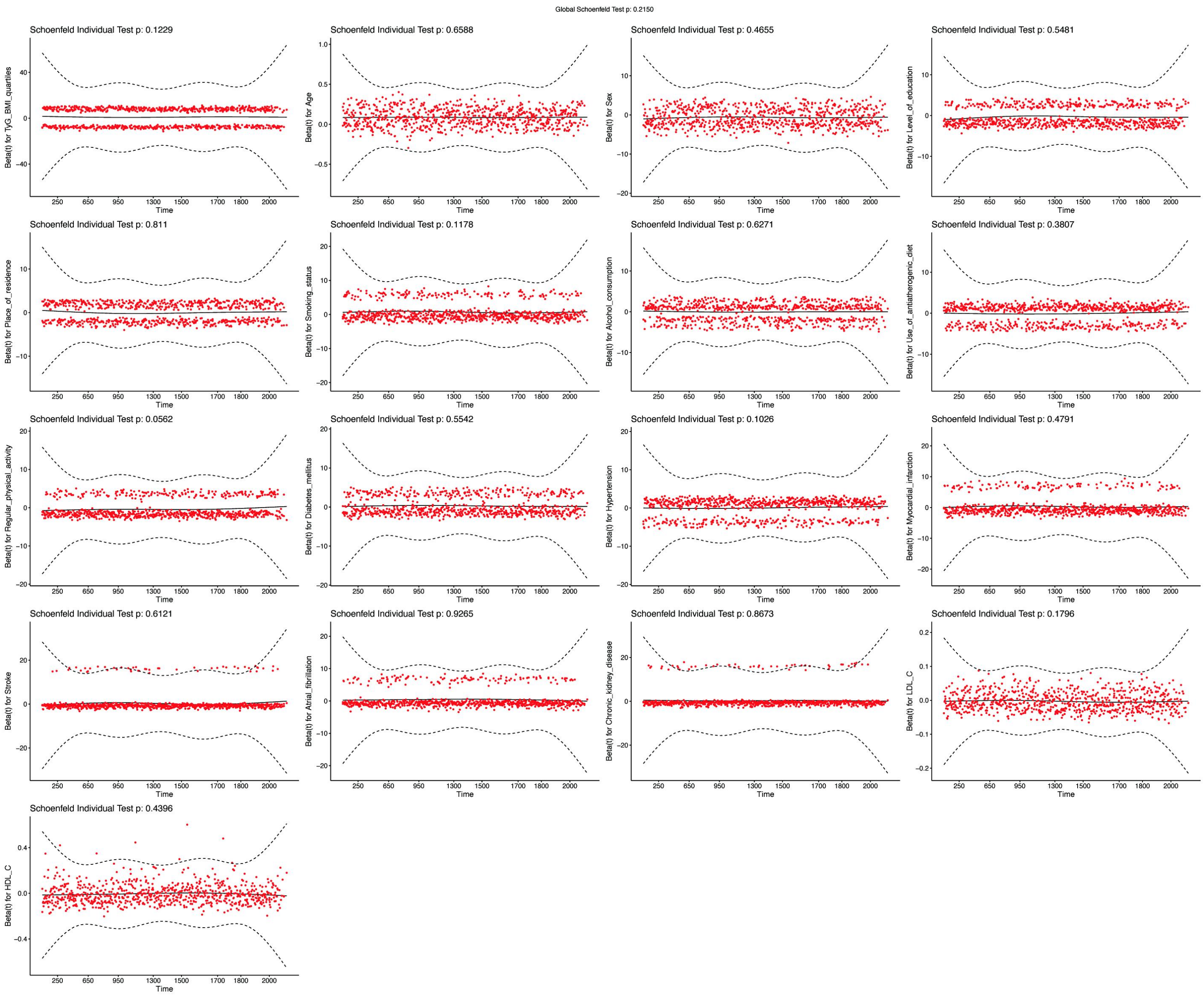

Supplementary Figure S4. Schoenfeld residuals plot for proportional hazards assumption in Cox models (TyG-BMI and total all-cause mortality). BMI, body mass index; HDL_C, high-density lipoprotein cholesterol; LDL_C, low-density lipoprotein cholesterol; TyG, triglyceride-glucose index.

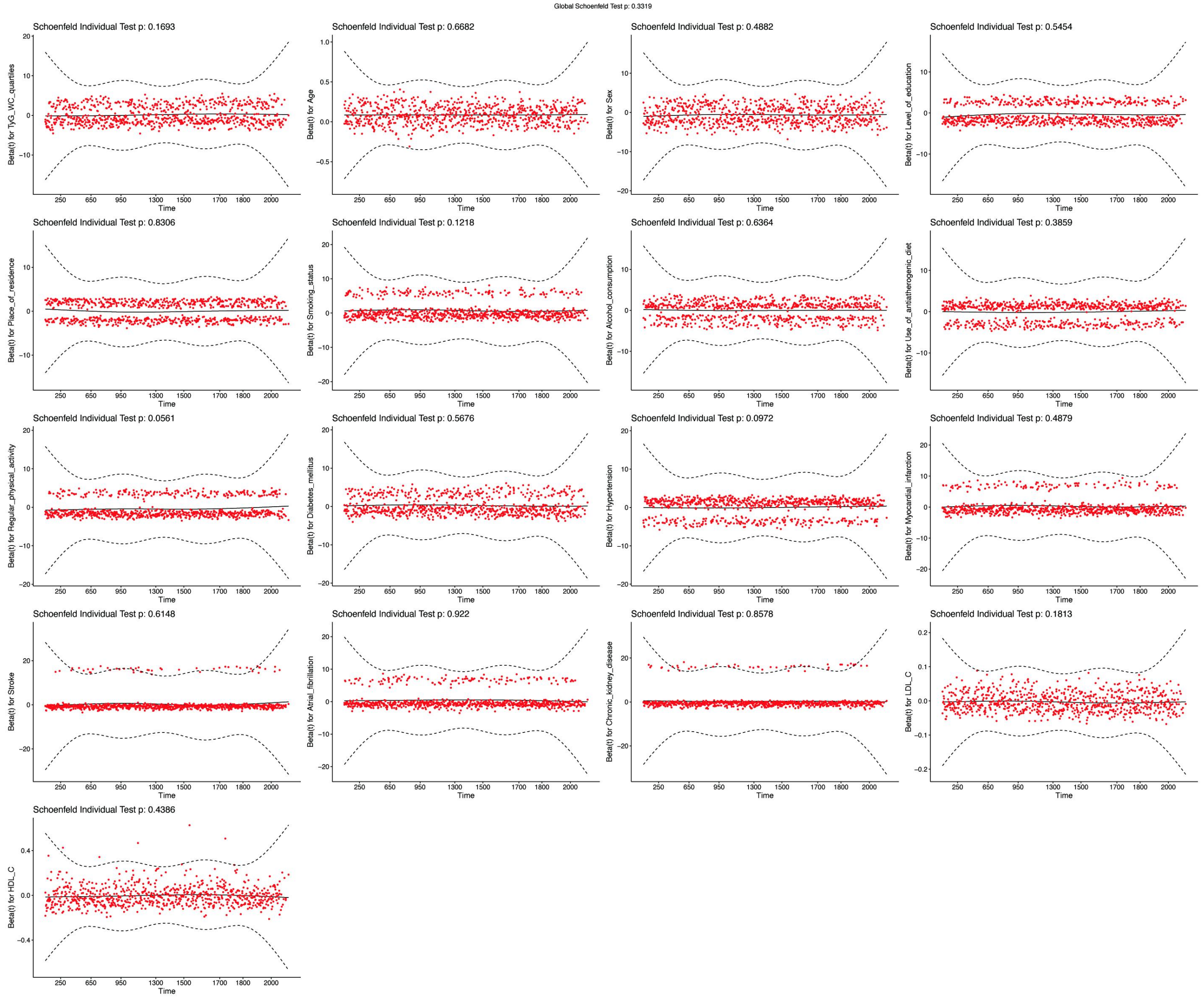

Supplementary Figure S5. Schoenfeld residuals plot for proportional hazards assumption in Cox models (TyG-WC and total all-cause mortality). HDL_C, high-density lipoprotein cholesterol; LDL_C, low-density lipoprotein cholesterol; TyG, triglyceride-glucose index; WC, waist circumference.

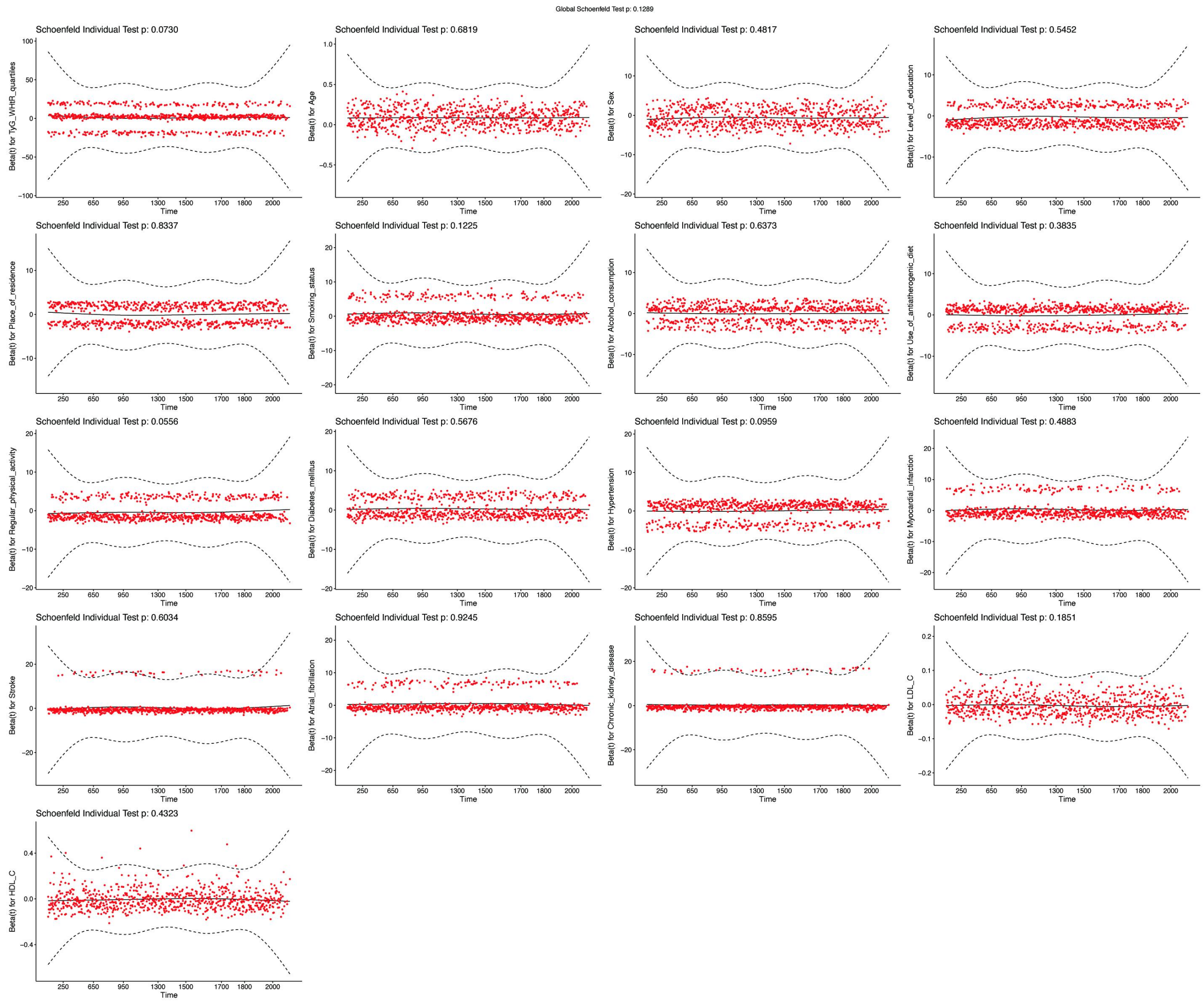

Supplementary Figure S6. Schoenfeld residuals plot for proportional hazards assumption in Cox models (TyG-WHtR and total all-cause mortality).
HDL_C, high-density lipoprotein cholesterol; LDL_C, low-density lipoprotein cholesterol; TyG, triglyceride-glucose index; WHtR, waist-to-height ratio.

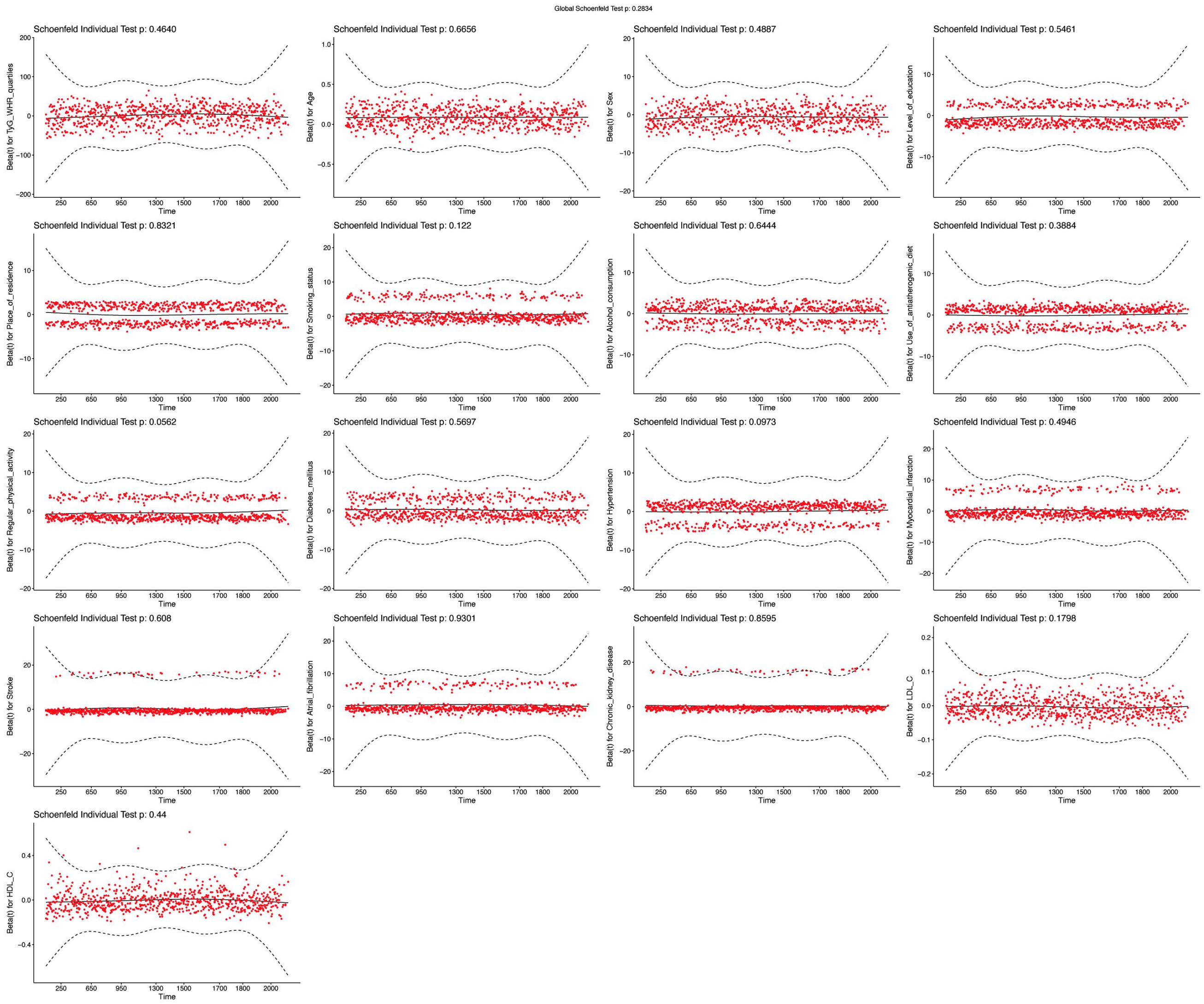

Supplementary Figure S7. Schoenfeld residuals plot for proportional hazards assumption in Cox models (TyG-WHR and total all-cause mortality). HDL_C, high-density lipoprotein cholesterol; LDL_C, low-density lipoprotein cholesterol; TyG, triglyceride-glucose index; WHR, waist-to-hip ratio.

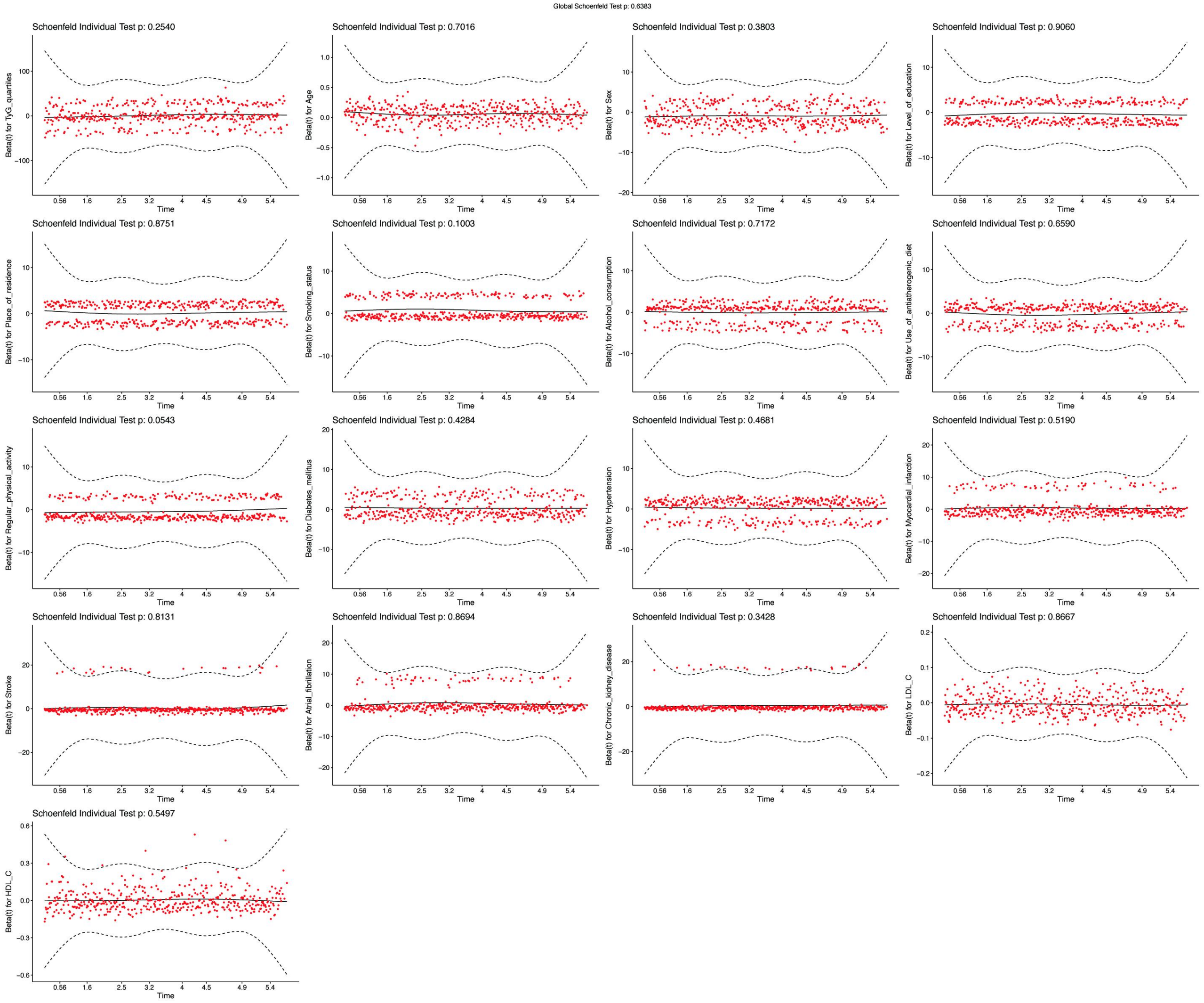

Supplementary Figure S8. Schoenfeld residuals plot for proportional hazards assumption in Cox models (TyG and premature all-cause mortality). HDL_C, high-density lipoprotein cholesterol; LDL_C, low-density lipoprotein cholesterol; TyG, triglyceride-glucose index.

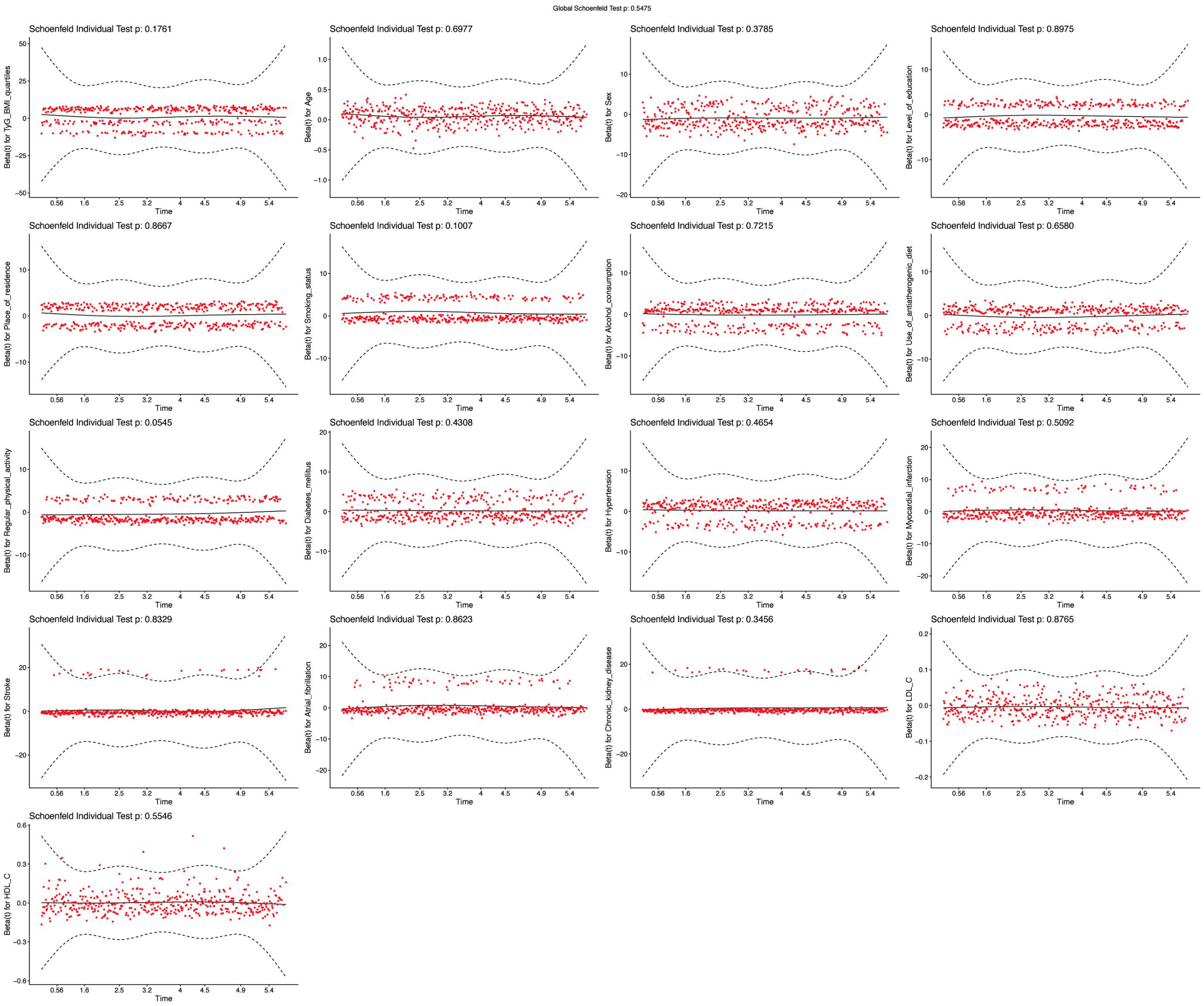

Supplementary Figure S9. Schoenfeld residuals plot for proportional hazards assumption in Cox models (TyG-BMI and premature all-cause mortality). BMI, body mass index; HDL_C, high-density lipoprotein cholesterol; LDL_C, low-density lipoprotein cholesterol; TyG, triglyceride-glucose index.

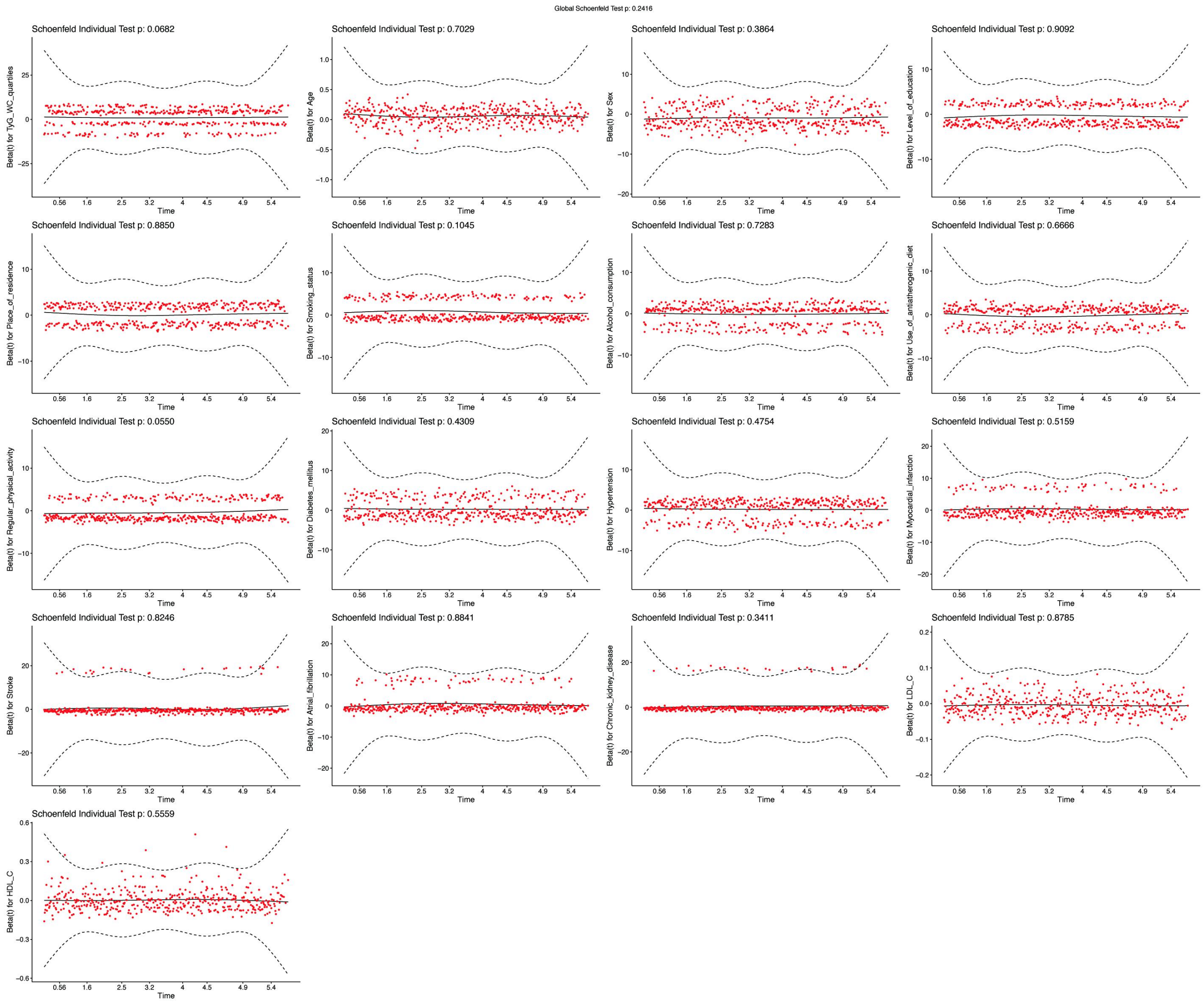

Supplementary Figure S10. Schoenfeld residuals plot for proportional hazards assumption in Cox models (TyG-WC and premature all-cause mortality). HDL_C, high-density lipoprotein cholesterol; LDL_C, low-density lipoprotein cholesterol; TyG, triglyceride-glucose index; WC, waist circumference.

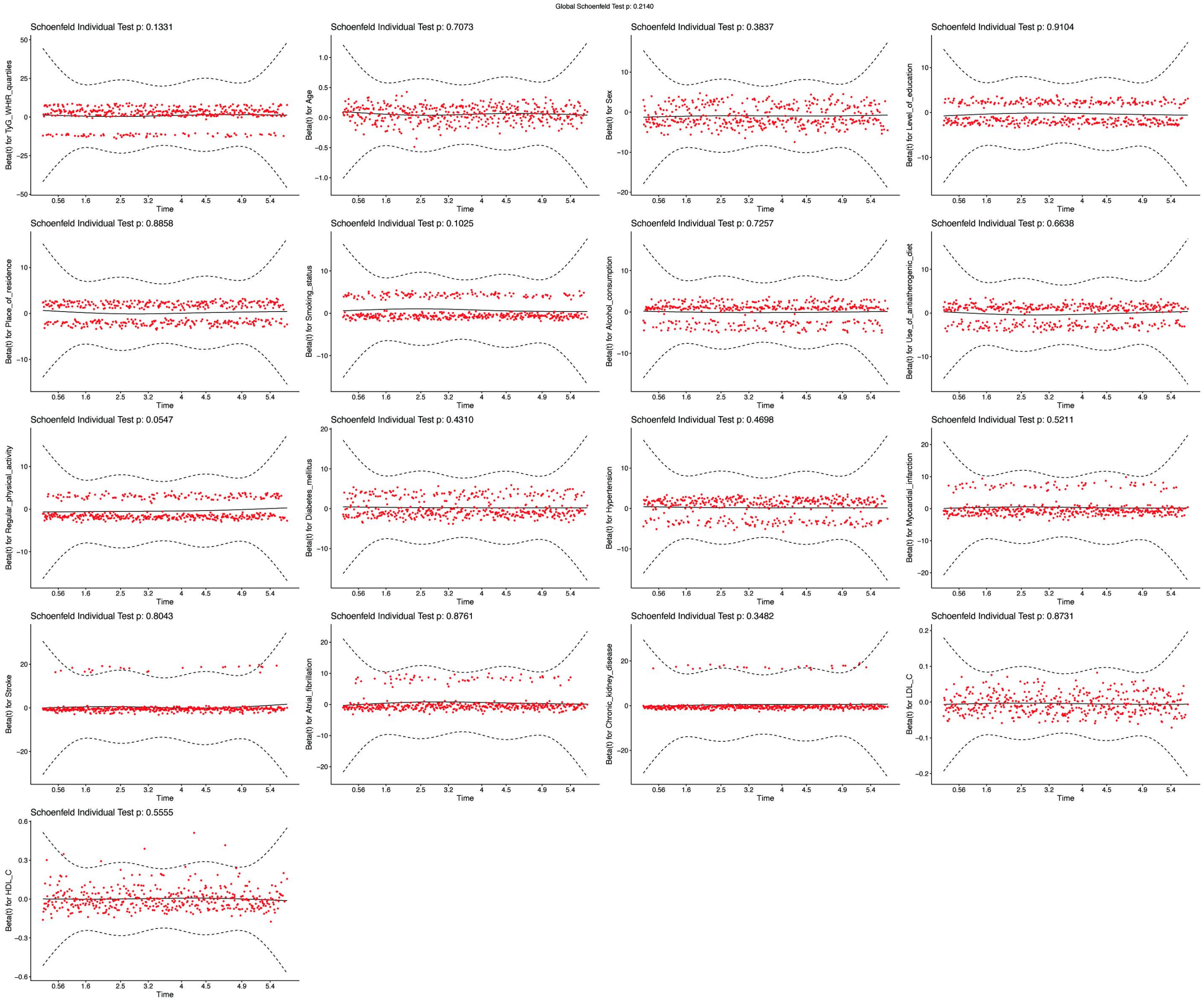

Supplementary Figure S11. Schoenfeld residuals plot for proportional hazards assumption in Cox models (TyG-WHtR and premature all-cause mortality). HDL_C, high-density lipoprotein cholesterol; LDL_C, low-density lipoprotein cholesterol; TyG, triglyceride-glucose index; WHtR, waist-to-height ratio.

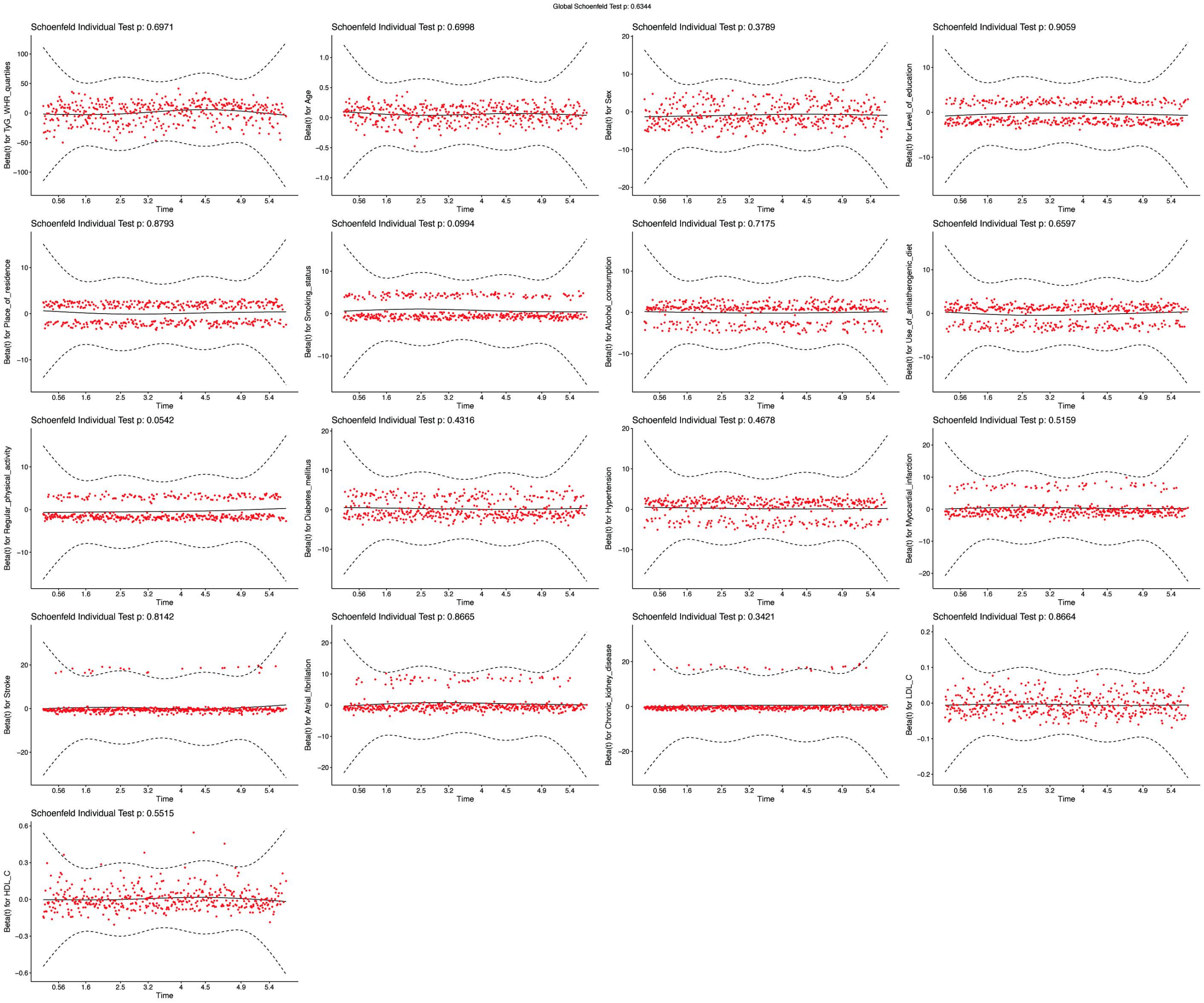

Supplementary Figure S12. Schoenfeld residuals plot for proportional hazards assumption in Cox models (TyG-WHR and premature all-cause mortality). HDL_C, high-density lipoprotein cholesterol; LDL_C, low-density lipoprotein cholesterol; TyG, triglyceride-glucose index; WHR, waist-to-hip ratio.

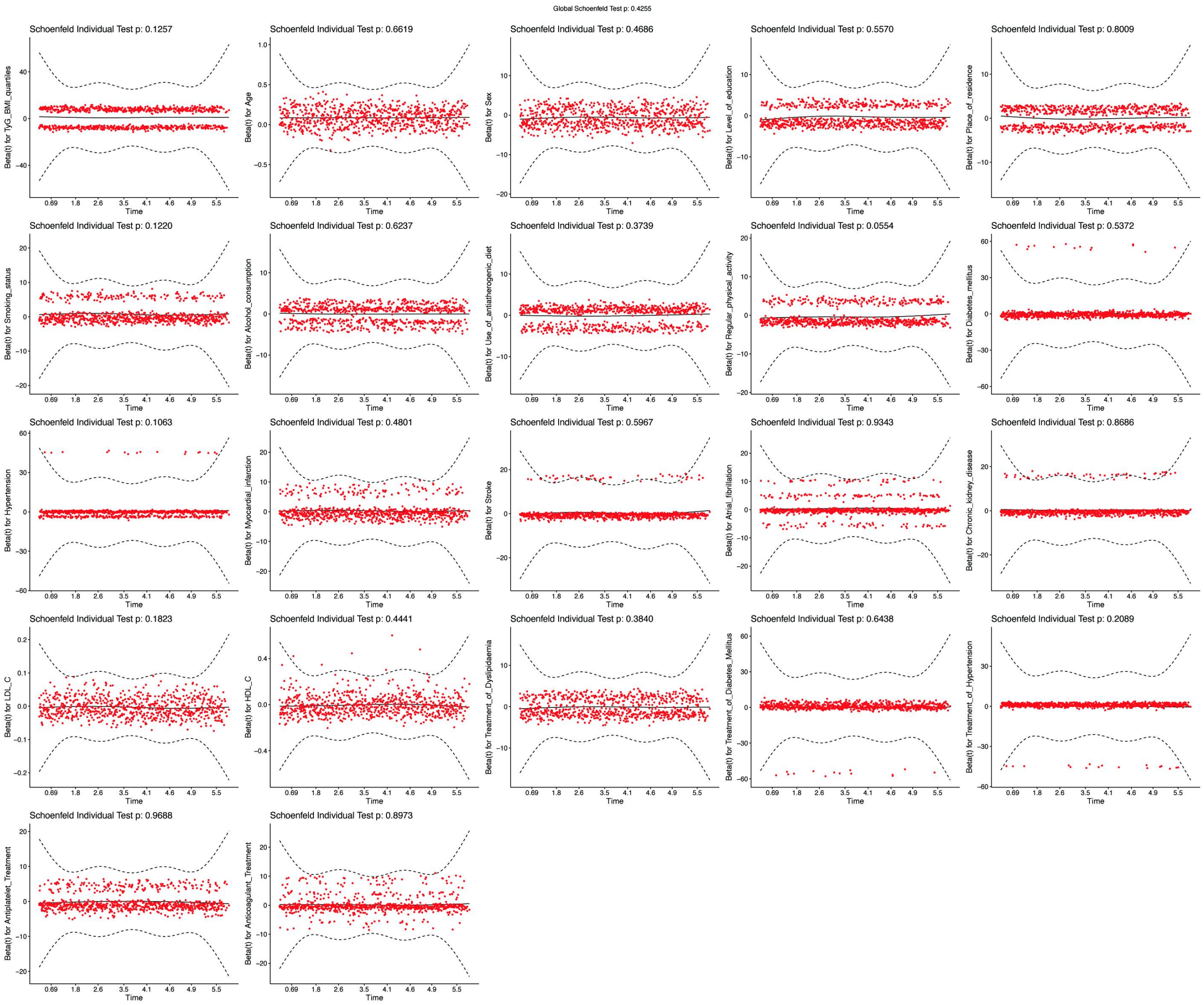

Supplementary Figure S13. Schoenfeld residuals plot for proportional hazards assumption in Cox models of sensitivity analysis (TyG-BMI and total all-cause mortality). BMI, body mass index; HDL_C, high-density lipoprotein cholesterol; LDL_C, low-density lipoprotein cholesterol; TyG, triglyceride-glucose index.

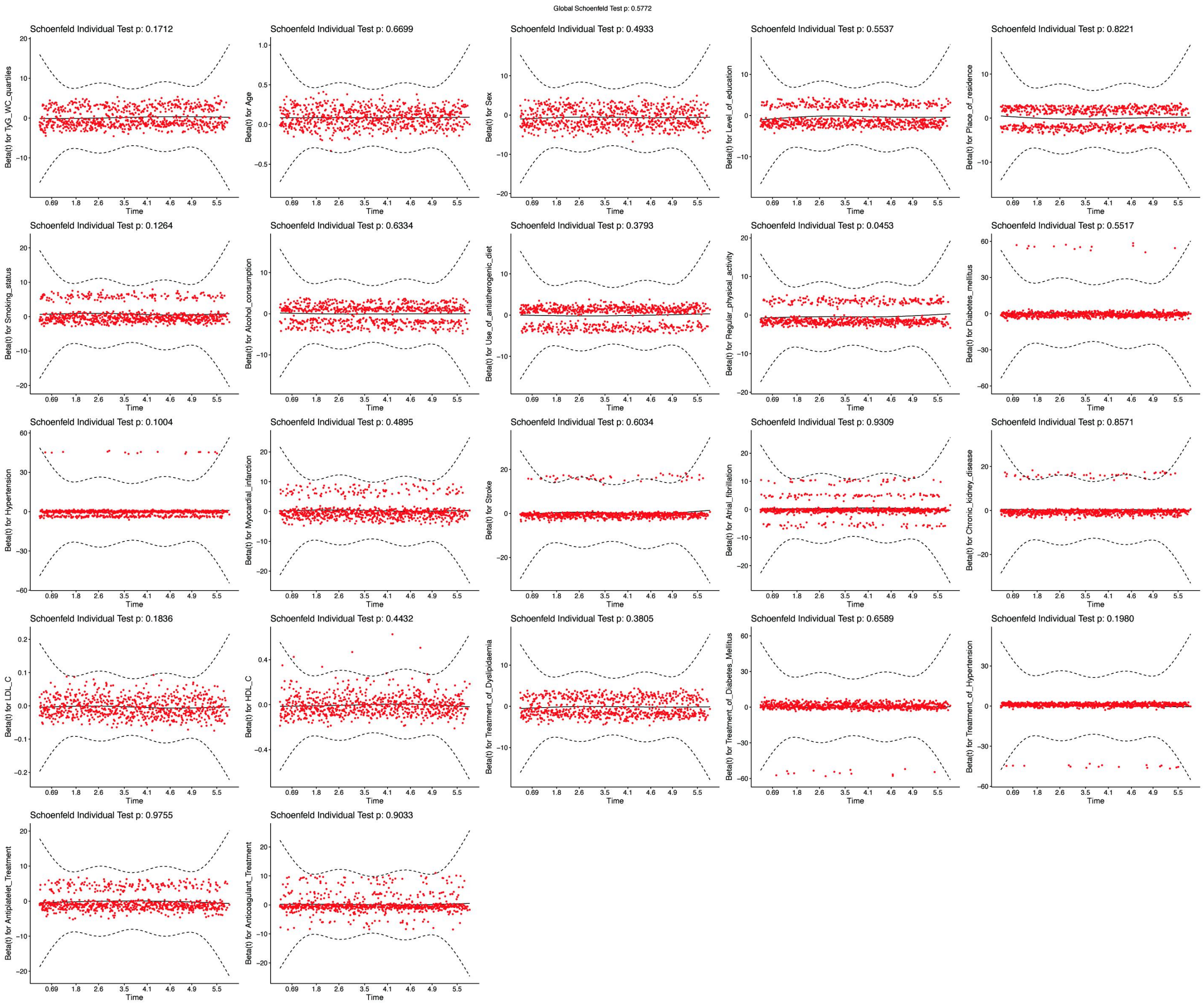

Supplementary Figure S14. Schoenfeld residuals plot for proportional hazards assumption in Cox models of sensitivity analysis (TyG-WC and total all-cause mortality). HDL_C, high-density lipoprotein cholesterol; LDL_C, low-density lipoprotein cholesterol; TyG, triglyceride-glucose index; WC, waist circumference.

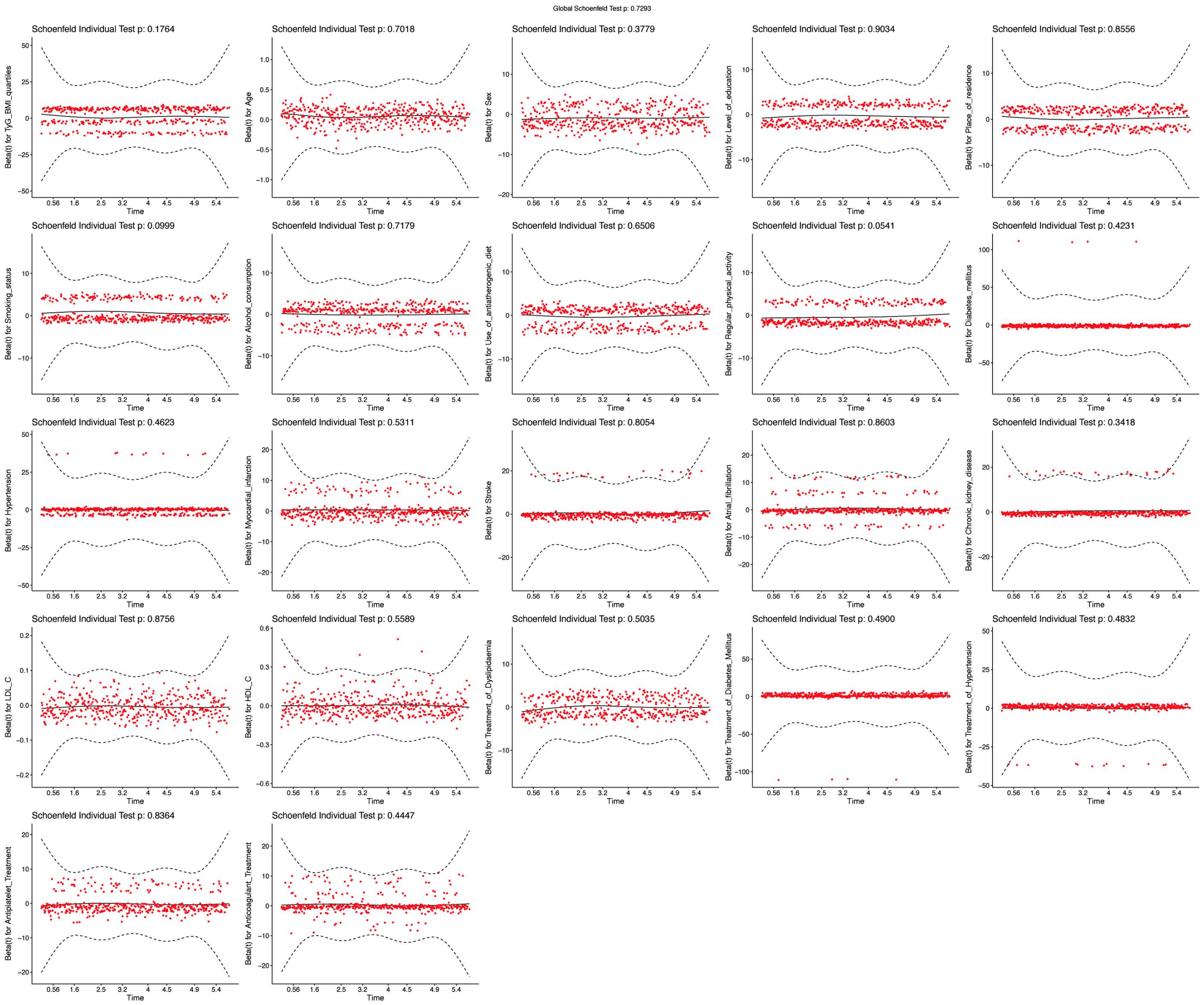

Supplementary Figure S15. Schoenfeld residuals plot for proportional hazards assumption in Cox models of sensitivity analysis (TyG-BMI and premature all-cause mortality). HDL_C, high-density lipoprotein cholesterol; LDL_C, low-density lipoprotein cholesterol; TyG, triglyceride-glucose index; WHR, waist-to-hip ratio.

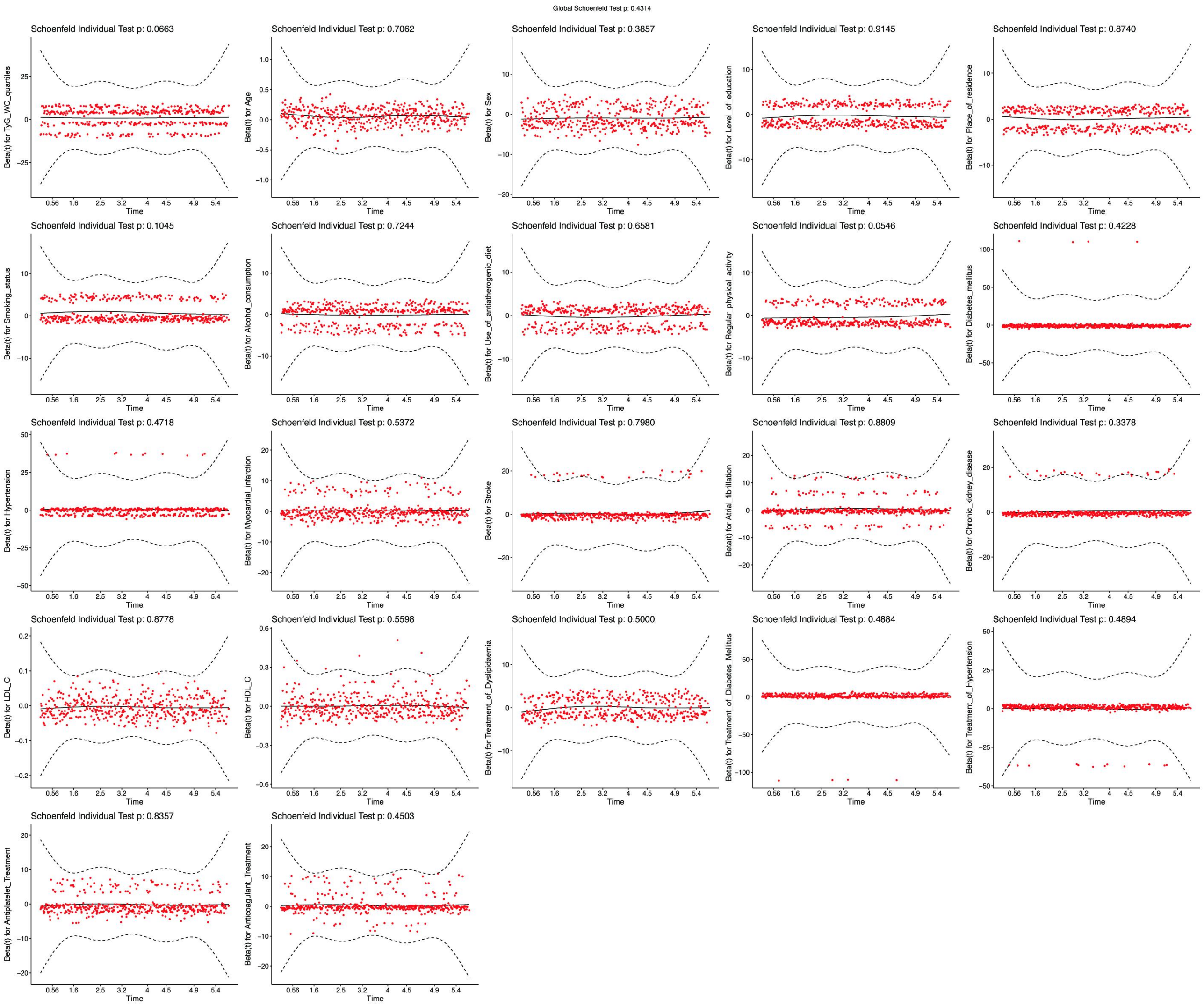

Supplementary Figure S16. Schoenfeld residuals plot for proportional hazards assumption in Cox models of sensitivity analysis (TyG-WC and premature all-cause mortality). HDL_C, high-density lipoprotein cholesterol; LDL_C, low-density lipoprotein cholesterol; TyG, triglyceride-glucose index; WC, waist circumference.

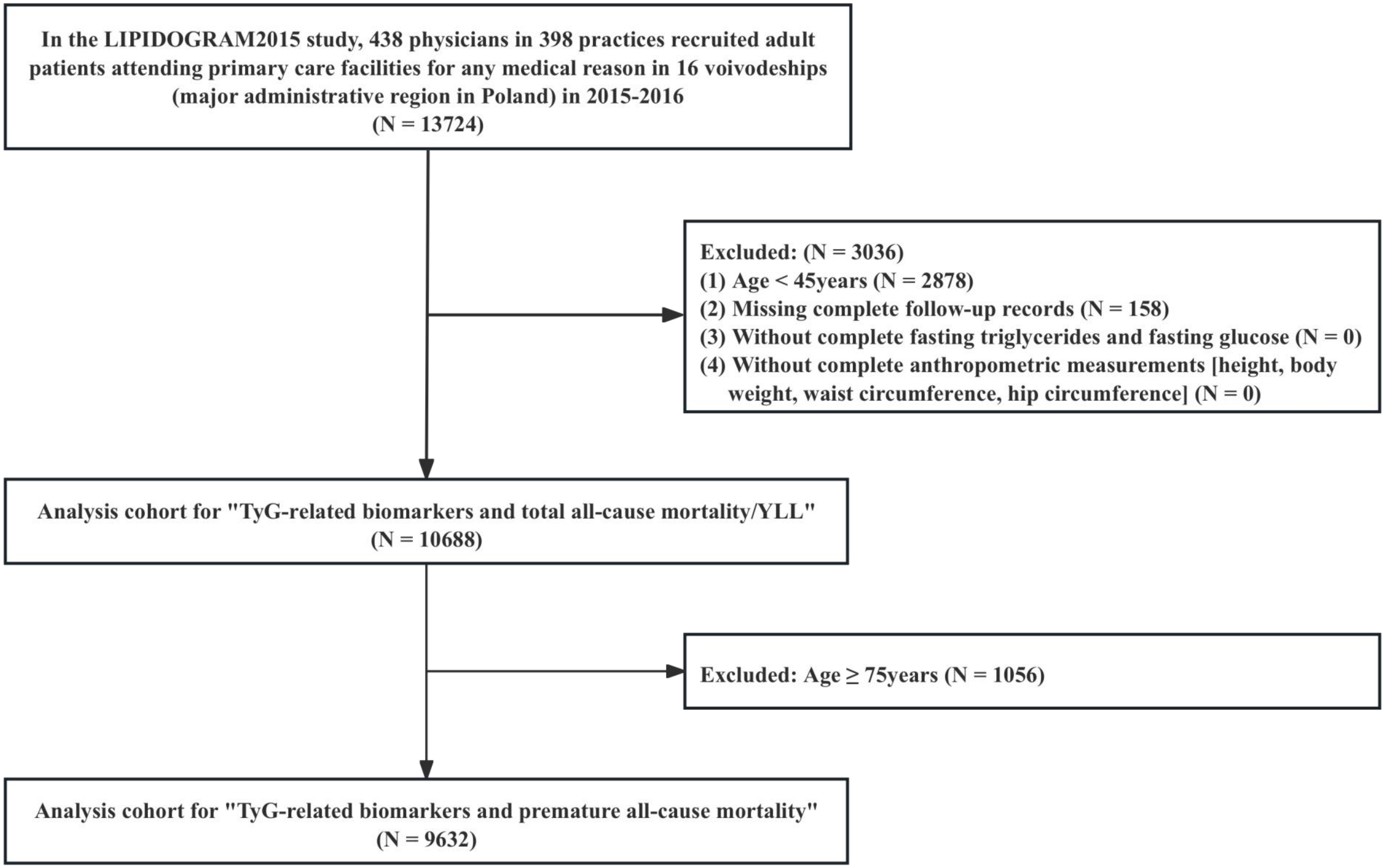

Supplementary Figure S17. Flowchart of this study. TyG, triglyceride-glucose index; YLL, years of life lost.

	Subgroup		HR (95% CI)	P-value	P for interaction
TyG	Male	Q1	0.91 (0.68, 1.22)	0.5181	
		Q2	Reference		
		Q3	0.96 (0.73, 1.26)	0.7519	
		Q4	0.95 (0.72, 1.25)	0.7081	
					0.7022
	Female	Q1	1.19 (0.87, 1.64)	0.2826	
		Q2	Reference		
		Q3	1.09 (0.81, 1.47)	0.5778	
		Q4	1.08 (0.78, 1.50)	0.6383	
TyG-BMI	Male	Q1	1.03 (0.77, 1.39)	0.8297	
		Q2	Reference		
		Q3	1.00 (0.76, 1.32)	0.9934	
		Q4	1.20 (0.91, 1.60)	0.2008	
					0.0355
	Female	Q1	1.88 (1.35, 2.61)	< 0.0001	
		Q2	Reference		
		Q3	1.05 (0.74, 1.49)	0.7721	
		Q4	1.38 (0.99, 1.93)	0.0576	
TyG-WC	Male	Q1	1.47 (1.00, 2.16)	0.0478	
		Q2	Reference		
		Q3	1.23 (0.92, 1.66)	0.1642	
		Q4	1.37 (1.03, 1.83)	0.0318	
					0.7332
	Female	Q1	1.44 (1.06, 1.95)	0.0197	
		Q2	Reference		
		Q3	0.94 (0.69, 1.30)	0.7254	
		Q4	1.20 (0.86, 1.65)	0.2813	
TyG-WHtR	Male	Q1	1.16 (0.83, 1.62)	0.3799	
		Q2	Reference		
		Q3	1.31 (1.00, 1.73)	0.0504	
		Q4	1.25 (0.94, 1.66)	0.1204	
					0.1852
	Female	Q1	1.53 (1.09, 2.13)	0.0129	
		Q2	Reference		
		Q3	0.95 (0.68, 1.32)	0.7599	
		Q4	1.03 (0.75, 1.43)	0.8480	
TyG-WHR	Male	Q1	Reference		
		Q2	1.28 (0.79, 2.09)	0.3209	
		Q3	1.21 (0.76, 1.93)	0.4208	
		Q4	1.25 (0.79, 1.99)	0.3478	
					0.3306
	Female	Q1	Reference		
		Q2	0.82 (0.61, 1.10)	0.1817	
		Q3	0.91 (0.67, 1.24)	0.5478	
		Q4	1.09 (0.76, 1.57)	0.6431	

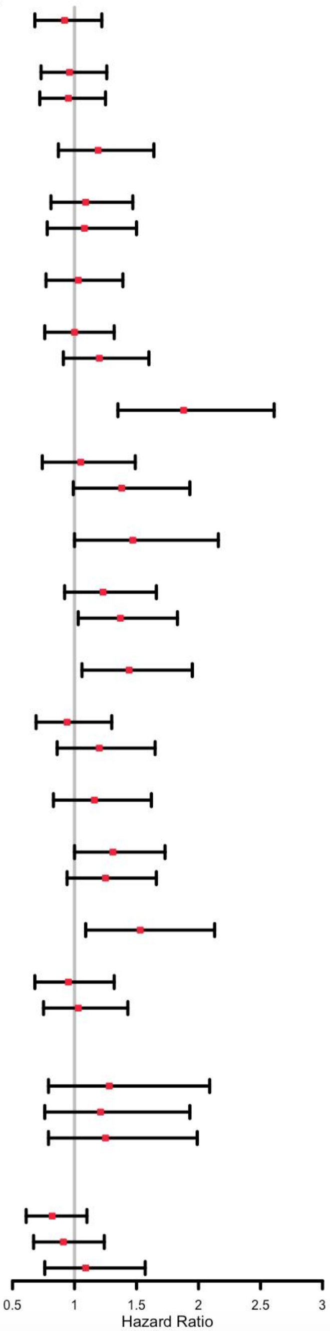

Supplementary Figure S18. Multivariable Cox proportional hazard regression model for total all-cause mortality in male and female. The Cox proportional hazards model was adjusted for age, level of education, place of residence, smoking status, alcohol consumption, regular physical activity, use of antiatherogenic diet, diabetes mellitus, hypertension, chronic kidney disease, myocardial infarction, stroke, atrial fibrillation, low-density lipoprotein cholesterol, and high-density lipoprotein cholesterol. Abbreviations: BMI, body mass index; CI, confidence interval; HR, hazard ratio; TyG, triglyceride-glucose index; WC, waist circumference; WHtR, waist-to-height ratio; WHR, waist-to-hip ratio.

Index	Subgroup	Quartile	HR (95% CI)	P-value	P for interaction
TyG	45 ≤ Age < 65 years	Q1	0.93 (0.62, 1.40)	0.7351	0.0747
		Q2	Reference		
		Q3	1.29 (0.90, 1.86)	0.1657	
		Q4	0.87 (0.58, 1.28)	0.4729	
	Age ≥ 65 years	Q1	1.08 (0.83, 1.39)	0.5715	
		Q2	Reference		
		Q3	0.92 (0.72, 1.18)	0.5153	
		Q4	1.08 (0.84, 1.39)	0.5628	
TyG-BMI	45 ≤ Age < 65 years	Q1	1.68 (1.12, 2.53)	0.0122	0.3279
		Q2	Reference		
		Q3	1.08 (0.71, 1.64)	0.7249	
		Q4	1.64 (1.10, 2.44)	0.0143	
	Age ≥ 65 years	Q1	1.27 (0.98, 1.65)	0.0680	
		Q2	Reference		
		Q3	1.00 (0.78, 1.29)	0.9751	
		Q4	1.13 (0.88, 1.47)	0.3412	
TyG-WC	45 ≤ Age < 65 years	Q1	1.58 (1.01, 2.47)	0.0434	0.2945
		Q2	Reference		
		Q3	1.28 (0.84, 1.93)	0.2502	
		Q4	1.69 (1.12, 2.54)	0.0118	
	Age ≥ 65 years	Q1	1.44 (1.09, 1.90)	0.0110	
		Q2	Reference		
		Q3	1.03 (0.80, 1.32)	0.8418	
		Q4	1.14 (0.89, 1.46)	0.3147	
TyG-WHtR	45 ≤ Age < 65 years	Q1	1.68 (1.10, 2.57)	0.0175	0.1662
		Q2	Reference		
		Q3	1.61 (1.08, 2.39)	0.0189	
		Q4	1.63 (1.08, 2.45)	0.0194	
	Age ≥ 65 years	Q1	1.26 (0.95, 1.67)	0.1053	
		Q2	Reference		
		Q3	1.00 (0.78, 1.28)	0.9895	
		Q4	1.01 (0.78, 1.29)	0.9651	
TyG-WHR	45 ≤ Age < 65 years	Q1	Reference		0.2984
		Q2	1.10 (0.69, 1.77)	0.6859	
		Q3	1.29 (0.81, 2.08)	0.2885	
		Q4	1.08 (0.65, 1.80)	0.7615	
	Age ≥ 65 years	Q1	Reference		
		Q2	0.88 (0.66, 1.17)	0.3657	
		Q3	0.87 (0.65, 1.15)	0.3246	
		Q4	1.01 (0.75, 1.37)	0.9415	

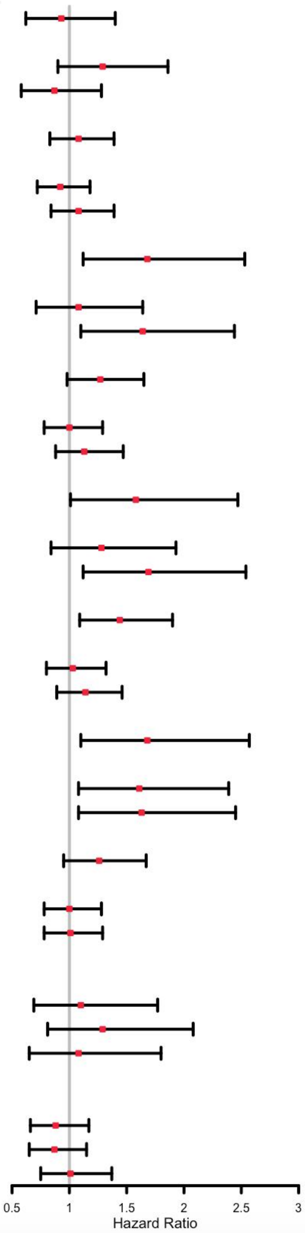

Supplementary Figure S19. Multivariable Cox proportional hazard regression model for total all-cause mortality in two different age groups. The Cox proportional hazards model was adjusted for sex, level of education, place of residence, smoking status, alcohol consumption, regular physical activity, use of antiatherogenic diet, diabetes mellitus, hypertension, chronic kidney disease, myocardial infarction, stroke, atrial fibrillation, low-density lipoprotein cholesterol, and high-density lipoprotein cholesterol. Abbreviations: BMI, body mass index; CI, confidence interval; HR, hazard ratio; TyG, triglyceride-glucose index; WC, waist circumference; WHtR, waist-to-height ratio; WHR, waist-to-hip ratio.

	Subgroup		HR (95% CI)	P-value	P for interaction
TyG	Male	Q1	0.92 (0.63, 1.33)	0.6554	
		Q2	Reference		
		Q3	0.98 (0.70, 1.39)	0.9171	
		Q4	0.94 (0.67, 1.32)	0.7076	
					0.4342
	Female	Q1	0.94 (0.58, 1.53)	0.8132	
		Q2	Reference		
		Q3	1.22 (0.79, 1.87)	0.3738	
		Q4	1.15 (0.72, 1.84)	0.5548	
TyG-BMI	Male	Q1	1.34 (0.90, 1.97)	0.1485	
		Q2	Reference		
		Q3	1.20 (0.84, 1.71)	0.3245	
		Q4	1.54 (1.07, 2.19)	0.0186	
					0.8740
	Female	Q1	1.75 (1.07, 2.85)	0.0263	
		Q2	Reference		
		Q3	1.24 (0.75, 2.05)	0.4133	
		Q4	1.59 (0.97, 2.58)	0.0636	
TyG-WC	Male	Q1	1.91 (1.21, 3.02)	0.0054	
		Q2	Reference		
		Q3	1.01 (0.69, 1.48)	0.9734	
		Q4	1.45 (1.01, 2.10)	0.0455	
					0.0728
	Female	Q1	1.80 (1.11, 2.92)	0.0169	
		Q2	Reference		
		Q3	1.64 (1.01, 2.65)	0.0442	
		Q4	1.73 (1.05, 2.86)	0.0311	
TyG-WHtR	Male	Q1	1.70 (1.13, 2.57)	0.0115	
		Q2	Reference		
		Q3	1.49 (1.05, 2.12)	0.0263	
		Q4	1.51 (1.05, 2.17)	0.0260	
					0.5761
	Female	Q1	1.64 (0.99, 2.71)	0.0559	
		Q2	Reference		
		Q3	1.22 (0.74, 2.02)	0.4347	
		Q4	1.51 (0.92, 2.47)	0.1004	
TyG-WHR	Male	Q1	Reference		
		Q2	0.87 (0.47, 1.59)	0.6411	
		Q3	0.75 (0.42, 1.34)	0.3283	
		Q4	0.83 (0.47, 1.48)	0.5375	
					0.0441
	Female	Q1	Reference		
		Q2	1.03 (0.66, 1.61)	0.8992	
		Q3	1.40 (0.89, 2.21)	0.1502	
		Q4	1.59 (0.94, 2.69)	0.0816	

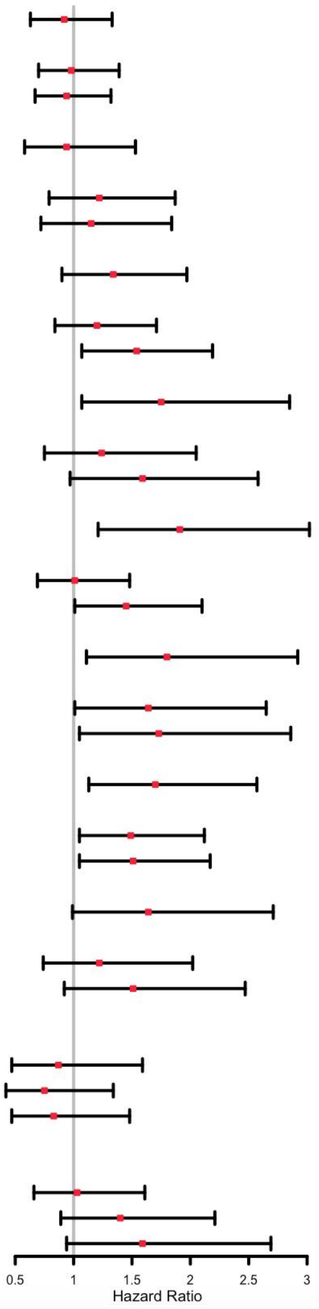

Supplementary Figure S20. Multivariable Cox proportional hazard regression model for premature all-cause mortality in male and female. The Cox proportional hazards model was adjusted for age, level of education, place of residence, smoking status, alcohol consumption, regular physical activity, use of antiatherogenic diet, diabetes mellitus, hypertension, chronic kidney disease, myocardial infarction, stroke, atrial fibrillation, low-density lipoprotein cholesterol, and high-density lipoprotein cholesterol. Abbreviations: BMI, body mass index; CI, confidence interval; HR, hazard ratio; TyG, triglyceride-glucose index; WC, waist circumference; WHtR, waist-to-height ratio; WHR, waist-to-hip ratio.

	Subgroup		HR (95% CI)	P-value	P for interaction
TyG	45 ≤ Age < 65 years	Q1	0.97 (0.65, 1.45)	0.8881	
		Q2	Reference		
		Q3	1.29 (0.90, 1.86)	0.1633	
		Q4	0.91 (0.61, 1.34)	0.6218	
					0.1007
	Age ≥ 65 years	Q1	0.91 (0.59, 1.42)	0.6861	
		Q2	Reference		
		Q3	0.83 (0.55, 1.25)	0.3707	
		Q4	1.09 (0.74, 1.61)	0.6557	
TyG-BMI	45 ≤ Age < 65 years	Q1	1.68 (1.12, 2.53)	0.0121	
		Q2	Reference		
		Q3	1.10 (0.73, 1.67)	0.6403	
		Q4	1.67 (1.12, 2.49)	0.0111	
					0.3880
	Age ≥ 65 years	Q1	1.29 (0.81, 2.03)	0.2841	
		Q2	Reference		
		Q3	1.27 (0.84, 1.92)	0.2504	
		Q4	1.26 (0.83, 1.91)	0.2844	
TyG-WC	45 ≤ Age < 65 years	Q1	1.57 (1.01, 2.45)	0.0475	
		Q2	Reference		
		Q3	1.24 (0.82, 1.88)	0.2992	
		Q4	1.67 (1.11, 2.50)	0.0136	
					0.5968
	Age ≥ 65 years	Q1	2.19 (1.34, 3.55)	0.0016	
		Q2	Reference		
		Q3	1.23 (0.79, 1.90)	0.3665	
		Q4	1.37 (0.89, 2.11)	0.1513	
TyG-WHtR	45 ≤ Age < 65 years	Q1	1.88 (1.22, 2.90)	0.0045	
		Q2	Reference		
		Q3	1.75 (1.16, 2.62)	0.0071	
		Q4	1.75 (1.15, 2.65)	0.0090	
					0.3675
	Age ≥ 65 years	Q1	1.57 (0.98, 2.53)	0.0630	
		Q2	Reference		
		Q3	1.11 (0.74, 1.68)	0.6120	
		Q4	1.24 (0.83, 1.85)	0.2994	
TyG-WHR	45 ≤ Age < 65 years	Q1	Reference		
		Q2	1.08 (0.67, 1.73)	0.7578	
		Q3	1.27 (0.79, 2.05)	0.3177	
		Q4	1.07 (0.64, 1.78)	0.8031	
					0.2580
	Age ≥ 65 years	Q1	Reference		
		Q2	0.97 (0.57, 1.65)	0.9142	
		Q3	0.96 (0.56, 1.62)	0.8630	
		Q4	1.27 (0.74, 2.19)	0.3893	

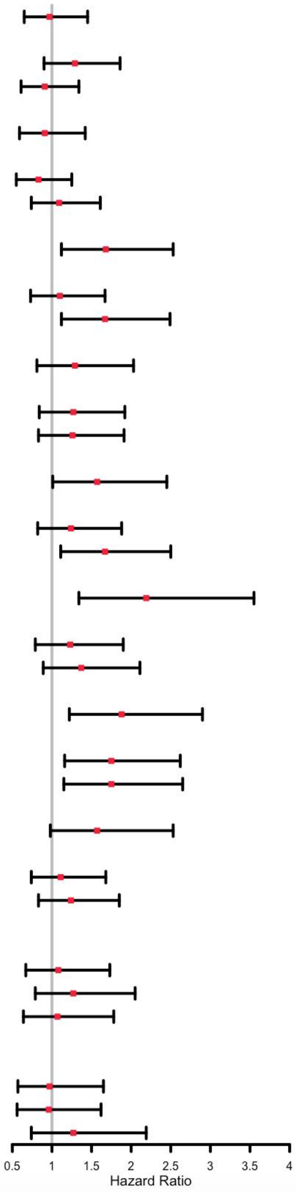

Supplementary Figure S21. Multivariable Cox proportional hazard regression model for premature all-cause mortality in two different age groups. The Cox proportional hazards model was adjusted for sex, level of education, place of residence, smoking status, alcohol consumption, regular physical activity, use of antiatherogenic diet, diabetes mellitus, hypertension, chronic kidney disease, myocardial infarction, stroke, atrial fibrillation, low-density lipoprotein cholesterol, and high-density lipoprotein cholesterol. Abbreviations: BMI, body mass index; CI, confidence interval; HR, hazard ratio; TyG, triglyceride-glucose index; WC, waist circumference; WHtR, waist-to-height ratio; WHR, waist-to-hip ratio.

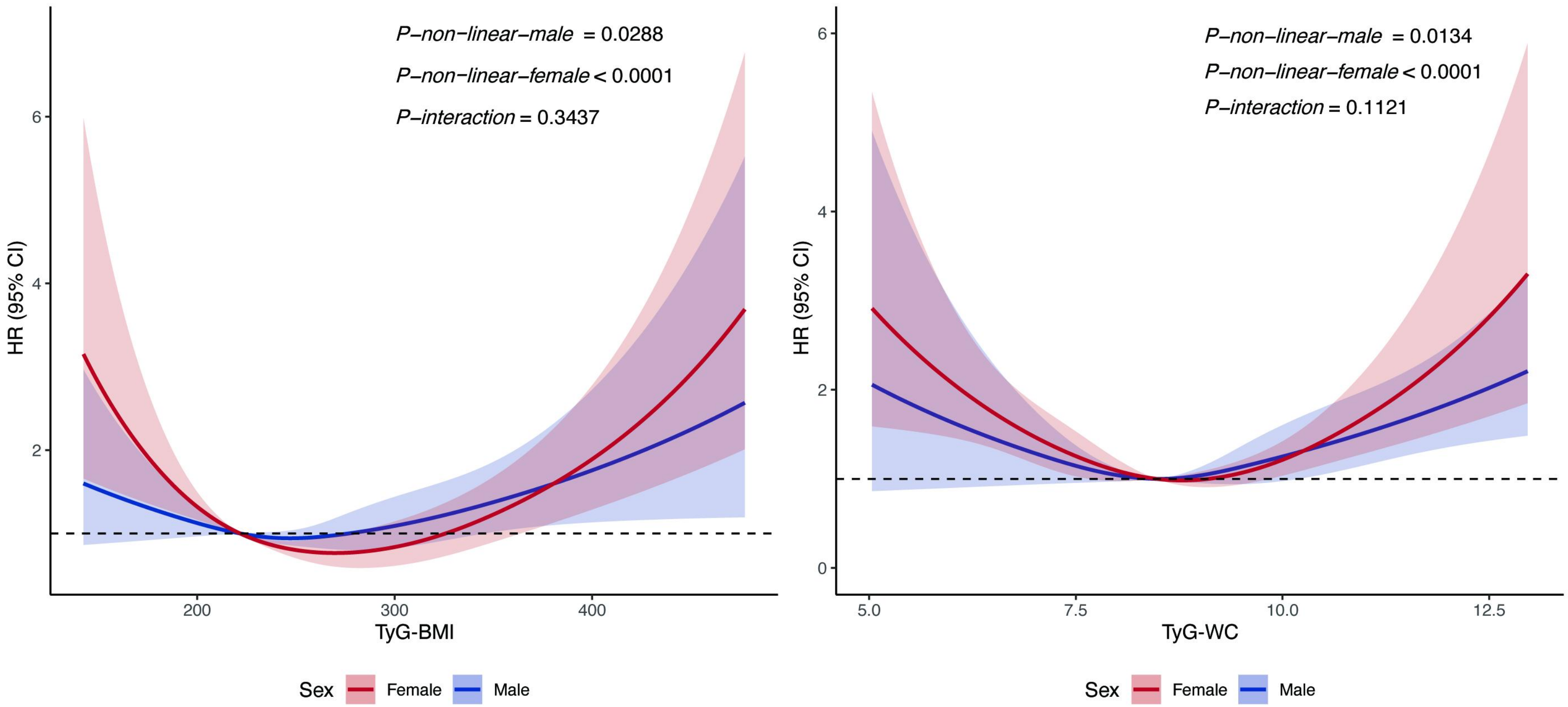

Supplementary Figure S22. Association between TyG-BMI and TyG-WC with total all-cause mortality in males and females using restricted cubic spline analysis. The Cox proportional hazards model was adjusted for age, sex, level of education, place of residence, smoking status, alcohol consumption, regular physical activity, use of antiatherogenic diet, diabetes mellitus, hypertension, chronic kidney disease, myocardial infarction, stroke, atrial fibrillation, low-density lipoprotein cholesterol, and high-density lipoprotein cholesterol. Abbreviations: BMI, body mass index; CI, confidence interval; HR, hazard ratio; TyG, triglyceride-glucose index; WC, waist circumference.
